# The Genome and Development-Dependent Transcriptomes of *Pyronema confluens*: A Window into Fungal Evolution

**DOI:** 10.1371/journal.pgen.1003820

**Published:** 2013-09-19

**Authors:** Stefanie Traeger, Florian Altegoer, Michael Freitag, Toni Gabaldon, Frank Kempken, Abhishek Kumar, Marina Marcet-Houben, Stefanie Pöggeler, Jason E. Stajich, Minou Nowrousian

**Affiliations:** 1 Lehrstuhl für Allgemeine und Molekulare Botanik, Ruhr-Universität Bochum, Bochum, Germany; 2 Center for Genome Research and Biocomputing, Department of Biochemistry and Biophysics, Oregon State University, Corvallis, Oregon, United States of America; 3 Centre for Genomic Regulation (CRG), Barcelona, Spain; 4 Universitat Pompeu Fabra (UPF), Barcelona, Spain; 5 Abteilung Botanische Genetik und Molekularbiologie, Botanisches Institut und Botanischer Garten, Christian-Albrechts-Universität zu Kiel, Kiel, Germany; 6 Institute of Microbiology and Genetics, Department of Genetics of Eukaryotic Microorganisms, Georg-August University, Göttingen, Germany; 7 Department of Plant Pathology and Microbiology, University of California Riverside, Riverside, California, United States of America; MicroTrek Incorporated, United States of America

## Abstract

Fungi are a large group of eukaryotes found in nearly all ecosystems. More than 250 fungal genomes have already been sequenced, greatly improving our understanding of fungal evolution, physiology, and development. However, for the Pezizomycetes, an early-diverging lineage of filamentous ascomycetes, there is so far only one genome available, namely that of the black truffle, *Tuber melanosporum*, a mycorrhizal species with unusual subterranean fruiting bodies. To help close the sequence gap among basal filamentous ascomycetes, and to allow conclusions about the evolution of fungal development, we sequenced the genome and assayed transcriptomes during development of *Pyronema confluens*, a saprobic Pezizomycete with a typical apothecium as fruiting body. With a size of 50 Mb and ∼13,400 protein-coding genes, the genome is more characteristic of higher filamentous ascomycetes than the large, repeat-rich truffle genome; however, some typical features are different in the *P. confluens* lineage, e.g. the genomic environment of the mating type genes that is conserved in higher filamentous ascomycetes, but only partly conserved in *P. confluens*. On the other hand, *P. confluens* has a full complement of fungal photoreceptors, and expression studies indicate that light perception might be similar to distantly related ascomycetes and, thus, represent a basic feature of filamentous ascomycetes. Analysis of spliced RNA-seq sequence reads allowed the detection of natural antisense transcripts for 281 genes. The *P. confluens* genome contains an unusually high number of predicted orphan genes, many of which are upregulated during sexual development, consistent with the idea of rapid evolution of sex-associated genes. Comparative transcriptomics identified the transcription factor gene *pro44* that is upregulated during development in *P. confluens* and the Sordariomycete *Sordaria macrospora*. The *P. confluens pro44* gene (*PCON_06721*) was used to complement the *S. macrospora pro44* deletion mutant, showing functional conservation of this developmental regulator.

## Author Summary

Fungi are a morphologically and physiologically diverse group of organisms with huge impacts on nearly all ecosystems. In recent years, genomes of many fungal species have been sequenced and have greatly improved our understanding of fungal biology. Ascomycetes are the largest fungal group with the highest number of sequenced genomes; however, for the Pezizales, an early-diverging lineage of filamentous ascomycetes, only one genome has been sequence to date, namely that of the black truffle. While truffles are among the most valuable edible fungi, they have a specialized life style as plant symbionts producing belowground fruiting bodies; thus it is difficult to draw conclusions about basal ascomycetes from one truffle genome alone. Therefore, we have sequenced the genome and several transcriptomes of the basal ascomycete *Pyronema confluens*, which has a saprobic life style typical of many ascomycetes. Comparisons with other fungal genomes showed that *P. confluens* has two conserved mating type genes, but that the genomic environment of the mating type genes is different from that of higher ascomycetes. We also found that a high number of orphan genes, i.e. genes without homologs in other fungi, are upregulated during sexual development. This is consistent with rapid evolution of sex-associated genes.

## Introduction

Fungi (Eumycota) are a group of eukaryotes that are present in almost all habitats; therefore they do not only play a great role in nature, but also influence human life in many ways [1]. About 100,000 fungal species have been described, but it is estimated that the actual number might exceed 1.5 million [2]. The largest group among the Eumycota is the Ascomycota (or ascomycetes), which comprise the Saccharomycotina, Taphrinomycotina, and Pezizomycotina. The former groups contain many unicellular species (yeasts) or species that develop only few hyphae or develop hyphae only under certain conditions (dimorphic fungi), whereas the Pezizomycotina are generally filamentous fungi capable of producing highly differentiated multicellular structures, the most complex of which are fruiting bodies for the protection and dispersal of sexual spores [3], [4]. The most basal groups of Pezizomycotina are the Pezizomycetes and the Orbiliomycetes that form open fruiting bodies called apothecia with exposed meiosporangia (asci). Phylogenetically derived groups (e.g. Sordariomycetes, Eurotiomycetes and Dothideomycetes) mostly differentiate closed fruiting bodies where the asci develop within and protected by mycelial structures [3], [5]–[8].

In the last decade, genomes of many filamentous ascomycetes have been sequenced and are invaluable for the analysis of the evolution of species as well as for understanding physiological and morphological properties of fungi. In fact, fungi are among the groups of eukaryotes with the highest number of sequenced genomes to date (http://www.ncbi.nlm.nih.gov/genome/browse/), largely because they include many model organisms, species of medical, agricultural or biotechnological importance. In addition, they usually have compact genomes with short introns and relatively few repetitive regions or non-coding DNA compared to plants and animals, thus making genomic analysis less complex. However, while there are at least ten genome sequences available for each of the more derived groups (Sordariomycetes, Leotiomycetes, Eurotiomycetes and Dothideomycetes), only one Orbiliomycete and one Pezizomycete genome have been sequenced, namely those of a nematode-trapping fungus, *Arthrobotrys oligospora*, and the black truffle, *Tuber melanosporum*, respectively [9], [10]. *A. oligospora* (teleomorph *Orbilia auricolor* [11]) belongs to a group of nematode-trapping soil fungi that comprises only a few known species, which are mostly analyzed for their ability to develop specialized trapping structures, while fruiting body formation is not well studied in this group. The 40 Mb genome of *A. oligospora* encodes ∼11,500 protein-coding genes, similar to the size and coding capacity of other ascomycete genomes [10]. In contrast, the 125 Mb genome of *T. melanosporum* is much larger than those of other sequenced ascomycetes, but contains fewer protein-coding genes. This genome expansion is mostly due to a large number of transposable elements that make up 58% of the truffle genome [9]. Truffles are symbiotic fungi that form mycorrhizal interactions with plant roots; and it has been noted that a biotrophic life-style, either as symbiont or pathogen, is often correlated with an increase in genome size, e.g. caused by repetitive sequences, in many fungi [12]. Furthermore, truffles have a highly specialized fruiting body that is adapted to growth within the soil, in contrast to fruiting bodies of almost all other filamentous fungi, which develop above ground. Thus, even though the truffle genome is of great interest for both ecological and economic reasons, it is difficult to distinguish between features that are ancestral with respect to the filamentous ascomycete lineage, specifically with respect to fruiting body formation, versus features that are adaptations to the truffle-specific life style, i.e. adaptations to mycorrhizal symbiosis or to below-ground fruiting body development. Consequently, the genome sequence of another member of the Pezizomycetes with fruiting body development that is more typical of filamentous ascomycetes will be of great value for evolutionary and morphogenetic analyses. To fill this gap, we sequenced and analyzed the genome and development-dependent transcriptomes of the Pezizomycete *Pyronema confluens*.

**Figure 1:**
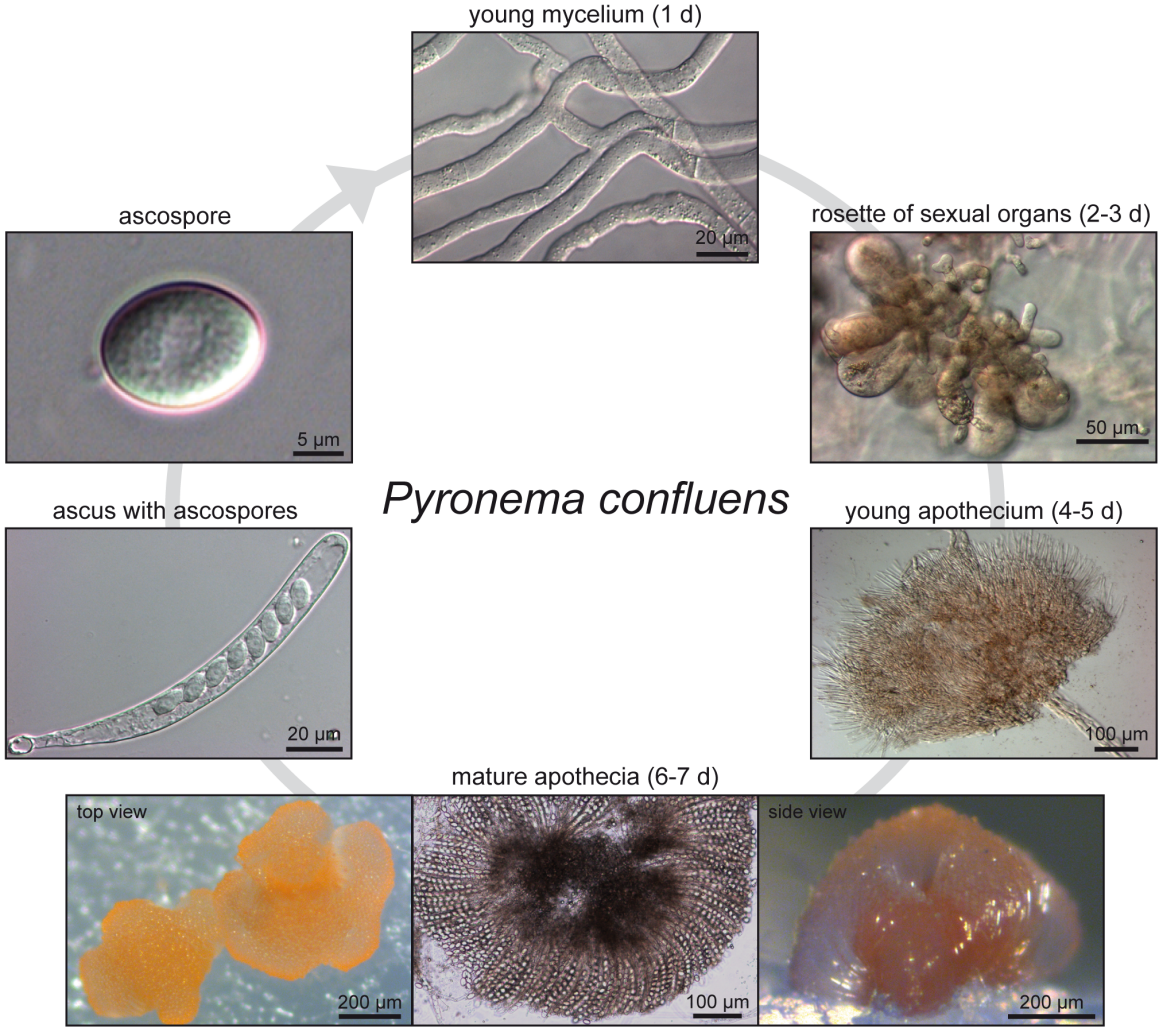
Life cycle of *P. confluens* under continuous illumination and laboratory conditions. Non-pigmented, regularly septated mycelium germinates from haploid ascospores after a few hours. First orange structures of sexual organs can be observed after two days, they consists of ascogonia (enlarged, cytoplasm-rich female structures) and antheridia (male organs). Cytoplasmic fusion and transfer of nuclei are realized by a trichogyne that grows from an ascogonium towards an antheridium; the formation of dikaryotic ascogenous hyphae begins after plasmogamy and is followed by karyogamy and meiosis (not shown). After the fourth day of incubation, young pigmented apothecia can be observed; these contain many thin paraphyses, but no mature asci yet. By the sixth to seventh day, apothecia are mature and contain numerous asci (shown in top and side view to the left and right, the middle panel shows a flattened apothecium to visualize the ascus rosette). Each ascus contains eight hyaline ascospores. With the exception of the ascogenous hyphae in which karyogamy occurs leading to a diploid nucleus (which directly undergoes meiosis to yield haploid ascospores), all hyphae, both in vegetative or sexual structures, contain haploid nuclei.

**Figure 2:**
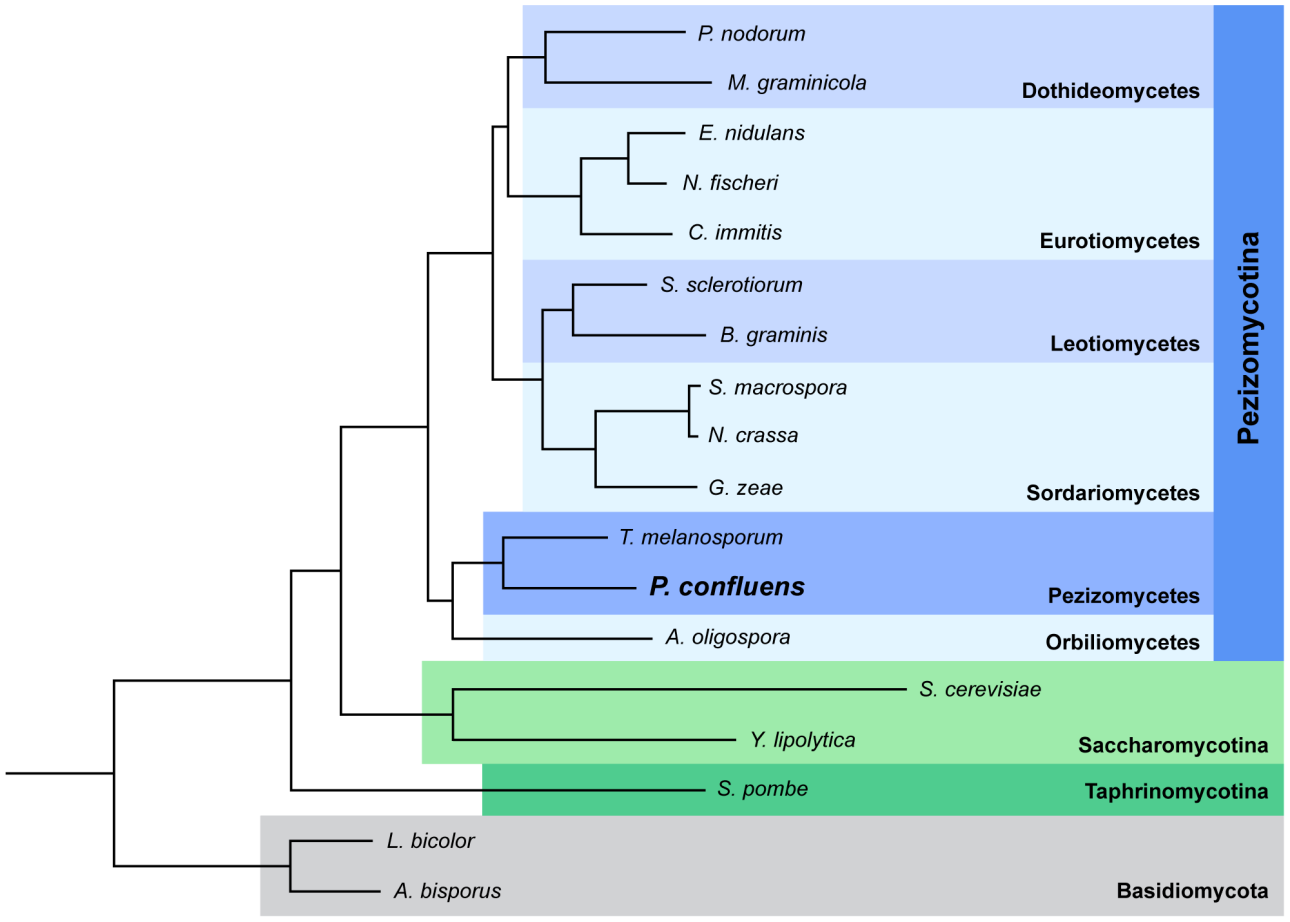
Species tree of 18 fungal species based on phylome reconstruction. The species tree was built using PhyML [191] based on 426 single-copy, widespread genes (see Materials and Methods for details). A bootstrap of 100 repetitions was also reconstructed, bootstrap values for all branches were 100%. A species tree constructed with the super-tree reconstruction program DupTree [195] based on all 6,949 trees reconstructed in the phylome had the same topology. The divergence time of the Pyronema and Tuber lineages was estimated at 260 or 413 Mya using r8s [49] calibrated with divergence times of 723.86 Mya and 1147.78 Mya, respectively, for *Schizosaccharomyces pombe* and the remaining ascomycetes (see Materials and Methods).

*P. confluens* was established as a model organism for the analysis of cell biology and fruiting body development in filamentous ascomycetes during the first half of the 20th century. It was instrumental in the elucidation of the dikaryotic phase during sexual development of higher ascomycetes [13]–[17]. In the last decade, *P. confluens* was used in comparative studies of gene expression during sexual development of ascomycetes [6], [18], [19]. It is a soil-living saprobe found in forests in temperate climates. In nature, its fruiting bodies (apothecia) usually appear on the ground after forest fires [20]. Under laboratory conditions, *P. confluens* has a short life cycle where typical apothecia containing eight-spored asci are formed within six days (Figure 1). This is rare among members of the Pezizomycetes, many of which do not easily reproduce sexually in the laboratory. Also, *P. confluens* is homothallic (self-fertile), therefore no crossing partner of different mating type is needed for the fungus to complete its sexual cycle [17]. In addition, *P. confluens* can also be used to study the effects of light on fruiting body formation, because in contrast to many other filamentous ascomycetes, fruiting body development in this fungus is strictly light-dependent [6], [21]. A previous analysis based on sequence data from 15 proteins showed that the *P. confluens* lineage is positioned at the base of the filamentous ascomycetes in a phylogenetic tree [6]. Phylogenomic analysis based on the genome data from this study and including sequences from *T. melanosporum* and *A. oligospora* confirms this basal position with the Pezizomycetes as sister group to the Orbiliomycetes (Figure 2).

In two previous small pilot studies, *P. confluens* was used for comparative expression analyses to identify genes with evolutionary conserved expression patterns during fruiting body development in ascomycetes [6], [19]. These studies already indicated that gene expression patterns during development might be conserved even over large evolutionary distances. Here, we sequenced the genome and development-dependent transcriptomes of *P. confluens* with the following objectives: (i) To work towards closing the sequence gap among basal filamentous ascomycetes, and, thus, to learn more about the evolution of fungal genomes. (ii) To use the genome and transcriptome data to study the biology of a basal filamentous ascomycete in comparison with more derived species, with a focus on sexual development.

## Results/Discussion

### Sequencing and Assembly of the *P. confluens* Genome and Development-Dependent Transcriptomes

The genome of the *P. confluens* strain CBS100304 was sequenced with a combination of Roche/454 and Illumina/Solexa sequencing similar to what was described previously for *Sordaria macrospora* [22]. A summary of the sequence reads that were used for the *P. confluens* assembly is given in Table S1. The final assembly consists of 1,588 scaffolds (1,898 contigs) with a total size of 50 Mb, a scaffold N50 of 135 kb and a GC content of 47.8% (Table 1). To estimate the genome size independently of the assembly, k-mer analyses based on the Illumina/Solexa reads were performed using an algorithm described for the potato genome [23]. The analysis resulted in one clear peak, as one would expect for haploid genome (Figure S1). Based on the analysis for different k-mer lengths (31 and 41), a total genome size of ∼50.1 Mb was predicted which is close to the total length of the assembly.

**Table 1:** Main features of the *P. confluens* genome.

assembly size	50 Mb
no. of scaffolds	1,588
N50	135 kb
GC content	47.8%
number of predicted protein-coding genes	13,369
number of predicted tRNAs	605
average length of CDSs	1,093 nt
GC content of CDSs	51.1%
coding regions in genome	29.2%
average length of mRNAs	1,483 nt
GC content of mRNAs	49.9%
mean/median length of 5′ UTRs^1^	260/156 nt
mean/median length of 3′UTRs^1^	281/200 nt

1 only those genes used where a UTR length >0 was determined.

For transcriptomics, we performed RNA-seq for three different conditions: sexual development (sex), long-term culturing in the dark (DD), and a mixture of different vegetative tissues (vegmix), in two biological replicates per condition (Table S1). For each condition, RNA from different time points was pooled to represent a high number of genes that are expressed during the corresponding condition (see Materials and Methods for details). RNA for sex samples was extracted from mycelia grown in minimal medium in surface culture in constant light. Only under these conditions is *P. confluens* able to develop fruiting bodies, whereas growth in darkness, submerged, or in complete medium prevents sexual development. We used RNA from 3d, 4d, and 5d old mycelia to cover the initial stages of sexual development up to the development of young fruiting bodies (Figure 1). The DD samples comprised RNAs from mycelia grown in minimal medium in submerged culture in constant darkness, which prevents fruiting body formation. The vegmix samples also contained only RNAs from mycelia that could not develop fruiting bodies, but from a mixture of growth conditions different from the DD condition. We argue that the use of different mycelia sharing the common denominator of “no fruiting bodies” would allow us to focus on genes that are differentially expressed during fruiting body morphogenesis by comparing the three different conditions. Thus, genes that are differentially regulated in the comparisons sex/DD and sex/vegmix, but not DD/vegmix are candidates for genes that are regulated in a sexual development-dependent manner.

RNAs from different growth conditions were also used to allow a high read coverage of as many genes as possible for annotation purposes as described previously for *Sordaria macrospora* [24]. Therefore, gene model predictions were performed *ab initio* as well as evidence-based on the RNA-seq data (details in Materials and Methods). The output from different gene prediction pipelines was merged using MAKER [25]. Gene model predictions were scanned for consistency, and ∼10% of the predicted gene annotations were corrected manually to improve the exon/intron structure. To address the question whether the assembly and annotation cover the complete gene space of *P. confluens*, we performed a BLASTP search with a eukaryotic core gene set as previously described [26]. All of the 248 single-copy core genes were present in the *P. confluens* predicted peptides, suggesting that the assembly covers the complete core gene space.

Untranslated regions (UTRs) were also modeled by the gene prediction pipeline, and were refined based on the manually curated gene set using custom-made Perl scripts as described previously [24]. For the current assembly, 13,369 protein-coding genes are predicted with an average CDS length of 1,093 and an average transcript length of 1,483 bases (Table 1, Table S2). The median length of 5′ and 3′ UTRs are 156 and 200 bases, respectively, similar to findings in *T. melanosporum*, *Aspergillus oryzae* and *S. macrospora* [24], [27], [28]. Furthermore, we predicted 605 tRNAs, and assembled an rDNA unit comprising the 18S, 5.8S and 28S rRNA genes as well as the internal transcribed spacers (ITS) 1 and 2. Based on this annotation, the majority of the RNA-seq reads map to exonic regions as expected (Figure S2).

Spliced sequence reads identified in RNA-seq mapping results can not only be used to improve the exon/intron structures of predicted genes, but also to address the question of natural antisense transcripts (NATs), because the consensus sequences at the 5′ and 3′ ends of introns allow strand determination even in non-strand-specific RNA-seq data. NATs can play a role in the regulation of gene expression, and were found to be pervasive in metazoans [29], [30]. To identify putative NATs in *P. confluens*, we extracted predicted splice sites in antisense orientation to annotated genes from the mapping results obtained with Tophat [31]. Antisense splice sites covered by at least five spliced sequence reads, and with a coverage of more than 10% of the average coverage of the predicted sense-transcript were checked manually to remove splice sites that were most likely due to annotation errors, within repeat-rich regions, or close to sequence gaps. This yielded 376 antisense splice sites in 281 genes (Table S3), indicating that natural antisense transcripts are present in *P. confluens*. The number of genes with NATs is most likely underestimated, because we set stringent criteria, and non-spliced antisense transcripts could not be discovered by this analysis. In *T. melansporum*, only 33 NATs were identified from RNA-seq data by gene modeling, but this low number most likely also is an underestimate due to the method used [27]. Few studies have addressed the presence of NATs at a genome-wide scale in filamentous fungi, and the number of NATs that were identified in the ascomycetes *Aspergillus flavus*, *Aspergillus niger*, and *Magnaporthe grisea* [32]–[34], and in the basidiomycetes *Ustilago maydis*, *Coprinopsis cinerea*, and *Schizophyllum commune* [35]–[37] is in a range similar to our findings in *P. confluens*. Thus, NATs appear to be present across all groups of filamentous fungi, even though they do not seem to be as pervasive as in metazoans.

When compared with the genome of its closest sequenced relative, *T. melanosporum* [9], the *P. confluens* genome is much smaller (50 Mb versus 125 Mb), but contains nearly twice the number of protein-coding genes (13,369 versus 7,496). The large size of the truffle genome is mostly due to an expansion of transposons and other repeated elements, with nearly 58% (∼71 Mb) of the genome consisting of repeats larger than 200 bp [9]. Repeat analyses based on similarity to known repeat classes as well as *de novo* repeat finding showed that in *P. confluens*, transposable elements longer than 200 bp constitute only 12% (∼6 Mb) of the genome, with low complexity regions and simple repeats constituting an additional 0.21 and 1.03%, respectively (Table S4). Very few of the repeats show a high percentage of sequence identity with the repeat consensus sequences, indicating a high degree of divergence among repeats (Figure S3). This is different from findings in *T. melanosporum*, *Fusarium oxysporum*, and *Pyrenophora tritici-repentis* [9], [38], [39], but similar to *Nectria haematococca* [40]. One reason for this finding might be that long perfect repeats (longer than the Roche/454 read lengths of 300–400 bp) were lost in the assembly process; however, this would not apply to shorter perfect repeats. Also, the k-mer analysis showed a single main peak corresponding to a genome size of 50 Mb (Figure S1), and did not show any major extra peaks, indicating that most of the genome sequence is represented in the assembly. Thus, the presence of repeats with dissimilar sequences suggests that the repeats are evolutionary rather old, and that genome defense processes are active in *P. confluens*. A search for genes that might be involved in chromatin modification and other silencing processes showed that *P. confluens* comprises gene sets similar to those in other fungi where genome defense processes were identified. Interestingly, there is even a slight expansion in some families of putative RNA interference genes (Table S5). This suggests that in its current state, the *P. confluens* genome is well-protected against repeat spreading, and that the high repeat content of the *T. melanosporum* genome is not characteristic for all members of the Pezizales. Among the genes that might be involved in genome defense is *PCON_06255*, a homolog of the *N. crassa rid* gene. In *N. crassa*, the cytosine DNA methyltransferase RID is essential for repeat induced point mutation (RIP) [41]. The RIP process introduces C∶G to T∶A mutations in duplicated sequences of more than 400 bp and at least 80% sequence identity during the sexual cycle, and thus is a means to control the spread of duplicated sequences including transposons [42]–[44]. The *rid* homolog *PCON_06255* is slightly upregulated during sexual development (Table S2), consistent with a role for the gene product under these conditions; however, an analysis of the genomic DNA for the presence of regions that might have been subjected to RIP yielded only a small fraction (0.46%) of the genome (Table S6). Thus, RIP does not seem to play a major role in genome defense in *P. confluens*. Overall, with respect to genome size, gene number, and transposon content, the *P. confluens* genome is more similar to those of many higher filamentous ascomycetes than to the truffle genome, indicating that these features in truffle might be a consequence of the specialized life style.

**Figure 3:**
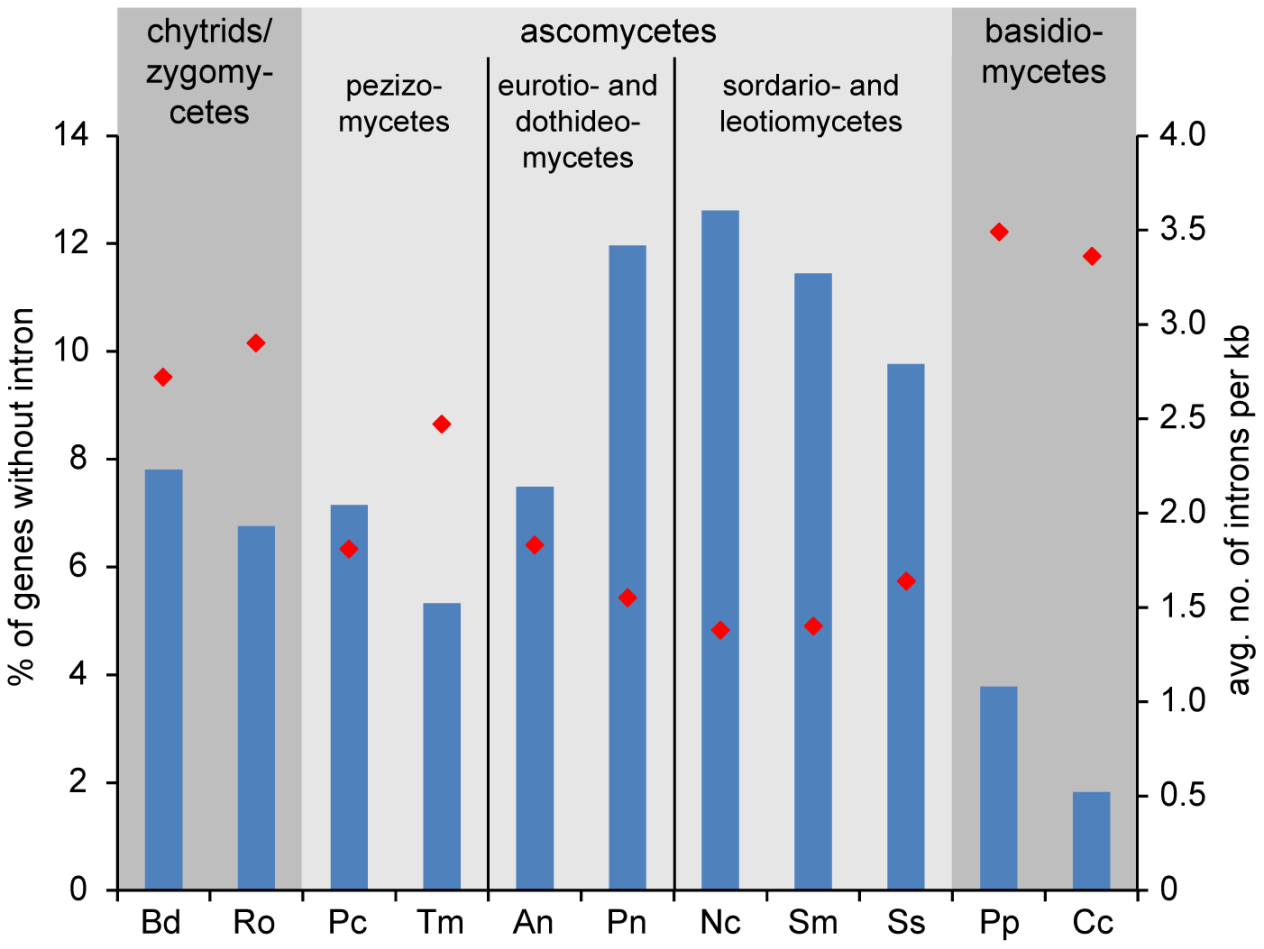
Intron content of protein-coding genes from *P. confluens* and 10 other filamentous fungi. Putative orthologs to *P. confluens* genes were identified by reciprocal BLASTP analysis, and 747 genes with orthologs across all fungal genomes used were analyzed for intron content (analysis of CDSs only, because UTRs are not annotated in all cases). Blue bars give the percentage of genes without introns (left y-axis), red diamonds give the average number of genes per kb (right y-axis). Data from genome projects for the following species were used for the analysis: Bd, *Batrachochytrium dendrobatidis* (http://genome.jgi-psf.org/Batde5/Batde5.home.html); Ro, *Rhizopus oryzae* [207]; Pc, *Pyronema confluens* (this study); Tm, *Tuber melanosporum* [9]; An, *Aspergillus nidulans* [174]; Pn, *Phaesphaeria nodorum* [179]; Nc, *Neurospora crassa* [178]; Sm, *Sordaria macrospora* [22]; Ss, *Sclerotinia sclerotiorum* [182]; Pp, *Postia placenta* [208]; Cc, *Coprinopsis cinerea* [209].

It has been shown previously that the number of introns per gene varies greatly between different fungal lineages, with filamentous ascomycetes harboring one to two introns per kilobase [45]. However, at the time of the study, no Pezizomycete genome sequences were available, therefore we performed an analysis of intron content in *P. confluens* and *T. melanosporum* in comparison with nine other filamentous fungi representing major fungal lineages (Figure 3). The comparison included 747 genes for which orthologs could be identified in all analyzed fungi. About 7% of the analyzed *P. confluens* genes do not contain introns, similar to chytrids and zygomycetes, and less than in most higher ascomycetes where 10–12% of the investigated genes are intron-free. This is also much less than in hemiascomycete yeasts, e.g. *S. cerevisiae*, where only a minority of genes contains introns [45]. However, it is almost twice the number found in basidiomycetes, where less than 4% of the analyzed genes do not contain introns. The average number of introns per kb is 1.83 in *P. confluens*, which is larger than that of the higher filamentous ascomcyetes with the exception of *Aspergillus nidulans*, but still in the same range as found in the previous study [45]. Interestingly, *T. melanosporum* has 2.49 introns per kb, the highest value of all investigated ascomycetes. These data might indicate that within filamentous ascomcyetes there is a tendency towards net intron loss that is more pronounced in the evolutionary derived lineages than in the basal Pezizomycetes. However, the exceptionally high intron content of *T. melanosporum* might also be life style-specific or connected to the high repeat content in this fungus.

The availability of transcriptome data for *P. confluens* also allowed us to compare overall expression levels across the genome. In a previous study with metazoans, genes could be grouped in two classes with high and low expression levels, respectively, independent of species, tissue type, or type of experiment [46]. In fungi, this has been addressed only in the filamentous ascomycete *Sordaria macrospora* where the situation is different, because there were up to three expression peaks depending on the conditions analyzed [24]. Interestingly, the situation *in P. confluens* is more similar to metazoans, with two main peaks that represent high and low expression in all three conditions investigated (for details see Text S1 and Figure S4).

### Analysis of Synteny with Other Fungal Species

**Figure 4:**
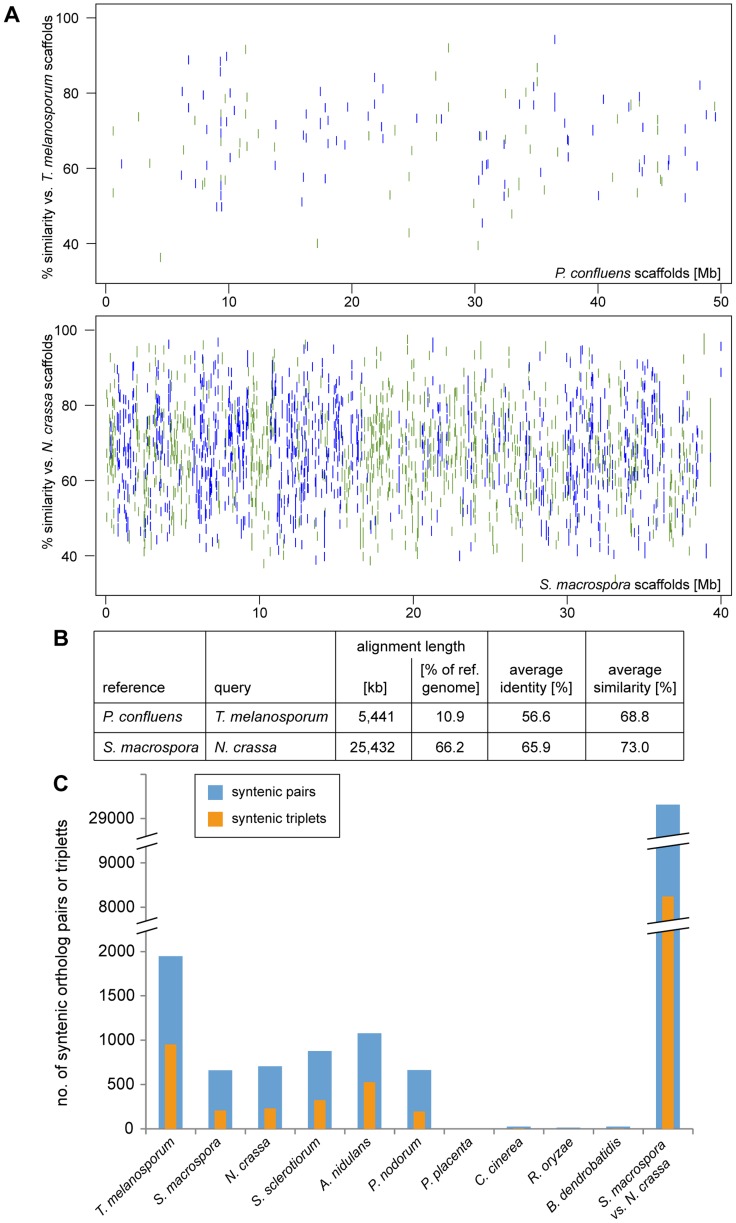
Synteny analysis with other fungi. **A–B.** Regions of sequence identity between the *in silico*-translated genomic sequences of the pairs *T. melanosporum*/*P. confluens* and *S. macrospora*/*N. crassa* (for comparison) were determined with the PROmer algorithm from the MUMmer package [47]. The percent identity plot (A) was plotted with gnuplot, green indicates sequences on the forward strand, blue on the reverse strand of the reference. The PROmer analysis shows a much higher percentage of regions of similarity/identity between *S. macrospora* and *N. crassa* than between *T. melanosporum* and *P. confluens*. **C.** The number of pairs or triplets of orthologous genes within a 20 kb (pairs) or 40 kb (triplets) region was determined for *P. confluens* and 10 other fungi. For comparison, the number of syntenic pairs or triplets was also determined for *S. macrospora* and *N. crassa* (bars at the right). Note that the y-axis is interrupted in two places for better visualization. Data used for this analysis are from the genome projects as indicated in the legend of Figure 3.

The closest relative of *P. confluens* with a sequenced genome is *T. melanosporum*, therefore, we used the MUMmer package [47] to determine regions of sequence similarity and possible synteny, i.e. the order of genes within the genome, between the two species. However, even though both species belong to the order Pezizales, there is little sequence similarity at nucleic acid level (data not shown), therefore we used the PROmer algorithm from the MUMmer package to compare the *in silico*-translated genomic sequences (Figure 4). Even at amino acid level, only ∼11% of the *P. confluens* genome align with the *T. melanosporum* genome, compared to more than 66% in the highly syntenic genomes of *S. macrospora* and *N. crassa* (Figure 4A and B). A dot plot analysis of the PROmer results also indicated a low degree of overall synteny between *P. confluens* and *T. melanosporum* (Figure S5A). In a second analyis of synteny, we identified orthologs for all *P. confluens* genes in the predicted proteomes of ten filamentous fungi by reciprocal BLAST analysis [48], and used the positions of orthologous proteins on sequenced chromosomes or contigs to determine synteny (Figure S5B). A dot plot for this comparison of *P. confluens* with *T. melanosporum* also shows that there is little overall synteny, in contrast to the comparison of the highly syntenic genomes of *N. crassa* and *S. macrospora*. The low degree of synteny between *P. confluens* and *T. melanosporum* might be explained by their large evolutionary distance. Estimation of divergence times of the Pyronema and Tuber lineages using r8s [49] placed their most recent common ancestor at least 260 Mya (million years ago), nearly twice the time of the estimated divergence of Tuber from its sister groups within the Tuberaceae (∼156 Mya, [50]).

Nevertheless, an analysis of the number of syntenic gene pairs or gene triplets showed that although the number of syntenic pairs and triplets in *P. confluens* versus *T. melanosporum* is still lower than in *N. crassa* versus *S. macrospora*, it is much higher than in comparisons of *P. confluens* with other ascomycetes (Figure 4C). This indicates that many regions of microsynteny exist between *P. confluens* and *T. melanosporum* whereas overall chromosomal synteny was lost. In contrast, in comparisons of *P. confluens* with basidiomycete, zygomycete, or chytrid species, we found few syntenic gene pairs or triplets.

### *P. confluens* Might Reflect an “Intermediate State” in the Evolution of the Genomic Environment of the Mating Type Loci

In filamentous ascomycetes, the master regulators of sexual reproduction are the various genes that reside at the mating type (*MAT*) loci [51]. They encode transcription factors that regulate the sexual cycle. Heterothallic ascomycetes have a bipolar mating type system, with isolates possessing one of two non-allelic versions (idiomorphs) of a single *MAT* locus, termed *MAT1-1* and *MAT1-2* [52]. The *MAT1-1-1* and *MAT1-2-1* genes encode transcription factors with a conserved alpha domain and high-mobility group (HGM)-domain, respectively [51]. Conversely, homothallic ascomycetes carry both *MAT* loci within a single genome, with the two loci either fused together, located within close proximity, or on separate chromosomes [53]–[57]. BLASTP searches with MAT1-1-1 proteins of different filamentous ascomycetes revealed the presence of a *MAT1-1-1* gene (*PCON_07491*, scaffold 329) encoding a putative transcription factor with an alpha domain that is most similar to the MAT1-1-1 protein of *T. melanosporum* [58] (Table S7). In the majority of Sordariomycetes, two other genes are also located in the *MAT1-1* locus: the *MAT1-1-2* gene encoding a protein with a PPF domain harboring the three invariant residues proline (P), proline and phenylalanine (F), and the *MAT1-1-3* gene encoding a protein with a high-mobility-group (HMG) domain as a DNA-binding motif [55], [59]. Homologs of these two *MAT1-1*-specific mating type genes could not be identified in the genome of *P. confluens*.

A BLAST search with MAT1-2-1 HMG domain mating type proteins identified the ORF *PCON_08389* (scaffold 381) encoding a HMG domain protein as a putative *MAT1-2-1* homolog. The encoded protein displayed the highest degree of identity to the mating type protein MAT1-2-1 of *Gibberella indica* (Table S7).

**Figure 5:**
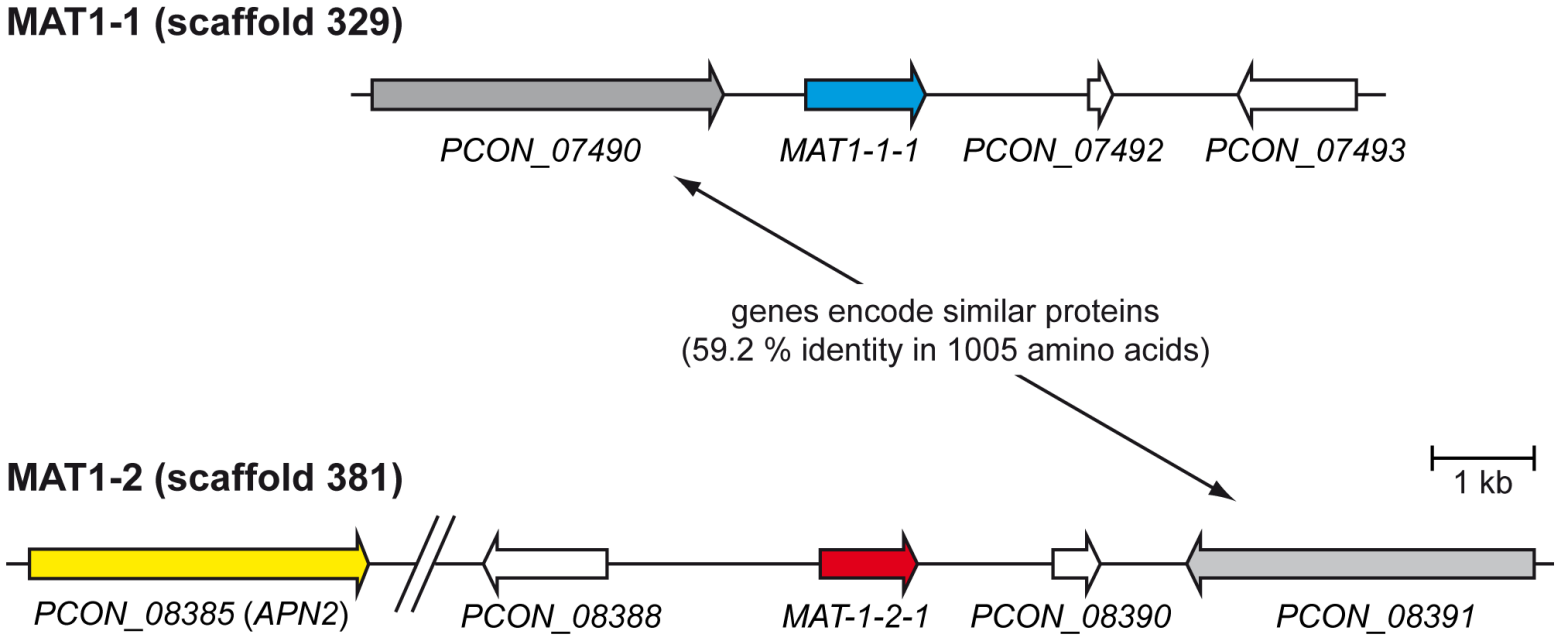
Organization of the mating type loci. The predicted mating type genes *MAT-1-1-1* and *MAT-1-2-1* on scaffolds 329 and 381, respectively, are shown together with adjacent genes. The gap in scaffold 381 was introduced for better visibility and contains the predicted genes *PCON_08386* and *PCON_08387*.

The genes *APN2*, encoding a putative DNA lyase, and *SLA2*, encoding a cytoskeleton assembly control factor, have been reported to be adjacent to *MAT* loci in many filamentous ascomycetes [55], [59]–[62]. The *MAT1-1* locus of *P. confluens* is flanked by genes encoding proteins of unknown function (Figure 5, Table S7). A homolog of *APN2* (*PCON_08385*) is located 10 kb upstream of the *MAT1-2* locus. A *SLA2* homolog (*PCON_02178*) is also present in the *P. confluens* genome but neither on scaffold 329 (*MAT1-1*) nor on scaffold 381 (*MAT1-2*). Recently the genes flanking the mating type locus of the Pezizomycete *T. melanosporum* have been identified as *GSTUMT00001088001* and *GSTUMT00001092001* at the left flank and at the right flank, respectively [58]. Only a homolog of *GSTUMT00001088001* is conserved in *P. confluens* (*PCON_01243*, scaffold 1068) but is not located adjacent to the mating type genes. Interestingly, *PCON_08391* at the right flank of *MAT1-2* and *PCON_07490* at the left flank of *MAT1-1* encode proteins with a high degree of similarity (59.2% identity in 1005 amino acids overlap), and for both proteins the closest homolog in *T. melanosporum* is *GSTUMT0008232001* (Figure 5, Table S7). Aspergilli also contain only one copy of this gene. Therefore, one might hypothesize that this gene was duplicated in *P. confluens* during a recombination event that led to the presence of both mating type loci in one genome and therefore to homothallism. Indeed the phylogenomic tree in phylomeDB shows that *PCON_08391* and *PCON_07490* are species-specific paralogs.

In summary, the genome of the homothallic *P. confluens* has two putative *MAT* loci, typical for homothallic filamentous ascomycetes. The *MAT1-1* and *MAT1-2* loci encode an alpha domain and an HMG domain transcription factor, respectively. The mating type loci are not fused and not in close proximity, similar to the situation in several Eurotiomycetes [63]. Only the *MAT1-2* locus is flanked by the conserved *APN2* gene, whereas both *MAT* loci are flanked by a pair of paralogous genes not found in this location in other ascomycetes (Figure 5). In this respect, *P. confluens* is more similar to derived filamentous ascomycetes than to its closest sequenced relative, *T. melanosporum*, where the *MAT* loci are not flanked by either *APN2* or *SLA2*. Thus, the *P. confluens MAT* loci might reflect a putative intermediate state between the relatively conserved genomic arrangement of mating type loci found in higher filamentous ascomycetes and the specific arrangements found in *T. melanosporum*.

We also searched for homologs to other proteins involved in sexual development and signaling, e.g. pheromone and pheromone receptor genes as well as genes involved in pheromone processing and downstream signaling. With the exception of pheromone genes, which are weakly conserved in ascomycetes, conserved genes for sexual development were found in *P. confluens*, too (for details see Text S2 and Table S7).

### Many Orphan Genes are Upregulated during Sexual Development

A quantitative analysis of gene expression across the *P. confluens* genome was performed based on RNA-seq (Table S2). Of the 13,369 annotated genes, only 58 were not expressed (i.e. have no RNA-seq reads mapped to their exon sequences) in any of the conditions tested. Our analysis was focused on development-dependent gene expression with one tested condition allowing sexual development (sex), and two conditions that only allow the formation of vegetative mycelium (DD and vegmix). Genes that are regulated mostly by developmental factors should be differentially expressed in the comparisons sex/DD and sex/vegmix, but not DD/vegmix. Of the predicted 13,369 protein-coding genes, 5,565 (41%) are regulated differentially in at least one of the three comparisons with thresholds >2 or <0.5, and 3,616 genes (27%) are differentially regulated in at least one comparison with thresholds >4 and <0.25. With the less stringent thresholds, 506 (4%) genes are downregulated and 1,804 (13%) genes are upregulated in both sex/DD and sex/vegmix, but not differentially regulated in DD/vegmix; with the more stringent thresholds, the numbers are 229 (2%) and 1,460 (11%) genes.

**Figure 6:**
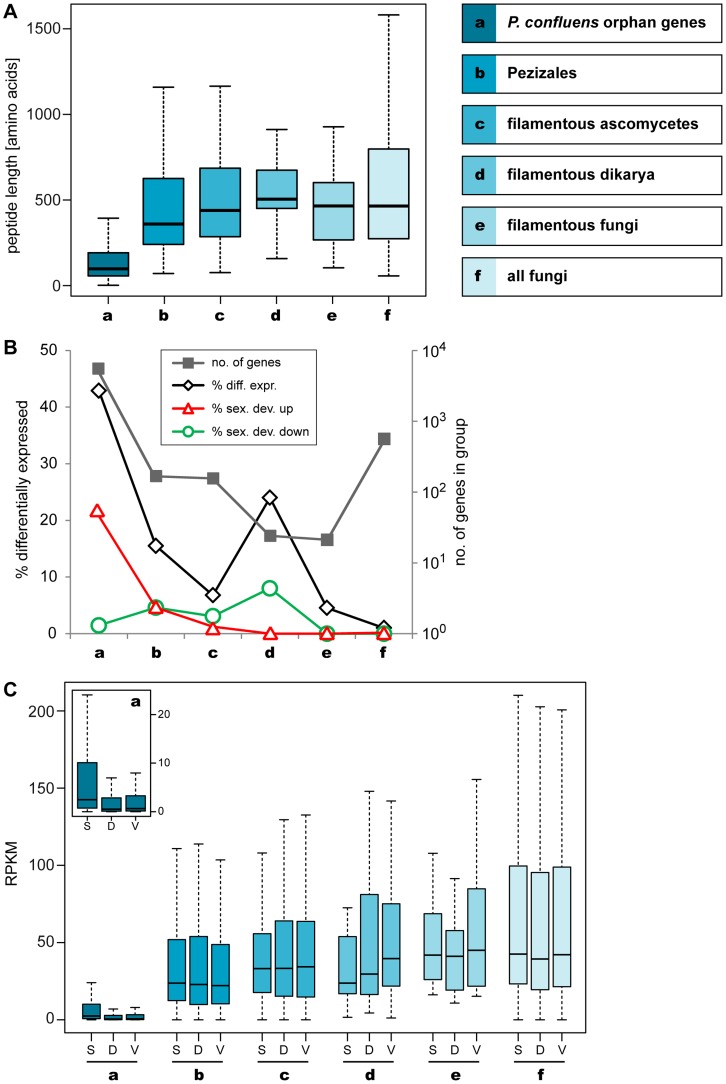
Lineage-specific peptide lengths and gene expression. Peptide lengths and expression of *P. confluens* genes from groups of genes with different levels of evolutionary conservation as indicated. **A.** Boxplot showing the distribution of peptide lengths (outliers left out for better visibility) with the median value as a horizontal line in the box between the first and third quartiles. Peptide length of predicted *P. confluens* orphan genes are smaller than those for the other groups, and from groups a to d, median peptide lengths increase with increasing conservation of genes. **B.** The number of genes in each group are indicated on the logarithmic y-axis to the right, and percentage of genes that are differentially regulated under any condition, and up- or down-regulated during sexual development (up or down in sex/DD and sex/vegmix, data shown for stringent expression analysis) are indicated on the y-axis to the left. The orphan genes have the highest percentage of differentially expressed genes, most of which are upregulated during sexual development. In the other groups, the portion of differentially regulated genes is smaller, and the percentage of genes upregulated during sexual development is similar or smaller than that of downregulated genes. **C.** Overall expression levels given in RPKM (reads per kilobase per million counted reads). For each group, RPKM values were calculated for samples sexual development (S), DD (D), and vegmix (V) as mean RPKM values of the two independent experiments. The boxplot shows the distribution with the median value as a horizontal line in the box between the first and third quartiles (outliers left out for better visibility). The small inlet shows a magnification of the RPKM values for group a (orphan genes). In this group, overall expression is lower, but the distribution of RPKM values for S is significantly different from D and V samples (p<0.01 in Kolmogorov-Smirnov-test). Within the other groups, no significantly different distribution between S, V, and D can be observed.

Studies in animals and plants have shown that genes associated with sexual reproduction evolve more rapidly than genes with other functions [64], [65]. This can be observed not only for single genes, but across genes that are transcriptionally expressed in organs involved in sexual reproduction [66]. In fungi, few studies have addressed this question so far. A genome comparison of *Candida* and related yeast species showed that meiotic genes undergo rapid evolution [67], and similar findings were made in studies of mating type and pheromone signaling genes in filamentous ascomycetes [68], [69]. A recent EST analysis of *Neurospora intermedia* and comparison with other Neurospora species indicated that sex-associated genes, i.e. those genes that are preferentially expressed during sexual development, are rapidly evolving in fungi, too [70]. Here, we approached this question from a different angle by analyzing gene expression levels for *P. confluens* genes with different degrees of evolutionary conservation to find out if genes with different lineage-specificities are preferentially expressed under any of the conditions that we investigated (Figure 6, Table S8). First, we extended our orthology analysis as described in the previous section to include the predicted proteomes of 14 fungi from the major fungal groups (chytrids, zygomycetes, ascomycetes, basidiomycetes), adding two ascomycetous yeasts and an additional chytrid to the previous dataset (Table S8). For downstream analysis, only genes without hits (orphan genes) or with clear reciprocal BLAST hits (orthologs) were used. Genes that are members of gene families with more than one paralog where clear orthologs could not be determined in the analyzed genomes were excluded from the analysis. This left 6,706 *P. confluens* genes in the final analysis that were sorted in six lineage-specific groups (a–f) ranging from *P. confluens* orphan genes (the largest group with 5,737 genes) to genes that are conserved in all analyzed fungal genomes (Figure 6, Table S8). For these genes, we analyzed derived peptide lengths, and expression in the three conditions sex, DD, and vegmix.

Median peptide lengths were shortest in the orphan genes (Figure 6A). This is consistent with observations from a broad range of organisms where conserved proteins are on average longer than poorly conserved proteins [71]. However, at least some of the short peptides may be artifacts of annotation problems, because no homology-based information to aid annotation was available for these genes. But while some of these genes might be due to spurious annotation, more than 40% of the orphan genes are differentially expressed in at least one condition, which might indicate some functionality (Figure 6B). Interestingly, more than 20% of the orphan genes are upregulated during sexual development (in sex/DD and sex/vegmix), while less than 2% are downregulated. This percentage of differentially expressed genes as well as genes upregulated during sexual development is much higher than in all the other groups where less than 5% of genes are upregulated during sexual development. Furthermore, the percentage of up- and downregulated genes is not much different in the other groups.

These trends were also observed when this analysis was performed with lineage-specificity groups obtained from phylogenomics analysis (Figure S6, Tables S8 and S9). This analysis allowed the differentiation between *P. confluens* orphan genes, Pezizales-specific genes, and genes that are specific to Pezizales and Orbiliales; and an increase in peptide lengths as well as a decrease in the percentage of genes that are upregulated during sexual development is correlated with decreasing lineage specificity (Figure S6).

The expression trends can also be seen when analyzing overall expression levels as measured by RPKM (reads per kilobase per million counted reads) values (Figure 6C, Figure S6C). Overall median expression is significantly lower for orphan genes than for the other groups, with a strong increase in Pezizales-specific genes and further slight increase in genes specific to filamentous ascomycetes. A general trend for more conserved genes to have higher expression has also been observed in other organisms [72], [73]. When looking at RPKM values in the three conditions tested, there are no significant differences between conditions within the lineage-specific groups with the exception of the orphan genes. In this group, the median expression is significantly higher in the sexual development condition. Thus, while overall expression is lower for orphan genes, this group comprises much more genes with specific expression during sexual development than the more conserved groups.

There are several hypotheses to explain this finding. One would be the above-mentioned rapid evolution of sex-associated genes leading to apparent orphan genes in its most extreme form. This should be observed especially in species where no sequence information is available for close relatives, as is the case with *P. confluens*. Increased evolutionary rates have generally been observed in genes with higher lineage-specificity, independent of putative function, in an analysis of seven ascomycete genomes [74]. However, there are other mechanisms that may lead to the presence of orphan genes. One is gene loss in all but one (observed) species, although this is unlikely to occur on a larger scale, i.e. for thousands of genes in a single species. Another is the *de novo* gene birth from previously non-coding sequences, a process that in recent years was acknowledged as probably being more common than previously thought [75], [76]. One might speculate that the high number of sex-associated orphan or less conserved genes in *P. confluens* indicates that sexual development allows the “testing” of novel gene-inventions. This might be feasible in filamentous fungi where sexual development is usually not the only means of propagation, and therefore novel genes that are deleterious for sexual reproduction under some circumstances might be retained by purely vegetative propagation until more compatible conditions occur. Another reason could be a more general trend for less conserved genes to be involved in group- or species-specific processes as was found in an analysis of gene expression during different stages of vegetative growth and conidiation in *N. crassa* [77]. Further analyses of more species and transcriptomes from different conditions will be necessary to address these questions.

### Light-Dependent Development and Expression of Photoreceptor Genes

**Figure 7:**
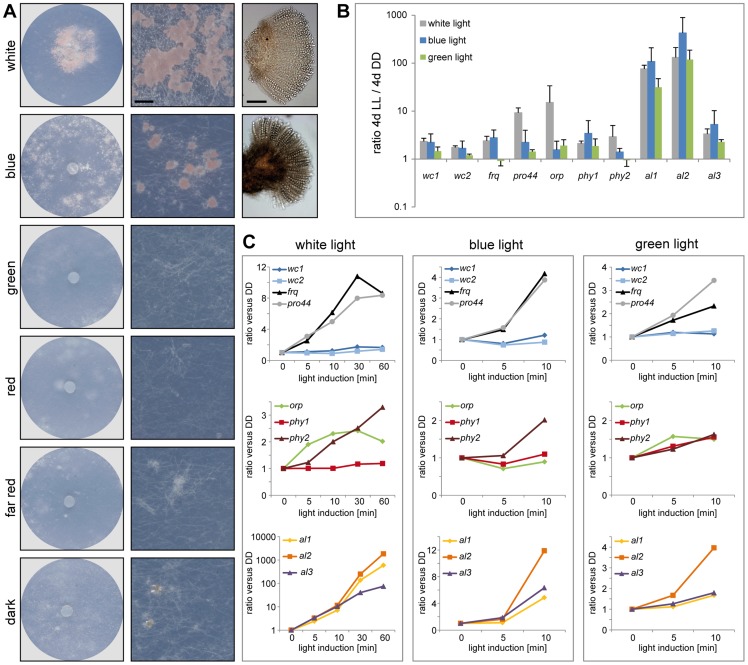
Fruiting body development and gene expression under different light regimes. **A.** Fruiting body development in *P. confluens* is blue light-dependent. *P. confluens* was grown on minimal medium in constant light of different wavelengths as indicated (for filter characteristics see Figure S8). The left column shows petri dishes, the middle column sections from petri dishes (bar 500 µm), and the right column shows mature apothecia in those cases where fruiting bodies are formed (bar 100 µm). Blue light is the effective part of the visible spectrum and induces apothecia formation, similar to white light. Under other light conditions or in darkness, sometimes mycelial aggreagates are formed that can be darkly pigmented, but do not contain sexual structures (e.g. visible in section from petri dish in the dark). **B and C.** Expression of homologs of genes that are involved in blue light responses (*wc1*, *wc2*, *frq*), in light responses and/or fruiting body formation (*pro44*), are photoreceptors for other wavelengths (*orp1*, *phy1*, *phy2*) or carotenoid biosynthesis genes (*al1*, *al2*, *al3*) in other fungi (Table 2). Transcript levels after 4 d of illumination with white, blue, or green light compared to 4 d in darkness (DD) are shown in B, short term light induction (5–60 min after growth in darkness for 4 d) in C. *P. confluens* was grown in minimal liquid medium and harvested under far-red light. Transcript levels were determined by qRT-PCR from at least two independent biological replicates; ratios versus DD samples are shown, error bars in B indicate standard deviation, for standard deviations for short term light induction in C, see Table S10.

It has been shown in many fungi that light can cause developmental changes. In several ascomycetes, illumination promotes vegetative reproduction via conidiospores, while sexual reproduction is observed in darkness [78]. In contrast to this, it was noted already at the beginning of the last century that the formation of apothecia in *P. confluens* is light-dependent [13], [21], and this was confirmed by our studies. Both constant illumination (LL) as well as a 12 h photoperiod promote fruiting body formation, whereas in constant darkness (DD), *P. confluens* is sterile (Figure S7, Figure 7). This complete light-dependency of fruiting body development is uncommon in ascomycetes. It was discussed that perithecia formation in *Trichoderma reseei* might be activated by light [79], but it was shown later on that sexual development in this fungus can also be observed in darkness [80]. However, light-dependent fruiting body development was observed in several basidiomycetes, e.g. *Schizophyllum commune* and *Coprinopsis cinerea*, and in both species, blue light constitutes the effective part of the visible spectrum [81]–[83]. We found that this is also the case for *P. confluens* where blue (400–500 nm) but not green or red light allows fruiting body formation (Figure 7A). This confirms an early study from the 1920s that found wavelengths of 400–550 nm to promote sexual development in this fungus [21].

Despite the fact that no phenotypic responses to wavelengths other than blue have been observed in *P. confluens* yet, its genome encodes putative photoreceptors that cover a range of wavelengths (Table 2), and most of these genes are expressed (Figure 7, Table S10). This includes homologs of *N. crassa* WC-1 and WC-2 (PCON_03119 and PCON_05086), transcription factor/photoreceptor proteins that are part of the white collar complex and mediate all blue-light responses [84]–[88]; however, no homolog was found for the VVD protein that functions in light adaptation in *N. crassa* and *T. reseei* [80], [89], [90]. Two putative phytochromes (PCON_06747 and PCON_08526) are present in the *P. confluens* genome, the first of which is orthologous to FphA that was shown to be the photoreceptor mediating repression of sexual development by red light in *A. nidulans* [91]. *P. confluens* also encodes an ortholog of the *N. crassa* opsin-related protein (PCON_01637) that lacks a conserved lysine residue for chromophore binding, but has no homolog of the rhodopsin NOP-1, a putative green-light receptor [92]–[94] (Table 2). BLAST searches in the *T. melanosporum* genome also failed to identify a NOP-1 homolog, suggesting that the Pezizales might lack a gene for this type of photoreceptor. A gene encoding a putative cryptochrome is present in *P. confluens* (PCON_04132, Table 2), but was the only putative photoreceptor gene for which no expression could be detected under the conditions tested. All other putative photoreceptor genes are expressed, and induced by white light in long-term illumination experiments (4 d LL versus 4 d DD, Figure 7B), but only moderately or not induced by short-term light pulses (from 5 to 60 min, Figure 7C). Interestingly, we also observed some induction with green light for several of the genes, which is surprising because *P. confluens* lacks a rhodopsin-type receptor that was hypothesized to mediate green-light responses in fungi [92]. However, the green-light filter we used has a slight transmission wavelength overlap with the blue light filter (Figure S8), therefore at least part of the green-light responses might be mediated by residual blue light. Nevertheless, *P. confluens* seems to have some green-light sensitivity, because the *orp* gene is light-induced in the short- and long-term illumination experiments, and this effect is stronger with green light than with blue light (Figure 7, B and C).

**Table 2:** *P. confluens* homologs of photoreceptor genes, genes involved in light signaling or light-regulated carotenoid biosynthesis genes in other fungi.

category	*P. confluens* locus tag	*S. macrospora* locus tag	*N. crassa* locus tag	protein^1^	description
photoreceptors	PCON_03119	SMAC_03527	NCU02356	WC-1	*white collar-1*, blue light receptor, transcription factor
	PCON_05086	SMAC_00185	NCU00902	WC-2	*white collar-2*, transcription factor, component of white collar complex
	PCON_06747	SMAC_03470	NCU04834	PHY-1/FphA	phytochrome, red/far-red sensing
	PCON_08526	SMAC_07655	NCU05790	PHY-2	phytochrome, red/far-red sensing
	PCON_04123	SMAC_01274	NCU00582	CRY	cryptochrome, blue-light sensing
	PCON_01637	SMAC_02424	NCU01735	ORP	opsin-related protein
	–	SMAC_06025	NCU10055	NOP-1	homolog of bacteriorhodopsin
	–	SMAC_06136	NCU03967	VVD	*vivid*, PAS/LOV domain, blue-light receptor, photoadaptation
light signal transduction/regulation	PCON_09365	SMAC_03705	NCU02265	FRQ	*frequency*, circadian rhythmicity, light signal transduction
	PCON_03414	SMAC_02423	NCU01731	VeA	*velvet*, red- and blue-light signal transduction
	PCON_06721	SMAC_03223	NCU01154	SUB-1/NsdD/PRO44	GATA-type transcription factor, mediates late light responses in *N. crassa*
carotenoid biosynthesis genes	PCON_03421	SMAC_01244	NCU00552	AL-1	phytoene dehydrogenase
	PCON_03423	SMAC_01277	NCU00585	AL-2	geranylgeranyl-diphosphate geranylgeranyltransferase
	PCON_05718	SMAC_06570	NCU01427	AL-3	farnesyltranstransferase

1 Protein designations are given for *N. crassa*; in cases where the protein was characterized under a different name, names are also given for *A. nidulans* (FphA, NsdD) and *S. macrospora* (PRO44).

We also looked for homologs to genes that act downstream of photoreceptors in light signal transduction in other fungi. Interestingly, *P. confluens* contains a *frequency* (*frq*) homolog (*PCON_09365*), and thus is the most distant relative of *N. crassa* in which this gene is found. The *frq* gene encodes the main regulator of circadian rhythmicity in *N. crassa*, and is a direct target of the white collar complex, but so far *frq* homologs were only found in Sordariomycetes, Dothideomycetes, and Leotiomycetes [95], [96]. The identification of a *frq* homolog in *P. confluens* suggests that *frq* was present in the ancestor of filamentous ascomycetes and was lost several times during evolution, because the *T. melanosporum* genome does not contain a *frq* homolog (data not shown), and no homolog has been detected in the Eurotiomycetes [95], [96]. Similar to *N. crassa* [84], [97], the *P. confluens frq* is strongly induced by short light pulses, and this reaction is mainly mediated by blue light (Figure 7C). *frq* was also upregulated in the long-term illumination experiments (Figure 7B).

In *N. crassa*, an antisense transcript of *frq* is also upregulated by light, and is involved in light-dependent resetting of the circadian clock [98]. Analysis of antisense splice sites did not show any NATs for the *P. confluens frq*, although we cannot exclude the possibility of non-spliced antisense transcripts with this analysis. However, a splice site analysis of RNA-seq reads for *frq* indicated that there might be an alternatively spliced intron in the sense direction overlapping the predicted start codon of the open reading frame (Figure S9). In *N. crassa*, an intron overlapping the *frq* start codon is alternatively spliced resulting in two different forms of the FRQ protein [99], [100]. To determine whether a similar mechanism might occur in *P. confluens*, we performed RT-PCR analysis of a region covering the predicted alternatively spliced intron. Interestingly, there are indeed two different transcript, and alternative splicing of the intron is light-dependent with an increased ratio of spliced versus non-spliced transcript in the light (Figure S9). In the *N. crassa frq*, alternative splicing of the AUG-covering intron is temperature-dependent [99], [100], therefore it seems that similar principles might be at work in these two distantly related fungi, but with different input signals.

We also identified an ortholog for the GATA-type transcription factor NsdD/SUB-1/PRO44 (PCON_06721) that was shown to mediate late light responses in *N. crassa* [101] and is essential for sexual development in *A. nidulans*, *Aspergillus fumigatus*, *N. crassa* and *S. macrospora* [102]–[105]. The developmental function of this protein appears to be conserved (see later section), and expression analyses indicate that light responses, and therefore regulatory activities, might also be similar to those in higher fungi, because the *P. confluens pro44* is strongly light induced after long- and short-term illumination (Figure 7).

We also searched for homologs to carotenoid biosynthesis genes that are known to be light-induced in other fungi [78], [106], [107]. *P. confluens* encodes homologs to the three enzymes AL-1, AL-2, and AL-3 from *N. crassa* [108]–[111], and the corresponding *al* genes (*PCON_03421*, *PCON_03432*, and *PCON_05718*) are strongly light-induced under short- and long-term illumination (Figure 7B, C). Under long-term illumination, blue light has an even stronger effect than white light on *al* gene expression. It has been shown in a previous biochemical analysis that the orange-pinkish pigments that characterize *P. confluens* cultures grown in white or blue light (Figure 7A) are carotenoids [112]. Thus, assuming a function of the *al* genes in *P. confluens* similar to that in *N. crassa*, it seems likely that these carotenoids are synthesized by the products of the *al* genes. Analysis of cultures grown on complete medium that does not support fruiting body formation even in the light indicates that pigment synthesis is independent of fruiting body formation, because light-grown cultures are pigmented even in the absence of apothecia formation (Figure S7).

In summary, our data suggest that light-sensing and signal transduction in *P. confluens* might be comparable to mechanisms in the distantly related species *N. crassa*, and might thus be conserved to a large degree in filamentous ascomycetes. However, output from the light-signaling pathways might be somewhat different, because fruiting body development is strictly light-dependent in *P. confluens*, but not in most other ascomycetes. Blue light has the strongest effect on both morphological as well as gene expression phenotypes, but our data hint at sensitivity to other wavelengths, too, especially in the green part of the visible spectrum. Similar findings were made in *S. macrospora*, where phenotypic changes were observed in response to green light, and in *N. crassa*, where gene expression was found to be modified in mutants of the putative green-light receptor NOP-1 [22], [113].

### Gene Family Expansions and Contractions in *P. confluens*

**Figure 8:**
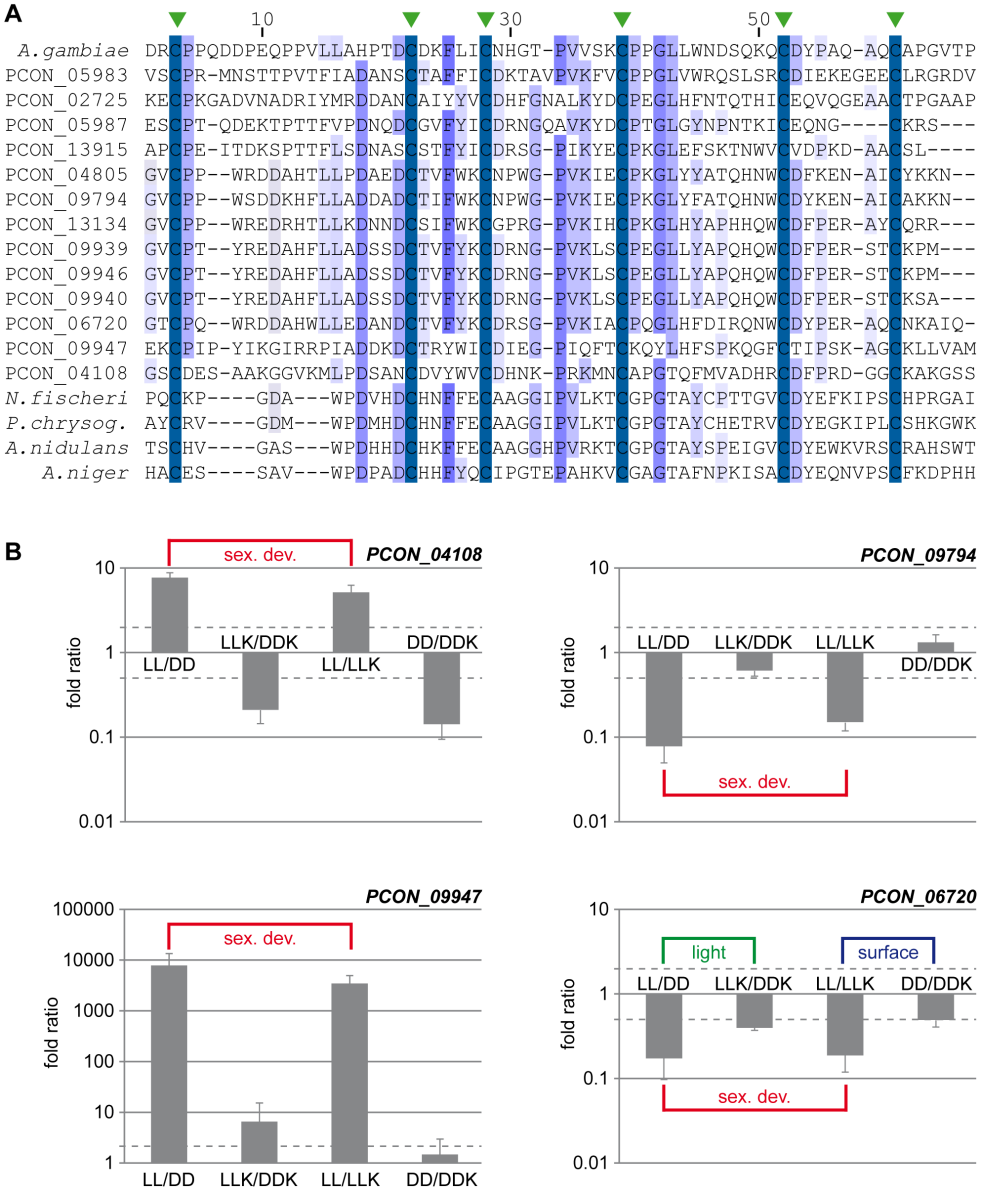
The CBM_14 domain protein family is expanded in *P. confluens*. **A.** Multiple alignment of the CBM_14 domains of proteins from *P. confluens*, several Eurotiales and an insect. In addition to the *P. confluens* proteins, the following proteins were used for Clustal X analysis: *A. gambiae*, *Anopheles gambiae* sp|O76217.2; *A. nidulans*, *Aspergillus nidulans* ANID_00499 from the *A. nidulans* genome project http://www.broadinstitute.org/annotation/genome/aspergillus_group/MultiHome.html; *A. niger*, *Aspergillus niger* ref|XP_001397263.1; *N. fischeri*, *Neosartorya fischeri* ref|XP_001266454.1; *P. chrysog.*, *Penicillium chrysogenum* ref|XP_002561001.1. The conserved cysteine residues are indicated by green arrowheads above the sequence. **B.** Quantitative real time PCR analysis of selected CBM_14 domain-encoding genes from *P. confluens*. Expression was analyzed for each gene in two independent biological replicates for the four conditions LL, DD, LLK and DDK (light and darkness in surface culture and submerged culture). Sexual development is only possible in condition LL. The graph shows mean and standard deviation (for better visualization, standard deviations for negative expression ratios are shown in the negative instead of the positive direction). Expression ratios were calculated to address the question if a gene is differentially regulated during sexual development (i.e. in LL/DD and in LL/LLK), or regulated by light (i.e. in LL/DD and LLK/DDK) or regulated by surface versus submerged growth (i.e. LL/LLK and DD/DDK).

We searched for conserved protein domains in the predicted proteins from *P. confluens* and seven other filamentous fungi to identify protein families that are expanded in *P. confluens* (Table S11). Among the expanded gene families are two that encode mostly small, extracellular proteins, namely CBM_14 and Defensin_2 domain proteins. The CBM_14 domain (Chitin binding Peritrophin-A domain) is mainly found in metazoa, and in fungi so far has been described only in the Avr4 protein from *Cladosporium fulvum* and, with one gene per genome, in the genomic sequences from several Aspergilli [114], [115]. BLASTP analysis in GenBank revealed that there are also some predicted CBM_14 proteins in other Eurotiales (Figure 8A), but not in other fungal groups. In contrast, there are 13 proteins with CBM_14 domain in *P. confluens* (Figure 8A). All of these are predicted as extracellular and have a putative cleavable N-terminal signal peptide for co-translational insertion into the ER (data not shown). They are mostly 80–140 amino acids long with the exception of PCON_04108 (351 amino acids), and contain no other recognized domains besides CBM_14. Some of the corresponding genes are clustered within the same genomic region, indicating that the genes might have arisen through duplications at certain gene loci: *PCON_09939*, *PCON_09940*, *PCON_09946*, and *PCON_09947* lie within 20 kb of scaffold 486, and *PCON_05983* and *PCON_05987* lie within 9 kb on scaffold 228.

The RNA-seq data show that more than half of the CBM_14 domain proteins are upregulated during sexual development. Furthermore, the overall expression levels of these proteins vary greatly ranging from no sequence reads in certain conditions to >13,000 reads (normalized to kb of mRNA, Figure S10). For example, the clustered genes *PCON_09939* to *PCON_09947* are preferentially or only expressed during sexual development, while others are more strongly expressed under non-sexual conditions. To address the expression of some CBM_14 domain genes in more detail and distinguish between regulation by sexual development, light, and growth conditions (surface versus submerged), we performed qRT-PCR for four genes under the conditions LL, DD, LLK and DDK (light and darkness in surface culture and submerged culture, Figure 8B). These combinations distinguish if a gene is differentially regulated during sexual development (i.e. in LL/DD and in LL/LLK), or regulated by light (i.e. in LL/DD and LLK/DDK) or regulated by surface versus submerged growth (i.e. in LL/LLK and DD/DDK). *PCON_04108* and *PCON_09947* are upregulated during sexual development, but not consistently regulated by light or surface culture (*PCON_09947* is slightly upregulated by light, but the extent of regulation is far lower than the development-dependent regulation). *PCON_09794* is downregulated during sexual development, but not regulated by the other two stimuli, whereas *PCON_06720* is downregulated during sexual development and (to a lesser extent) downregulated in the light and in surface cultures. Overall, the four genes have distinct expression patterns, which might indicate that they are functional in *P. confluens*. The Avr4 protein from the phytopathogenic *C. fulvum* was shown to bind chitin and protect it from hydrolysis by plant chitinases [115]. *P. confluens* is non-pathogenic, but one might speculate that secreted CBM_14 domain proteins might protect the fungus from microbial attacks in its soil habitat.

A second expanded gene family encoding small, secreted proteins is the Defensin_2 family (Table S11). Defensin_2 domain proteins are mostly known from arthropods where they are part of the immune system and act against bacteria [116], [117]. In fungi, only one Defensin_2 domain protein has been described in detail, namely Plectasin from *Pseudoplectania nigrella*, a member of the Pezizales [118], [119]. In *P. confluens*, the family comprises six members (PCON_01606 to PCON_01611), all of which are between 92–96 amino acids long, including predicted signal peptides of 15–23 amino acids, and are encoded by a cluster of genes within 10 kb on scaffold 1117 (Figure S11A and B). Two of the genes (*PCON_01607* and *PCON_01611*) are pseudogenes, and interestingly the expression of the two pseudogenes is much lower than that of the other four genes under all conditions investigated by RNA-seq (Figure S11C). Both pseudogenes have one of the functional Defensin genes as closest homolog (Figure S11D), and one might speculate that they have arisen from gene duplications within the Defensin gene cluster, but lost (most of) their expression and function which was retained by their closest homologs. Interestingly, all genes including the pseudogenes are downregulated during sexual development (Figure S11C). However, a comparison of intergenic regions showed that these are not conserved, in contrast to the coding regions. On the one hand, this might indicate that the regulatory sequences responsible for development-specific regulation are too small or non-conserved to be detected in these comparisons. Another explanation could be that regulation is achieved through chromatin organization of the complete gene cluster, similar to what was described for secondary metabolism gene clusters in fungi [120], [121].

In addition to the Defensin_2 domain proteins in *P. confluens* and *P. nigrella*, searches in other sequenced fungal genomes identified Defensin_2 domain proteins only in Eurotiomycetes (Figure S8D). This phylogenetic distribution is similar to that of the CBM_14 domain proteins. One might speculate that both classes of small, secreted proteins arose from horizontal transfer events from insects into fungi; alternatively, this could be a case of gene loss or rapid evolution in the other ascomycete groups. Horizontal gene transfer has been acknowledged as an important mechanism in fungal evolution only in recent years, and the availability of genome sequences has made in-depth analyses possible [122]. A transfer event of carotenoid biosynthesis genes from fungi into insects has been shown already [123], therefore it seems possible that a transfer in the reverse direction might also occur. Interestingly, both the Defensin_2 as well as the CBM_14 domain proteins might be involved in defense mechanisms against microorganisms in arthropods. One might hypothesize that the corresponding genes were acquired by fungi through horizontal gene transfer, and were retained because they offer a selective advantage in the microorganism-rich soil habitat.

A third gene family that is expanded in *P. confluens* compared to *T. melanosporum* (but not to other ascomycetes) comprises genes with HET (heterokaryon incompatibility protein) domains. While there are only two HET-domain containing protein in *T. melanosporum* [124], there are 15 in *P. confluens* (Table S11). However, there are 11–101 predicted HET domain proteins in various species of higher filamentous ascomycetes, therefore the number in *T. melanosporum* might have been reduced by selective gene loss. In the Sordariomycetes *N. crassa* and *Podospora anserina*, HET domain proteins were shown to mediate heterokaryon incompatibility (HI) [125], [126]. Homologs to many known HI proteins can be found in *P. confluens* (Table S12); however, none of the *P. confluens* HET-domain proteins contains additional WD repeat, NACHT, leucine- or glycine-rich repeat domains that are found in the HET domain in HI proteins from *N. crassa* and *P. anserina* [127]. Therefore, if HI is present in *P. confluens*, it is unlikely to be mediated by the same proteins that regulate HI in Sordariomycetes.

A number of protein families are have fewer members or are missing in *P. confluens* compared to more derived ascomycete groups (Table S11). Most prominent among these are gene families involved in secondary metabolism (see next section), transporter protein families, and several gene families involved in carbohydrate metabolism. The low number of genes for some transporter families might be connected to the limited capabilities for the production (and presumably export) of secondary metabolites; whereas the reduced number of genes for certain enzymes involved carbohydrate metabolism might either be a niche-specific adaptation or indicate that the expansion of carbohydrate-activating enzymes occured only in more derived ascomycete groups. Overall, gene family contraction in *P. melanosporum* is much less prominent than in *T. melanosporum* [9].

### Genes for Secondary Metabolism

In contrast to the gene families described in the previous section, genes encoding enzymes for the biosynthesis of polyketides or non-ribosomal peptides, typical secondary metabolites of filamentous ascomycetes, are underrepresented in the *P. confluens* genome (Figure S12A). There are seven putative non-ribosomal peptide synthase (NRPS) genes, and one polyketide synthase (PKS) gene, much fewer than in the genomes of higher filamentous ascomycetes [128]–[130]. The predicted NRPS protein PCON_02859 has the typical domain structure of siderophore NRPSs and is part of a cluster of genes homologs of which are involved in siderophore biosynthesis in other fungi [131], [132] (Figure S12B). A second putative NRPS gene (*PCON_07777*) is not clustered and does not have homology to NRPSs with known function. The remaining five NRPS genes all have a domain structure that is typical for alpha-aminoadipate reductase (AAR)-type NRPSs (Figure S12A), and (with the exception of PCON_04030) all have high sequence similarity to aminoadipate semialdehyde dehydrogenase, an enzyme of lysine biosynthesis that is conserved in fungi [129]. Most fungi have only one AAR-type NRPS [129], therefore the high number of corresponding genes in *P. confluens* is somewhat unusual. Possible explanations may be selective amplification of this specific gene family or loss of most other NRPS genes with exception of AAA-type NRPS genes. However, at least PCON_04030 might have a function other than lysine biosynthesis, because the gene is located adjacent to the single PKS gene (*PCON_04029*) in a gene cluster that also contains other genes encoding enzymes that might be involved in the biosynthesis of secondary metabolites (Figure S12C). The genes in this cluster might be involved in the production of a hybrid polyketide/non-ribosomal peptide. The existence of gene clusters encoding separate PKS and NRPS proteins that act in a common biosynthetic pathway was demonstrated, for example, in *A. nidulans*, where such a cluster is responsible for the production of Emericellamide [133].

The single predicted PKS PCON_04029 is a type I PKS. In filamentous ascomycetes, there is usually one type III PKS encoded in the genome [130], but type III PKSs are missing in *P. confluens*. A low number of PKS and NRPS genes was also found in *T. melanosporum*, and therefore seems to be typical for lower filamentous ascomycetes rather than a result of the truffle-specific life-style [9]. In summary, our analysis shows that the presence and clustering of NRPS and PKS genes is already established in *P. confluens*. In combination with the fact that no PKS genes and only the single AAR-type NRPS gene were found in Taphrinomycotina, Saccharomycotina, and zygomycete genomes [130], this suggests that the evolution and expansion of PKS and NRPS gene families began in a common ancestor of filamentous ascomycetes, whereas the evolution of type III PKS genes might be a later event that occurred in higher filamentous ascomycetes. However, at present it cannot be excluded that the low number of putative PKS and NRPS genes is an adaptation to specific ecological niches in both *T. melanosporum* and *P. confluens* [134], [135]; more Pezizomycete genome sequences will be needed to resolve this question.

### Genes Encoding Putative Transcription Factors

The number of putative transcription factor genes (excluding general transcription factors that regulate RNA polymerase) in filamentous fungi varies from 182 in *N. crassa* to more than 600 to 800 in several *Fusarium* species [39], [136]–[138]. In truffle, 201 transcription factor genes were predicted [139], while our survey of the *P. confluens* genome indicated 177 putative transcription factor genes (Table 3, Table S13). Similar to other filamentous ascomycetes, the largest group comprises putative Zn_2_-Cys_6_ binuclear cluster (Zn cluster) proteins; thus, the regulatory capacity of *P. confluens* appears to be similar to that of other filamentous fungi. 54 of the putative transcription factor genes are differentially expressed in at least one of the comparisons that were investigated (Figure S13). Eight genes are strongly upregulated during sexual development, and among these is *PCON_02619*, the gene encoding the ortholog of STE12, a transcription factor that was shown to be involved in sexual development in yeast and filamentous ascomycetes [140]–[143]. Expression of *PCON_02619* and five additional transcription factor genes was characterized in more detail by qRT-PCR (Figure S14). Development-dependent expression was confirmed for those genes that were predicted to be differentially regulated during sexual development by the RNA-seq analysis, showing that our sampling strategy is indeed suitable for identifying developmentally regulated genes. The *STE12* ortholog *PCON_02619* was confirmed as one of the most strongly upregulated transcription factors during sexual development. Interestingly, the corresponding *SteA* gene in truffle is downregulated in fruiting bodies [139], suggesting a functional diversification of this conserved transcription factor among Pezizales.

**Table 3:** Overview of putative transcription factor genes.

pfam domain model^1^	no. of genes	diff. expressed	% diff. expr.
bZIP_1	6	0	0.0
bZIP_2	3	0	0.0
bZIP1 and bZIP2	3	1	33.3
CBF	3	0	0.0
CBFB_NFYA	1	0	0.0
CBFD_NFYB_HMF	7	1	14.3
Fork_head	4	1	25.0
GATA	8	3	37.5
HLH	8	4	50.0
HMG_box	8	2	25.0
Homeobox	3	1	33.3
Myb_DNA-binding	13	3	23.1
SRF-TF	2	0	0.0
zf-C2H2	25	10	40.0
zf-C3HC4	20	5	25.0
Zn_clus	63	23	36.5
*sum*	*177*	*54*	*30.5*

1 Searches for conserved protein domains (pfam domains) were performed with the HMMER 3.0 program hmmsearch [160], [161]. Numbers of genes that are differentially expressed in at least one of the comparisons (sex/DD, sex/vegmix, DD/vegmix) are indicated, detailed information for each gene is given in Table S13.

### Comparative Expression Analysis to Identify Genes with Conserved Expression Patterns during Sexual Development

**Figure 9:**
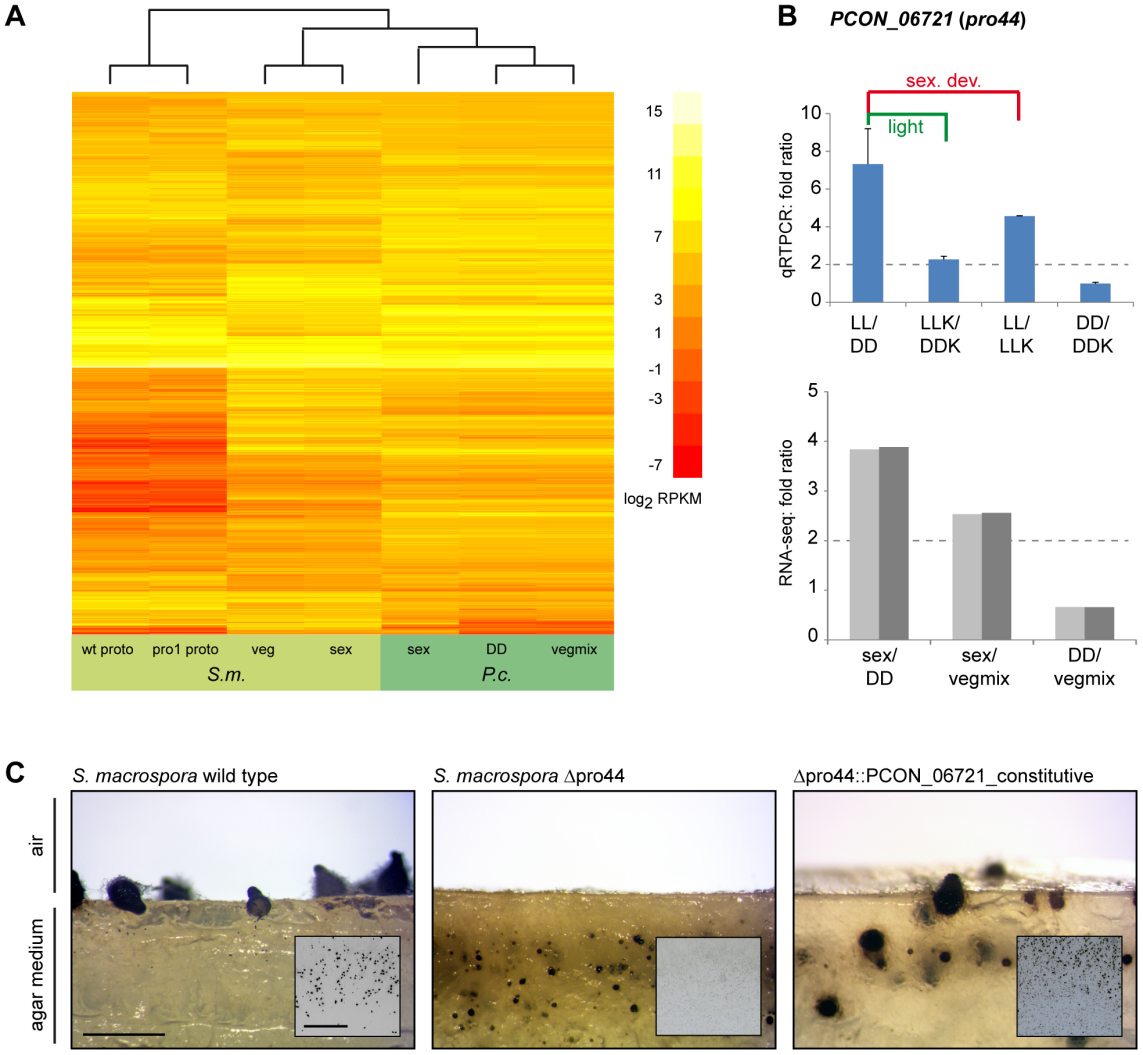
Comparative transcriptome analysis with *S. macrospora*, and functional conservation of the transcription factor gene *PCON_06721*. **A.** Comparative analysis of RPKM values for all orthologous genes for *P. confluens* and *S. macrospora* (with the exception of genes without read counts in one or more conditions). **B.** Quantitative real time PCR analysis of the predicted transcription factor gene *PCON_06721* in *P. confluens*. Expression was analyzed in two independent biological replicates for the four conditions LL, DD, LLK and DDK (light and darkness in surface culture and submerged culture). Sexual development is only possible in condition LL. Expression ratios and standard deviations were calculated to address the question if the gene is differentially regulated during sexual development (i.e. in LL/DD and in LL/LLK), or regulated by light (i.e. in LL/DD and LLK/DDK) or regulated by surface versus submerged growth (i.e. LL/LLK and DD/DDK). *PCON_06721* is upregulated during sexual development, but also slightly upregulated by light. RNA-seq results are given for comparison. **C.** Complementation of the *Sordaria macrospora* mutant Δpro44 with the *P. confluens* ortholog *PCON_06721*. The mutant was transformed with plasmid pFA50 that carries the *PCON_06721* ORF under control of the respective *gpd* and *trpC* promoter and terminator sequences of *Aspergillus nidulans*. The figure shows a side view (longitudinal section) of the region comprising the agar/air interface from cultures of the wild type, the sterile mutant Δpro44 and a complemented transformant (Δpro44::PCON_06721_constitutive). The small inserts show photographs of Petri dish sections. The *S. macrospora* wild type forms mature perithecia at the agar/air interface, whereas the mutant only forms protoperithecia that are submerged in the agar. Complemented transformants produce mature perithecia at the agar/air interface like the wild type. Strains were grown on corn meal agar; photographs were taken after 8 d; scale bar indicates 1 mm, and 1 cm in the inserted photographs.

Comparison of gene expression patterns can serve to identify core genes that are involved in biological processes, because conservation of expression is a strong indicator for functional significance [144], [145]. In previous studies, we have already demonstrated that development-dependent expression of several genes is conserved in *P. confluens* and other, more derived filamentous ascomycetes, and that conservation of gene expression can be used as a criterion to identify genes that play a role during sexual development in fungi [6], [18], [19]. Here, we compared the RNA-seq results from *P. confluens* with published data from different developmental stages of *S. macrospora* [24] (Table S14). A cluster analysis of RPKM values for all orthologous gene pairs showed that overall expression patterns in total vegetative or sexual mycelia from *S. macrospora* are more similar to those from total mycelia from *P. confluens* than to expression patterns from isolated young fruiting bodies (protoperithecia) of *S. macrospora* (Figure 9). Similar results were obtained in a cluster analysis of expression ratios where comparisons of *S. macrospora* protoperithecia with total mycelia cluster separately from other comparisons (Figure S15). This indicates that similar tissues/organs in different species might have more similar expression patterns than different tissues/organs from the same species; in other words, tissue/organ-specific gene expression might be conserved across fungi. While more comparative studies of specific fungal organs or cell types are needed to confirm this, this finding is similar to results from organ-specific gene expression analysis in mammals [66].

Among the genes that were upregulated during sexual development in both species are the predicted transcription factor gene *PCON_06721* and its *S. macrospora* ortholog *pro44*. The *S. macrospora* gene was shown previously to be essential for fruiting body development, similar to the corresponding homologous genes in *A. nidulans*, *N. crassa*, and *A. fumigatus* [102]–[105]. In *N. crassa*, the homologous *sub-1* gene was also shown to be light-regulated at the level of transcription, and to be involved in light-regulation of downstream genes [101]. *PCON_06721* (*P. confluens pro44*) is also light-induced (see section about light-dependent regulation and Figure 7), and further qRT-PCR analysis of *PCON_06721* showed that it is upregulated by both light and sexual development, indicating that *PCON_06721* might be involved in sexual development and development-independent light reactions (Figure 9B). *PCON_06721* and its homologs in other fungi encode GATA-type transcription factors. Whereas the C-terminal GATA domain is highly conserved in all homologous proteins, the N-terminal part of the protein is only weakly conserved (Figure S16). To address the question whether the developmental function of *PCON_06721* might be conserved despite limited sequence conservation, we transformed an *S. macrospora* Δpro44 strain with a construct expressing the *P. confluens PCON_06721* gene (Figure 9C). The *S. macrospora* Δpro44 is sterile and forms protoperithecia, but no mature perithecia. Transformation with *PCON_06721* restored the fertility of the deletion mutant, demonstrating that the gene from the basal filamentous ascomycete *P. confluens* is functional in the more derived Pezizomycete *S. macrospora*. This indicates that this transcription factor gene is one of the core regulators of sexual development across filamentous ascomycetes.

### Conclusions

Here, we have analyzed the genome and development-dependent transcriptomes of *P. confluens*. This is the second Pezizomycete genome to be sequenced, but the first of a Pezizomycete with a more “typical” saprobic lifestyle and apothecium when compared to the more specialized life style of the truffle *T. melanosporum*. Overall genomic synteny with *T. melanosporum* is low, but regions of microsynteny between *P. confluens* and truffle are more numerous than between *P. confluens* and other ascomycetes, indicating that the two Pezizomycetes are more closely related to each other than to other ascomycete groups; however, the level of synteny still suggests a wide evolutionary range within the Pezizomycetes. This is consistent with phylogenetic analyses based on rDNA sequences that placed *Pyronema* and *Tuber* in subgroups C and B, respectively, of the Pezizomycetes [146], [147].

The *P. confluens* genome has a number of characteristics that are similar to higher filamentous ascomycetes, and distinct from *T. melanosporum*, namely its size of 50 Mb, gene content of ∼13,400 protein-coding genes, and low repeat content. However, several typical features of higher filamentous ascomycetes are different in *P. confluens*, allowing conclusions about the evolution of these features in fungi. For example, the mating type genes are conserved, but in contrast to higher filamentous ascomycetes, their genomic environment is not. Also, clustered genes for secondary metabolites exist, but in much lower numbers than in other species. On the other hand, *P. confluens* has a full complement of fungal photoreceptors, and expression studies indicate that light-sensing and signaling might be similar to more derived species and therefore represent basic features of filamentous ascomycetes. Several families encoding predicted small secreted proteins are expanded in *P. confluens* and present in only few other fungal groups, making it possible that they were acquired by horizontal gene transfer. By analyzing spliced RNA-seq reads in antisense direction to annotated genes, we were able to deduce the presence of natural antisense transcripts in *P. confluens*; and this principle might be of interest for non-strand-specific RNA-seq experiments in other organisms.

Interestingly, among the *P. confluens* orphan genes, a disproportionally high number is upregulated during sexual development, consistent with a hypothesis of rapid evolution of sex-associated genes. Comparative transcriptome analysis with *S. macrospora* identified the transcription factor gene *PCON_06721*, the ortholog of *S. macrospora pro44*, as upregulated during sexual development in both species, and complementation of an *S. macrospora* deletion mutant with the *P. confluens* gene demonstrated the conserved function of this regulator of sexual development. In summary, the *P. confluens* genome helps to close a sequence gap at the base of the filamentous ascomycetes, and the genome and transcriptome data are valuable resources for the analysis of fungal evolution and sexual development.

## Materials and Methods

### Strains and Culture Conditions

The sequenced strain is *Pyronema confluens* CBS100304, obtained from the CBS (Centraalbureau voor Schimmelcultures, Utrecht, NL). The strain was grown on minimal medium as previously described [6] or on cornmeal medium [148]. *Sordaria macrospora* strains used in this study are the wild type (FGSC 10222) and a *pro44* deletion mutant from the strain collection of the Department of General and Molecular Botany at the Ruhr-Universität Bochum (Nowrousian, Teichert and Kück, unpublished). *S. macrospora* was grown on cornmeal medium as previously described [148].

### Light Treatment for the Analysis of Light-Dependent Development and Gene Expression

For standard cultures, white light with a spectral range from 400 to 700 nm (Osram L36W/840; 1.57 lx at culture level) was used. For wavelength-dependent development and gene expression analyses, LEE filters (Andover, UK) with different transmission characteristics were used (light intensity at the level of the cultures given in lux, for transmission data see Figure S8): far red (LEE Filter 787 marius red; 0.02 lx), red (LEE Filter 106 primary red; 0.54 lx), green (LEE Filter 139 primary green; 0.60 lx) and blue (LEE Filter 363 medium blue; 0.14 lx). For light induction experiments, samples were harvested under far-red light (Philips PF712E darkroom safe light) after 4 d continuous light (LL), or continuous darkness (DD), or DD and 5–60 min of light induction with the respective wavelengths.

### DNA Preparation and Sequencing

Genomic DNA from *P. confluens* was prepared from mycelium grown for 3 days in minimal medium. Mycelium was frozen in liquid nitrogen, pulverized, and incubated in equal volumes of lysis buffer (0.6 M, 10 mM EDTA, 100 mM Tris-HCl pH 8.0, 1% SDS) and phenol/chloroform (1∶1) at room temperature for 10 min with constant shaking. After centrifugation, the supernatant was again treated with an equal volume phenol/chloroform (1∶1), and this step was repeated until the supernatant was clear. It was then treated with RNase A, and afterwards again with phenol/chloroform. Genomic DNA was precipitated with sodium acetate (pH 7.0) and ethanol. Roche/454 sequencing was performed with 20 µg genomic DNA at Eurofins MWG GmbH (Ebersberg, Germany) on a GS FLX system. Illumina/Solexa paired-end sequencing was performed with 5 µg genomic DNA at GATC Biotech (Konstanz, Germany) on a HiSeq 2000. An overview of obtained sequence reads is given in Table S1.

### Assembly and k-mer Frequency Analysis

The 454 raw data were extracted from sff files and converted to fasta files using sff_extract.py (Jose Blanca and Bastien Chevreux, http://bioinf.comav.upv.es/sff_extract/index.html). 454 and Illumina raw data were trimmed with custom-made Perl scripts to remove reads with undetermined bases (“N”) and for trimming of low quality bases (phred score <10) from the 3′ end as described (available at http://c4-1-8.serverhosting.rub.de/public/software.html) [24], [104]. 454 reads were assembled with the Celera assembler [149]. The trimmed 454 and Illumina reads as well as the 454-based Celera assembly were used for an assembly with Velvet 1.1.04 [150] with the following parameters for velveth (k) and velvetg (all others): k 41, exp_cov 100, cov_cutoff 2, long_mult_cutoff 0, ins_length 300. Overlapping Velvet scaffolds were merged further using CAP3 [151].

The rDNA unit (scaffold 1635) was assembled separately from 454 reads. BLAST searches with rDNAs from *S. macrospora* and several publicly available Pezizales rDNAs against the 454 reads and the Celera assembly were used to obtain sequences with homology to rDNA. Theses reads were assembled with CAP3 [151] to obtain an rDNA unit that contains the 18S, 5.8S, and 28S rRNA genes as well as the internal transcribed spacers 1 and 2.

k-mer frequencies were analyzed based on the Illumina reads with an algorithm described for potato genome [23]. The algorithm was used to write a custom Perl program. Based on the fastq data of the Illumina reads, k-mers of 31 and 41 bases were analyzed.

### RNA Preparation, qRT-PCR, and RNA-seq

For RNA extraction, *P. confluens* mycelia were grown as described in liquid medium (minimal medium or cornmeal medium) either in darkness (and harvested in dark red light) or in light, as surface cultures or submerged (shaking cultures) [6]. For the analysis of effects of different wavelengths on fruiting body formation and gene expression, LEE filters (Andover, UK) were used as described above. RNA was prepared with the RNeasy lipid tissue mini kit (Qiagen, Hilden, Germany) as described [6]. Reverse transcription and qRT-PCR were performed as described previously [18], [152], oligonucleotide primers are given in Table S15.

For RNA-seq analysis, 50 µg total RNA from several growth conditions were pooled to generate the sex, DD, and vegmix samples. RNA for sample sex was extracted from mycelia grown in minimal medium in surface culture in constant light. Only under these conditions is *P. confluens* able to develop fruiting bodies. Equal amounts of RNAs from mycelia grown for 3, 4, and 5 d were pooled to represent a high number of genes that are expressed during fruiting body development. The DD samples comprised RNAs from mycelia grown for 3, 4, and 5 d in minimal medium in submerged culture in constant darkness, which prevents fruiting body formation. The vegmix samples also contained only RNAs from mycelia that could not develop fruiting bodies, but from a mixture of growth conditions different from the DD samples: for the vegmix samples, we used mycelia grown for 3 d in complete medium (cornmeal medium) in surface culture in constant darkness, mycelia grown for 3 d submerged in minimal medium in constant light, and mycelia grown for 3 d submerged in minimal medium in constant darkness. Two independent biological replicates of each condition (sex, DD, and vegmix) were used for sequencing. cDNA and library preparation for RNA-seq as well as Illumina/Solexa paired-end sequencing was performed at GATC Biotech (Konstanz, Germany). Indexed cDNA libraries for multiplexing were prepared with the TrueSeq RNA sample preparation kit (Illumina, San Diego, CA, USA). One library was prepared for each independent replicate for each of the three conditions (sex, DD, and veg), and the resulting six libraries were pooled and sequenced in one lane on a HiSeq 2000. An overview of obtained sequence reads is given in Table S1.

### Annotation and Analysis of Repeat Content

RNA-seq reads were assembled with Trinity [153], and assembled transcripts were mapped to the genome sequence with PASA [154]. The longest full length ORFs identified by PASA were used to train AUGUSTUS and SNAP, then gene models were predicted independently with AUGUSTUS, SNAP, and GeneMark-ES [155]–[158]. The resulting annotation from each of the prediction programs was used together with the RNA-seq data as input to MAKER, a program that integrates the different sources of gene evidence [25]. Detailed parameters that were used for the gene predictions are available at https://github.com/hyphaltip/fungi-gene-prediction-params/. Initial automated gene predictions were checked for consistency (e.g. presence of start/stop codons) and manually curated in about 10% of all cases. UTR predictions were refined/improved based on the RNA-seq data using custom-made Perl scripts as described previously [24]. For each of the predicted proteins, the protein with the highest sequence identity in GenBank (nr) was determined using BLASTP [48], and putative localizations of the predicted proteins were determined with WoLF PSORT [159] (Table S2). Searches for conserved protein domains (Pfam-A domains, http://pfam.sanger.ac.uk/) were performed with the HMMER 3.0 program hmmsearch [160], [161]. A chi-square test to determine which domains are over- or underrepresented in *P. confluens* was performed in R. tRNAs were predicted using a combination of Infernal 1.0, tRNAscan-SE, TFAM 1.0, and ARAGORN [162]–[165].

Analysis of transposable elements and other repeats was performed with RepeatMasker (A.F.A. Smit, R. Hubley, P. Green; www.repeatmasker.org) based the RepbaseUpdate library [166] and a library of *de novo*-identified *P. confluens* repeat consensus sequences that was generated by RepeatModeler (A.F.A. Smit, R. Hubley; www.repeatmasker.org/RepeatModeler.html). First, the *P. confluens* genome sequence was analyzed using the RepBase Update library and species-specification “fungi”. In a second step, repeats were identified *de novo* from the *P. confluens* genome using RepeatModeler, and the RepeatMasker analysis was repeated with the *P. confluens*-specific repeat library generated by RepeatModeler. The results of both RepeatMasker runs were combined using custom-made Perl scripts to remove redundancy and only keep non-overlapping repeat regions. Histograms of percent divergence, percent insertions, and percent deletions compared to the repeat consensus sequences were generated based on the output information from RepeatMasker.

### RIP Index Calculation

Composite RIP indices were calculated with Perl script RIP_index_calculation.pl (https://github.com/hyphaltip/fungaltools/blob/master/scripts/RIP_index_calculation.pl) on DNA sequences of 500 bp in sliding windows (window step size 100 bp) based on a method used in [167]. Briefly, a RIP product index (TpA/ApT) and RIP substrate index (CpA+TpG/ApC+GpT) [168], [169] are calculated. Sequences that have been subjected to RIP have a product index of at least 1.1 and a substrate index of less than 0.9, while sequences that have not been subjected to RIP have a product index of less than 0.8 and substrate index of at least 1.1. The composite RIP index is calculated by subtracting the substrate index from the product index; positive values imply that the DNA has been subjected to RIP [167].

### Analysis of Natural Antisense Transcripts (NATs)

Predicted splice sites from the junctions.bed output of Tophat [31] were analyzed with respect to strand based on the intron consensus sequences (5′ GT or GC, 3′ AG) and overlap with annotated protein-coding genes using custom-made Perl scripts based on BioPerl modules [170]. Splice sites in antisense direction to annotated genes were further filtered to include only sites covered by at least five spliced sequence reads, and with a coverage of more than 10% of the average coverage of the predicted sense-transcript. Remaining putative antisense splice sites were checked manually to remove splice sites that were most likely due to annotation errors or within repeat-rich regions or close to sequence gaps.

### Synteny Analysis

Regions of sequence similarity were determined with the PROmer algorithm from the MUMmer package version 3.23 [47]. The resulting files were used as input to mummerplot, and percent identity plots and dot plots of PROmer results were visualized with gnuplot (www.gnuplot.info) based on the mummerplot output files.

An orthology-based analysis of synteny was performed by determining orthologs for all *P. confluens* genes in the predicted proteomes of ten filamentous fungi by reciprocal BLAST analysis [48], and using custom-made Perl scripts based on BioPerl modules [170] to determine the positions of orthologous proteins on sequenced chromosomes or contigs.

### Phylogenomics Analysis and Estimation of Divergence Times

The predicted proteomes of *P. confluens* and the following 17 other fungal species were used for the reconstruction of the phylome: *Agaricus bisporus* [171], *Arthrobotrys oligospora* [10], *Blumeria graminis* [172], *Coccidioides immitis* [173], *Emericella nidulans* [174], *Gibberella zeae* [138], *Laccaria bicolor* [175], *Mycosphaerella graminicola* [176], *Neosartorya fischeri* [177], *Neurospora crassa* [178], *Phaeosphaeria nodorum* [179], *Saccharomyces cerevisiae* [180], *Schizosaccharomyces pombe* [181], *Sclerotinia sclerotiorum* [182], *Sordaria macrospora* [22], *Tuber melanosporum* [9], *Yarrowia lipolytica* [183]. 13 of the proteomes belonged to other Pezizomycotina species, *S. cerevisiae* and *Y. lipolytica* represented the Saccharomycotina and *S. pombe*, *L. bicolor* and *A. bisporus* served as outgroups.

The phylome, meaning the complete collection of phylogenetic trees for each gene in a genome, was reconstructed in an automated process that mimics a manual phylogenetic tree reconstruction process [184]: homology search, multiple sequence alignment and phylogenetic reconstruction. For each protein encoded in the *P. confluens* genome we performed a Smith-Waterman search against the proteome database formed by the genomes listed above. Results were then filtered using an e-value threshold of 1e-05 and a continuous overlapping region between the query and the result of 0.5. A maximum of 150 sequences were taken. Multiple sequence alignments were then reconstructed using three different programs: MUSCLE v3.8 [185], MAFFT v6.712b [186], and DIALIGN-TX [187]. Alignments were reconstructed in forward and reverse (i.e using the Head or Tail approach [188]). The resulting alignments were then combined using M-COFFEE [189]. A trimming step was performed using trimAl v1.3 [190] (consistency-score cutoff 0.1667, gap-score cutoff 0.9). Trees were reconstructed using PhyML [191]. First the best fitting model was selected by reconstructing neighbor joining trees as implemented in BioNJ [192] using seven different models (JTT, LG, WAG, Blosum62, MtREV, VT and Dayhoff). The two best models in terms of likelihood were used to reconstruct maximum-likelihood trees. Four rate categories were used and invariant positions were inferred from the data. Branch support was computed using an aLRT (approximate likelihood ratio test) based on a chi-square distribution. Resulting trees and alignments are stored in phylomeDB [184] (http://phylomedb.org), with the phylomeID 203.

Orthologs between *P. confluens* and the other species included in the phylome were based on the phylogenetic trees reconstructed in the phylome (Table S9). A species-overlap algorithm, as implemented in ETE v2 [193] was used to infer orthology and paralogy relationships. The algorithm traverses the tree and at each node it calls speciation or duplication depending on whether there are common species at both sides of the node.

Expanded protein families were detected based on the trees reconstructed in the phylome. For each tree, we used ETE v2 [193] to find nodes that exclusively contained *P. confluens* sequences. Only those nodes with more than five sequences were considered as expansions. Overlapping expansions were fused when they shared more than 20% of their members. Expansions were then annotated using a BLAST search against UniProt.

The species tree was build using a concatenation method. 426 single-copy, widespread genes were selected. The concatenated alignment was further trimmed using trimAl [190] (gap-score cutoff 0.5 and conservation 0.5). The final alignment contained 277,192 positions. The tree was reconstructed using PhyML [191]. LG model [194] was selected and a 4-categories GAMMA distribution was used. A bootstrap of 100 repetitions was also reconstructed. Additionally a species tree based on the super-tree reconstruction program DupTree [195] was reconstructed. All the trees reconstructed in the phylome were used as input (6,949 trees). Both species trees showed the same topology.

r8s [49] was used to estimate the divergence between *P. confluens* and *T. melanosporum* based on the species tree inferred from the concatenated alignment. Two analyses were run using different estimates of the divergence between *Schizosaccharomyces pombe* and the remaining acomycetes as calibration point. For each analysis the smoothing parameter was estimated using cross-validation. In the first analysis, the divergence between *S. pombe* and the remaning ascomycetes was put at 723.86 Mya [196] (www.timetree.org), this resulted in a divergence time *between T. melanosporum* and *P. confluens* of 260.38 Mya. If a more ancestral divergence point is selected as a calibration point (1147.78 Mya, [197]), then the divergence time between the two species of interest is 413.30 Mya.

### Quantitative Analysis of Gene Expression Based on RNA-seq Data

RNA-seq reads were cleaned with custom-made Perl scripts as described [24] and mapped to the *P. confluens* genome sequence using Tophat [31]. Custom-made Perl scripts using modules from the BioPerl toolkit [170] were used to determine the number of reads that mapped to each annotated protein-coding gene based on the SAM files with the mapping information (output from Tophat), and quantitative analysis was done with two different methods (“classical” and with LOX [198]) as described previously [24]. We also used the two Bioconductor packages DESeq [199] and baySeq [200] in the R computing environment (version 2.12.1); however, similar to previous analyses, the number of differentially expressed genes was >10%, and under these conditions the statistical models upon which these methods are based are no longer valid [24], therefore the results were not used for further analyses (data not shown). Results from both LOX and “classical” analyses agreed well with qRT-PCR results for selected genes (see e.g. Figure S14, compare qRT-PCR results for LL/DD with RNA-seq results for sex/DD). LOX calculates expression ratios and Bayesian credible intervals and p-values for differential expression. The classical analysis consists of the calculation of expression ratios, standard deviation, and coefficient of variance from read counts normalized to the total number of read counts for the sample, and genes were sorted into five groups (0–4) according to the following criteria: genes in group 4 have ratios of ≤0.25 or ≥4 in all independent biological replicates, genes in group 3 have a mean ratio of ≤0.25 or ≥4 and a coefficient of variance <0.5, genes in group 2 have ratios of ≤0.5 or ≥2 in all independent biological replicates, genes in group 1 have a mean ratio of ≤0.5 or ≥2 and a coefficient of variance <0.5, and group 0 contains all other genes (with the exception of genes for which no ratios could be calculated due to a lack of read coverage, these were not included in the analysis). To classify genes as differentially expressed, a consensus was determined for each gene based on the results from both the classical and LOX analysis; a gene was labeled as up-regulated (1), down-regulated (−1) or not differentially expressed (0) under the conditions that were compared, when the following criteria were met: (a) (“normal” analysis) expression ratios from both classical and LOX analysis had to be >2 and <0.5, LOX Bayesian probability for differential expression = 1, and the gene had to be in groups 1–4 in the classical analysis; (b) (stringent analysis) thresholds for expression ratios were set to >4 and <0.25.

For the analysis of reads that mapped to different genomic regions (e.g., exons, introns, intergenic regions), reads were counted based on the SAM files with the mapping information using custom-made Perl scripts as described previously [24]. To determine the distribution of expression frequencies (Figure S4), the coverage for locus tags of protein-coding gene was determined as the average coverage for the bases of the predicted mRNA (normalized to coverage per kilobase per million counted bases in the sample). Curve fitting and clustering of the data by expectation-maximization was performed on the log_2_-transformed RNA-seq data using the R package mclust [201].

### Complementation of *S. macrospora* Δpro44 with *PCON_06721*

Plasmid pRSnat_06721_OE for complementation of an *S. macrospora pro44* deletion mutant was generated by homologous recombination in yeast as described [102]. It contains the *PCON_06721* ORF under control of the *Aspergillus nidulans gpd* promoter and *trpC* terminator in vector pRSnat, which contains a nourseothricin resistance cassette for selection in *S. macrospora* [202]. The *S. macrospora* Δpro44 strain was transformed with pRSnat_06721_OE as described [104], [203].

### Phylogenetic Analysis

Multiple alignments were created in CLUSTALX [204] and trimmed with Jalview [205], and the same alignment was used for analysis by distance-matrix (DM) or maximum parsimony (MP). Phylogenetic analyses were made with PAUP version 4.0b10 for Windows (D.L. Swofford, distributed by Sinauer Associates, copyright 2001 Smithsonian Institution). DM and MP analyses were performed using 10,000 bootstrap replicates. Consensus trees were graphically displayed with TREEVIEW [206].

### Accession Numbers

The sequence and annotation data are available under the accession numbers HF935090–HF936677 (annotated scaffolds at the European Nucleotide Archive ENA, http://www.ebi.ac.uk/ena/data/view/HF935090-HF936677), CATG01000001–CATG01001898 (primary, non-annotated contigs from which scaffolds were assembled), and BioProject acc. PRJNA65573. The sequence reads that were used for the assembly of the *P. confluens* genome were submitted to the NCBI sequence read archive (accession number SRA059523). The RNA-seq reads and derived expression ratios were submitted to the GEO database (accession number GSE41631).

## Supporting Information

Figure S1 k-mer frequency distribution for *P. confluens* Illumina/Solexa reads. k-mer lengths of k = 31 (top) and k = 41 (bottom) were used for the analysis. A single main peak can be observed in both cases. The rise in k-mer occurrence below a k-mer frequency of 10 is due to sequencing errors. The sum of k-mers from the main peak results in an estimate of ∼50.1 Mb for the size of the haploid genome.
(TIF)

Figure S2 Analysis of genome-wide coverage of different genomic regions by RNA-seq reads. Reads were counted that map to exons of protein-coding genes, introns of protein-coding genes, intergenic regions, and non-coding RNAs (only reads are counted where both ends map to the same type of region). Percent of reads that map to the corresponding regions are shown. At the right end of the graph (separated by a dashed line), the relative distribution of these regions across the genome is indicated.
(TIF)

Figure S3 Repeat divergence analysis. Histograms of percent divergence, insertions, and deletions of *P. confluens* repeat sequences compared to the consensus sequences of the respective repeats as determined by RepeatMasker based on a RepeatModeler-generated, *P. confluens*-specific repeat library. For this analysis, only repeats >200 bp (without simple repeats and low complexity regions) were chosen, a total of 7,158 repeats were analyzed. Only few repeats show perfect or high identity to the consensus sequence (i.e. low sequence divergence); the majority of reads shows between 10–20% divergence compared to the consensus sequence (mean 15.20%, median 16.73%), suggesting that they are not of recent origin. However, few deletions or insertions are observed in the repeat sequences.
(TIF)

Figure S4 Distribution of gene expression levels. **A.** Histograms of log_2_ of coverage (normalized to base coverage per kilobase of mRNA per million counted bases) for each locus tag for each independent biological replicate (left, middle), and histograms and estimated frequency distribution functions for the log_2_ of the mean for each condition (right). In case of zero coverage, log_2_ coverage was set to −11 (otherwise all log_2_ values >−11) and was not used here. The distribution function (red) for each condition could be dissected into components. The components (blue, green, and yellow lines) are normal distributions with varying means and variances that make up different proportions of the observed distribution. Estimation of mixtures was done with the mclust package from R (Fraley and Raftery, J Amer Stat Assoc 2002, 97: 611–631) and some manual curve adjustments. **B.** Venn diagrams of the number of genes that contribute to the low expression peak (peak 1, green) and the high expression peak (peak 2, blue) in each condition in A.
(TIF)

Figure S5 Synteny analysis with other fungi. **A.** Regions of sequence identity between the *in silico*-translated genomic sequences of the pairs *T. melanosporum*/*P. confluens* and *S. macrospora*/*N. crassa* (for comparison) were determined with the PROmer algorithm from the MUMmer package [47]. The dot plot was plotted with gnuplot, red indicates sequences on the forward strand, blue on the reverse strand of the reference (x-axis). The PROmer analysis also shows much greater synteny between *S. macrospora* and *N. crassa* than between *T. melanosporum* and *P. confluens*. **B.** Positions of orthologous genes were determined along the concatenated scaffolds from each species and visualized as dot plot for the pairs *T. melanosporum*/*P. confluens* and *S. macrospora*/*N. crassa* (for comparison). Scaffolds were not specifically ordered for this analysis, therefore the seemingly still somewhat random organization in the *N. crassa*/*S. macrospora* comparison (as compared to the corresponding Figure in [22]); however, this was done on purpose to show the difference between the *S. macrospora*/*N. crassa* and *T. melanosporum*/*P. confluens* comparisons, even though the number of scaffolds in *S. macrospora* and *P. confluens* is very similar (1,583 vs. 1,588 scaffolds in the assemblies used for this comparison).
(TIF)

Figure S6 Lineage-specific peptide lengths and gene expression based on phylogenomics analysis and RNA-seq data. Peptide lengths and expression of *P. confluens* genes from groups of genes with different levels of evolutionary conservation as indicated. Lineage-specificity groups were determined based on phylogenomics analyses (Tables S8 and S9). **A.** Boxplot showing the distribution of peptide lengths (outliers left out for better visibility) with the median value as a horizontal line in the box between the first and third quartiles. Peptide length of predicted *P. confluens* orphan genes are smaller than those for the other groups, and from groups A to D, median peptide lengths increase with increasing conservation of genes. **B.** The number of genes in each group are indicated on the logarithmic y-axis to the right, and percentage of genes that are differentially regulated under any condition, and up- or down-regulated during sexual development (up or down in sex/DD and sex/vegmix, data shown for stringent expression analysis) are indicated on the y-axis to the left. The orphan genes have the highest percentage of differentially expressed genes, most of which are upregulated during sexual development, and this can also be observed in the Pezizales-specific genes (group B). In the other groups, the portion of differentially regulated genes is smaller, and the percentage of genes upregulated during sexual development is similar or smaller than that of downregulated genes. **C.** Overall expression levels given in RPKM (reads per kilobase per million counted reads). For each group, RPKM values were calculated for samples sexual development (S), DD (D), and vegmix (V) as mean RPKM values of the two independent experiments. The boxplot shows the distribution with the median value as a horizontal line in the box between the first and third quartiles (outliers left out for better visibility). The small inlet shows a magnification of the RPKM values for group A (orphan genes) for better visualization of the higher mean in the samples for sexual development.
(TIF)

Figure S7 Fruiting body development under different light regimes and on different media. *P. confluens* was grown on minimal medium or cornmeal medium for 7 d at 25°C. Light regimes were constant white light (LL), constant darkness (DD) and 12 h light/12 h darkness cycles (LD). Fruiting bodies are only formed on minimal medium with illumination (LL or LD), whereas on complete medium, there is orange pigmentation of the mycelium, but no fruiting body formation. Under conditions that do not allow sexual development, sometimes mycelial aggreagates are formed, but do not contain sexual structures.
(TIF)

Figure S8 Transmission data for filters used in light experiments. The following LEE filters (Hampshire, UK) were used, transmitted light curves from http://www.leefilters.com/lighting/colour-list.html: blue: http://www.leefilters.com/lighting/colour-details.html#363&filter=cf, green: http://www.leefilters.com/lighting/colour-details.html#139&filter=cf, red: http://www.leefilters.com/lighting/colour-details.html#106&filter=cf, far-red: http://www.leefilters.com/lighting/colour-details.html#787&filter=cf. Curves indicate transmitted light (in %) for each wavelength.
(TIF)

Figure S9 Alternative splicing of the *P. confluens frq* gene (*PCON_09365*). **A.** The first 1500 nucleotides of the *frq* transcript are shown. The upper part shows the major transcript and the lower part shows a minor transcript generated through alternative splicing as identified by the presence of spliced sequence reads in the RNA-seq data. The minor transcript lacks the predicted AUG and would therefore use a downstream AUG and yield a protein that lacks the first 32 amino acids. Three putative upstream ORFs are indicated as black rectangles. Numbers indicate nucleotide positions in scaffold 447. **B.** Semi-quantitative RT-PCR analysis of alternative splicing. PCR primers (PCON_09365_t1/t3, Table S15) are indicated by gray arrows below the transcripts in A. (+) and (−) indicate RT-PCRs with and without reverse transcriptase, respectively, with the (−) samples showing no amplicons as expected. NTC, no template control. The 500 bp and 300 bp amplicons represent the major and the minor (alternatively spliced) transcript, respectively. The minor transcript is barely detectable in DD, but clearly present under light conditions, both in LL (4 d of constant light) and after a 30 min light pulse (DD+30). **C.** Quantification of the ratio of spliced versus non-spliced transcripts (300 bp versus 500 bp RT-PCR products in B). Mean and standard deviation of two independent biological replicates are shown.
(TIF)

Figure S10 Expression of CBM_14 domain-encoding genes in *P. confluens* as determined by RNA-seq. **A.** Expression of CBM_14 domain-encoding proteins during sexual development and vegetative growth. Heatmap of log_2_ ratios of gene expression under three different conditions. Gray boxes indicate non-determined values. C, ratio from “classical” analysis; L, ratio from LOX analysis. Nearly half of the CBM_14 domain-encoding genes are upregulated during sexual development. **B.** Overall expression levels vary between CBM_14 domain-encoding genes. Normalized read counts per kb of mRNA (mean of two independent biological replicates) are shown for the three conditions for each gene. Overall read counts per condition vary from none to >13000.
(TIF)

Figure S11 Defensin_2 domain-encoding genes in *P. confluens*. **A.** Multiple Alignment of Defensin_2 domain-containing proteins from *P. confluens* and *Pseudoplectania nigrella*. Two of the *P. confluens* genes are pseudogenes that are not expected to yield a functional protein; the stop codon within PCON_01607 and the two amino acids between which a frameshift occurs in PCON_01611 are indicated in red. The predicted signal sequences for co-translational insertion into the ER are given in bold (SignalP 4.0 predictions, Nature Methods 2011, 8:785–786). The position of the conserved intron within the coding region of all *P. confluens* proteins is indicated by a green triangle above the sequences (the intron is located between the second and third nucleotide of the corresponding nucleotide triplet). Accession number of the *P. nigrella* plectasin: sp|Q53I06.1. **B.** Genomic organization of Defensin_2 domain-encoding genes. The genes are located within a region from nt 102001 to nt 113000 of scaffold 1117. The two pseudogenes *PCON_01607* and *PCON_01611* are shaded in gray. **C.** Expression of Defensin_2 domain-containing genes. Normalized counts of RNA-seq reads for the three conditions that were investigated (sex, DD, and vegmix) are shown for the six genes; note that the read counts for sexual development are given on the secondary axis to the right, because they are much lower than the read counts in DD and vegmix for all six genes. Read counts for the two pseudogenes *PCON_01607* and *PCON_01611* (shaded in gray) are generally lower than those for the other genes in all conditions investigated. **D.** Phylogenetic analysis of Defensin_2 domain-encoding proteins. In addition to the *P. confluens* proteins, the following proteins were used for Neighbor joining analysis with 1000 bootstrap replications: A.cap, *Ajellomyces capsulatus* ref|XP_001537899.1; A.der., *Ajellomyces dermatitides* gb|EEQ83322.1; ANID_05046 and ANID_11510, *Aspergillus nidulans* from the *A. nidulans* genome project http://www.broadinstitute.org/annotation/genome/aspergillus_group/MultiHome.html; T.rub, *Trichophyton rubrum* ref|XP_003237583.1; P. nig, *Pseudoplectania nigrella* sp|Q53I06.1.
(TIF)

Figure S12 Genes for polyketide and non-ribosomal peptide biosynthesis in *P. confluens*. **A.** Protein domain organization of all proteins in *P. confluens* that are predicted to be PKSs or NRPSs. Complete A-T-C modules in the NRPSs are boxed. The putative siderophore biosynthesis NRPS PCON_02859 has the typical domain structure of four A-T-C modules and two T-C modules. The five AAR-type NRPSs (domain structures A-T-R) are shown in a gray box. **B.** Putative biosynthetic cluster containing the NRPS gene *PCON_02859*. Genes with putative functions in siderophore biosynthesis are shown in light blue. **C.** Putative biosynthetic gene cluster containing the PKS gene *PCON_04029* and the NRPS gene *PCON_04030*. Genes with putative functions in the biosynthesis of a hybrid polyketide/non-ribosomal peptide are shown in light blue.
(TIF)

Figure S13 Differentially expressed predicted transcription factors in *P. confluens*. **A.** 54 of the 177 predicted transcriptions factors are differentially expressed in at least one comparison (sex/DD, sex/vegmix, DD/vegmix; data from non-stringent analysis); these are shown in this heatmap (clustering and heatmap generation in R). C and L show log_2_ expression ratios from classical and LOX analysis, respectively. **B.** Eight putative transcription factors are strongly upregulated (>10×, up to ∼4000×) during sexual development in both sex/DD and sex/vegmix. They contain domains from seven different DNA binding domain families. Only PCON_07392 and PCON_02619 have an ortholog in most investigated fungi, for the others, there are often BLAST hits, but no clear orthologs (in reciprocal BLAST). One reason for this might be that these transcription factors belong to larger families where the definition of orthologs is difficult. PCON_02619 is the ortholog of the *S. macrospora*/yeast STE12 transcription factor.
(TIF)

Figure S14 Quantitative real time PCR analysis of selected putative transcription factor genes in *P. confluens*. Expression was analyzed by qRT-PCR for each gene in two independent biological replicates for the four conditions LL, DD, LLK and DDK (light and darkness in surface culture and submerged culture). Sexual development is only possible in condition LL. Expression ratios are shown as blue bars, and were calculated to address the question if a gene is differentially regulated during sexual development (i.e. in LL/DD and in LL/LLK), or regulated by light (i.e. in LL/DD and LLK/DDK) or regulated by surface versus submerged growth (i.e. LL/LLK and DD/DDK). RNA-seq results for each gene are given as gray bars for comparison: light gray, LOX; dark gray, classic analysis. Dashed lines indicate two-fold upregulation. The qRT-PCR results match the RNA-seq results very well, this can be seen in the comparison sex/DD and LL/DD (same growth conditions, qRT-PCR 3 d, RNA-seq: pool of 3–5 d; the other growth conditions are not directly comparable, but tendencies are conserved). The qRT-PCR data confirm a strong upregulation of *PCON_02619*, *PCON_04765*, and *PCON_06047* during sexual development only (as opposed to light- or surface culture-dependent, because LL/LLK also gives upregulation which indicates independence of light, and DD/DDK does not give upregulation which indicates that surface culture alone is not sufficient for upregulation). The three genes *PCON_10922*, *PCON_10987*, and *PCON_10990* are not significantly upregulated by a single condition (sexual development, light, surface culture). *PCON_10922* is upregulated in the comparison LL/LLK, but not LL/DD or DD/DDK. This might indicate that surface culture induces the expression of this gene, but only in the light.
(TIF)

Figure S15 Comparative analysis of gene expression during sexual development in *P. confluens* (*P.c.*) and *S. macrospora* (*S.m.*). Heat map of hierarchical clustering of log_2_ expression ratios for comparison of different growth conditions in both fungi.
(TIF)

Figure S16 Gene structure and alignments of *pro44* homologs. **A.** Genomic loci and predicted proteins for *P. confluens pro44* (*PCON_06721*) and its orthologs in *S. macrospora* (*pro44*, *SMAC_03223*), *N. crassa* (*sub-1*, *NCU01154*), and *A. nidulans* (*nsdD*, *ANID_03152*). Lengths of coding sequences and proteins are given to the right. **B.** Multiple alignment of the four proteins. The conserved GATA domain at the C-terminus is indicated by a black line above the sequence. It includes four conserved cysteine residues that bind a zinc atom to form the zinc finger. Data from the following sources: *S. macrospora*: acc. no. CABT02000007, *N. crassa*: *Neurospora crassa* database (http://www.broadinstitute.org/annotation/genome/neurospora/MultiHome.html), *A. nidulans*: acc. no. U70044 and Aspergillus comparative database (http://www.broadinstitute.org/annotation/genome/aspergillus_group/MultiHome.html).
(TIF)

Table S1 Overview of DNA and RNA sequencing results for the *P. confluens* genome and transcriptome. **A.** Roche/454 sequencing of genomic DNA. **B.** Illumina/Solexa sequencing of genomic DNA (50 base reads from 300 bp insert paired-end library). **C.** RNA-seq analysis (Illumina/Solexa sequencing of 101 base reads from 300 bp insert paired-end cDNA library).
(PDF)

Table S2 Analysis of differential gene expression in *P. confluens* by RNA-seq. The table gives an overview of all protein-coding genes of *P. confluens* with their basic characteristics (length of mRNA, UTRs, CDS, peptides; homology to other species; putative domains and subcellular localization), normalized RNA-seq read counts from two replicates each of the condition sex, DD, and vegmix, as well as the expression ratios for sex/DD, sex/vegmix, and DD/vegmix calculated with LOX or “classic” analysis as well as results of the consensus analysis.
(XLSX)

Table S3 Antisense splice sites in RNA-seq reads.
(XLSX)

Table S4 Repeat elements in the *P. confluens* genome.
(XLSX)

Table S5 *P. confluens* homologs of chromatin-associated proteins and proteins involved in genome defense. **A.** Histones and histone modification. **B.** DNA methylation machinery. **C.** RNA interference and meiotic silencing.
(PDF)

Table S6 Predicted regions that were subjected to RIP.
(XLSX)

Table S7 Mating type, pheromone, pheromone receptor, pheromone-processing, and signal transduction genes, and homologs to developmental genes in *P. confluens*. **A.** Genes within or adjacent to the mating type loci. Genes that are upregulated at least two-fold in both classic (C) and LOX (L) analysis in the conditions investigated are given in bold red. **B.** Putative pheromone processing proteins. Genes that are upregulated at least two-fold in both classic (C) and LOX (L) analysis in the conditions investigated are given in bold red. **C.** Putative proteins of the pheromone response pathway. Genes that are upregulated at least two-fold in both classic (C) and LOX (L) analysis in the conditions investigated are given in bold red. **D.** Putative MAP kinase modules. **E.** *P. confluens* homologs to developmental proteins from *S. macrospora*.
(PDF)

Table S8 Lineage-specific gene expression. The table contains four sheets that give a summary as well as expression and orthology information for *P. confluens* genes of groups a-f with orthology determined by reciprocal BLAST in the first two sheets, and a summary and Expression and orthology information for *P. confluens* genes of groups A–G with orthology determined by phylogenomics analysis in the last two sheets.
(XLSX)

Table S9 Overview of phylome reconstruction.
(XLSX)

Table S10 Gene expression under different light regimes. Transcript levels were determined after short term light induction (5–60 min after growth in darkness for 4 d). *P. confluens* was grown in minimal liquid medium and harvested under far-red light. Transcript levels were determined by qRT-PCR from at least two independent biological replicates, ratios versus 4 d in darkness (DD) and standard deviations are given.
(PDF)

Table S11 Analysis of gene families and gene family expansion and contraction in *P. confluens* compared to other fungi. The first sheet contains results from pfam domain searches, the second sheet results from phylogenomic analysis, and the third sheet contains gene families that are contracted in *P. confluens* in comparison with other filamentous ascomycetes.
(XLSX)

Table S12 *P. confluens* homologs to heterokaryon incompatibility genes from *N. crassa* and *P. anserina*. For *N. crassa* and *P. anserina* proteins shaded in gray, no clear *P. confluens* ortholog can be identified. **A.** *N. crassa het* genes. **B.** *P. anserina het* genes.
(PDF)

Table S13 Expression data for predicted *P. confluens* transcription factor genes.
(XLSX)

Table S14 Comparative transcriptomics of orthologs in *P. confluens* and *S. macrospora*. *S. macrospora* data are from [24], *P. confluens* data from this study.
(XLSX)

Table S15 Oligonucleotides used as primers for qRT-PCR analysis.
(PDF)

Text S1 Distribution of gene expression levels.
(PDF)

Text S2 Genes for pheromone/receptor signaling.
(PDF)
