# Supplementary figures and images for: The Genome and Development-Dependent Transcriptomes of Pyronema confluens: A Window into Fungal Evolution
Source: PLoS Genet. 2013 Sep 19;9(9):e1003820. doi: 10.1371/journal.pgen.1003820 (PMC3778014; doi:10.1371/journal.pgen.1003820)

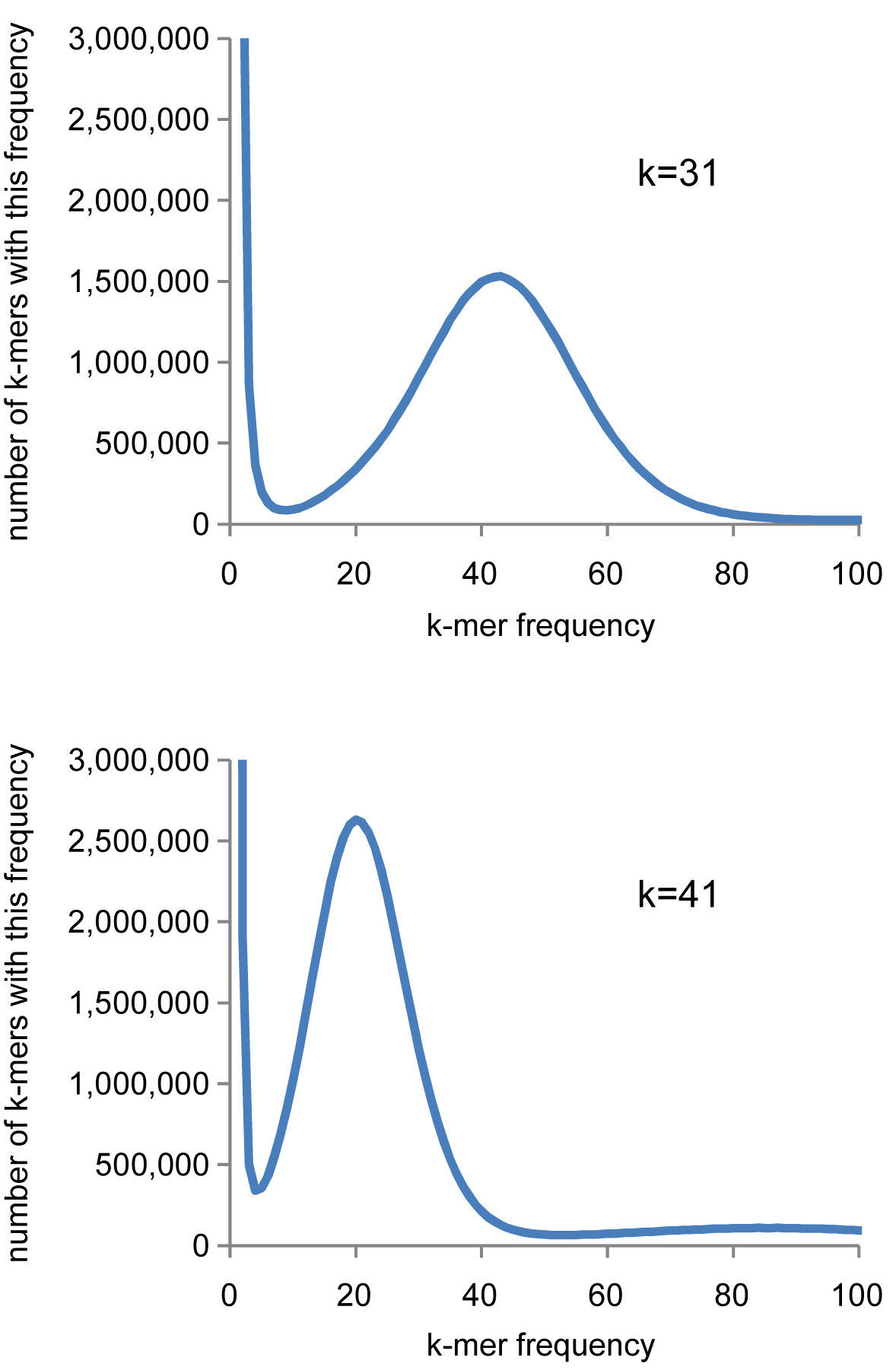

Supplement: Figure S1 — k-mer frequency distribution for P. confluens Illumina/Solexa reads. k-mer lengths of k = 31 (top) and k = 41 (bottom) were used for the analysis. A single main peak can be observed in both cases. The rise in k-mer occurrence below a k-mer frequency of 10 is due to sequencing errors. The sum of k-mers from the main peak results in an estimate of ∼50.1 Mb for the size of the haploid genome. (TIF) [file pgen.1003820.s001.tif]

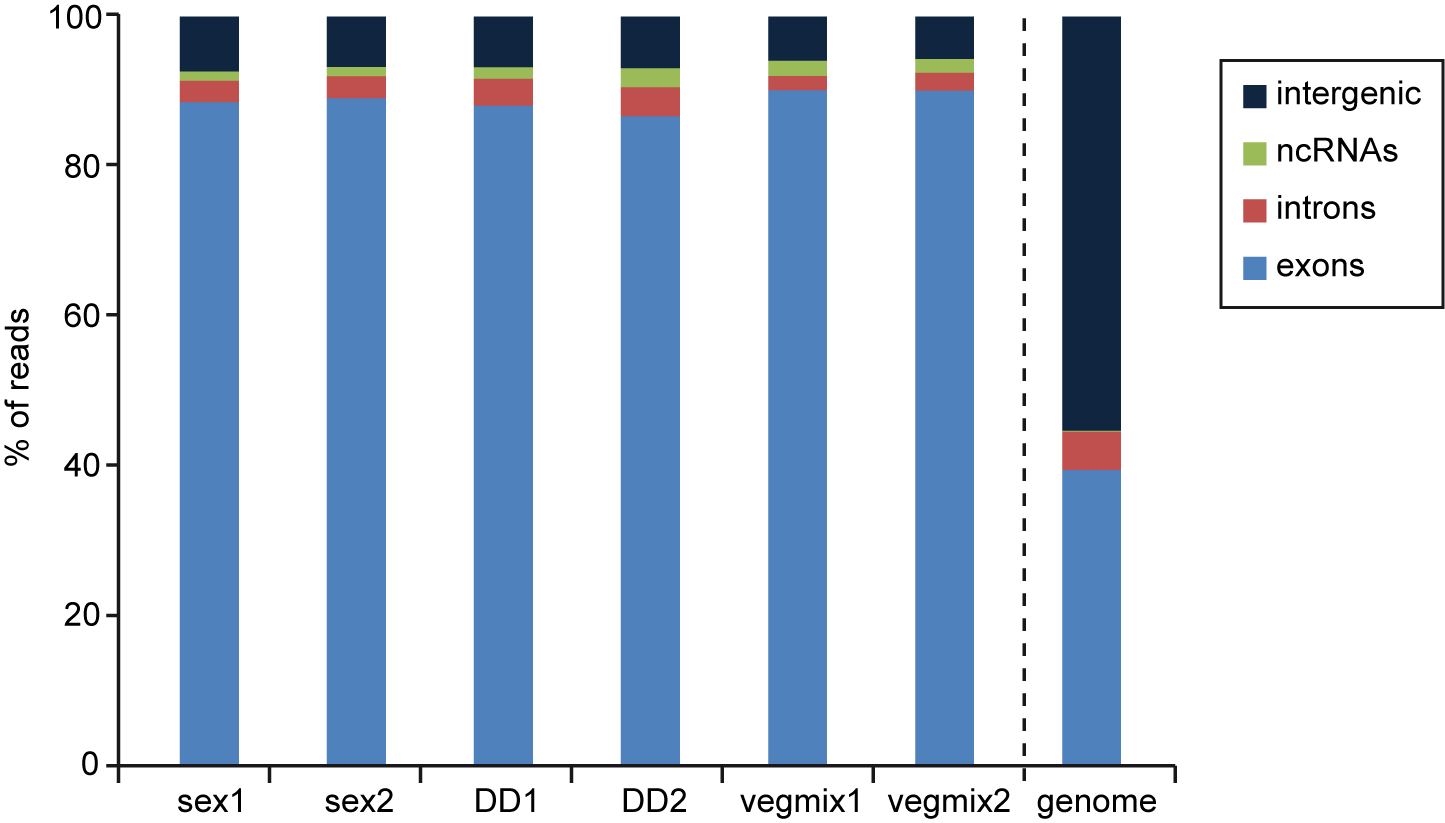

Supplement: Figure S2 — Analysis of genome-wide coverage of different genomic regions by RNA-seq reads. Reads were counted that map to exons of protein-coding genes, introns of protein-coding genes, intergenic regions, and non-coding RNAs (only reads are counted where both ends map to the same type of region). Percent of reads that map to the corresponding regions are shown. At the right end of the graph (separated by a dashed line), the relative distribution of these regions across the genome is indicated. (TIF) [file pgen.1003820.s002.tif]

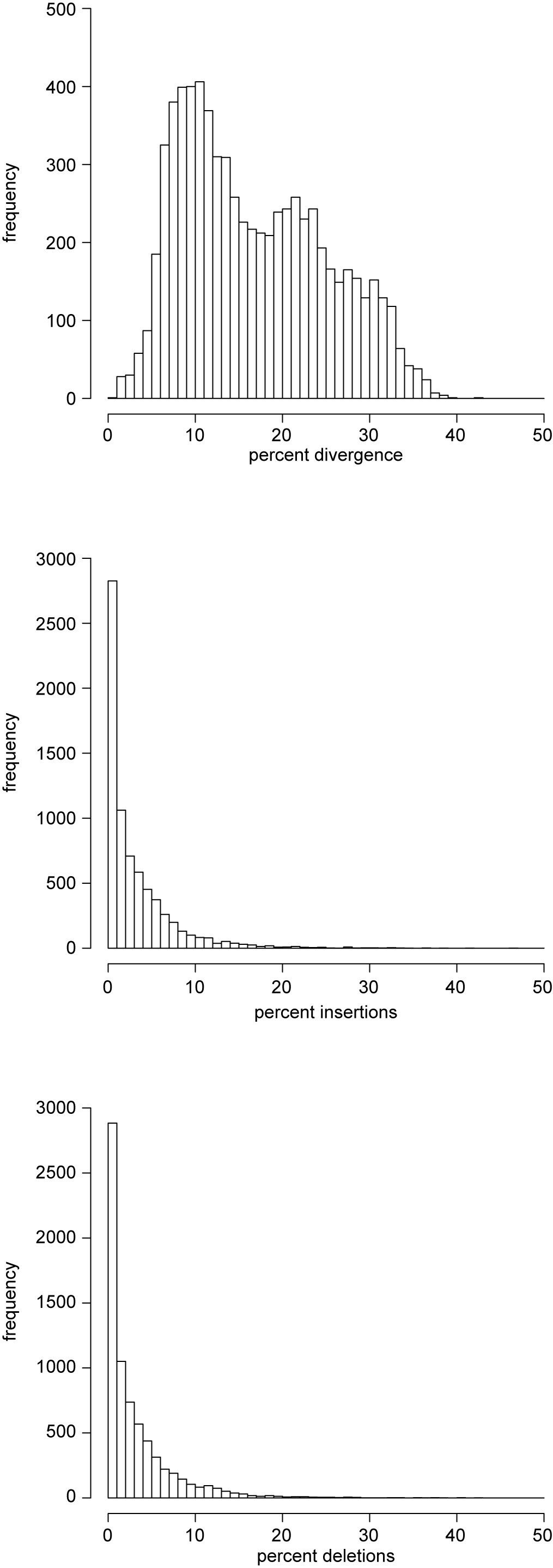

Supplement: Figure S3 — Repeat divergence analysis. Histograms of percent divergence, insertions, and deletions of P. confluens repeat sequences compared to the consensus sequences of the respective repeats as determined by RepeatMasker based on a RepeatModeler-generated, P. confluens-specific repeat library. For this analysis, only repeats >200 bp (without simple repeats and low complexity regions) were chosen, a total of 7,158 repeats were analyzed. Only few repeats show perfect or high identity to the consensus sequence (i.e. low sequence divergence); the majority of reads shows between 10–20% divergence compared to the consensus sequence (mean 15.20%, median 16.73%), suggesting that they are not of recent origin. However, few deletions or insertions are observed in the repeat sequences. (TIF) [file pgen.1003820.s003.tif]

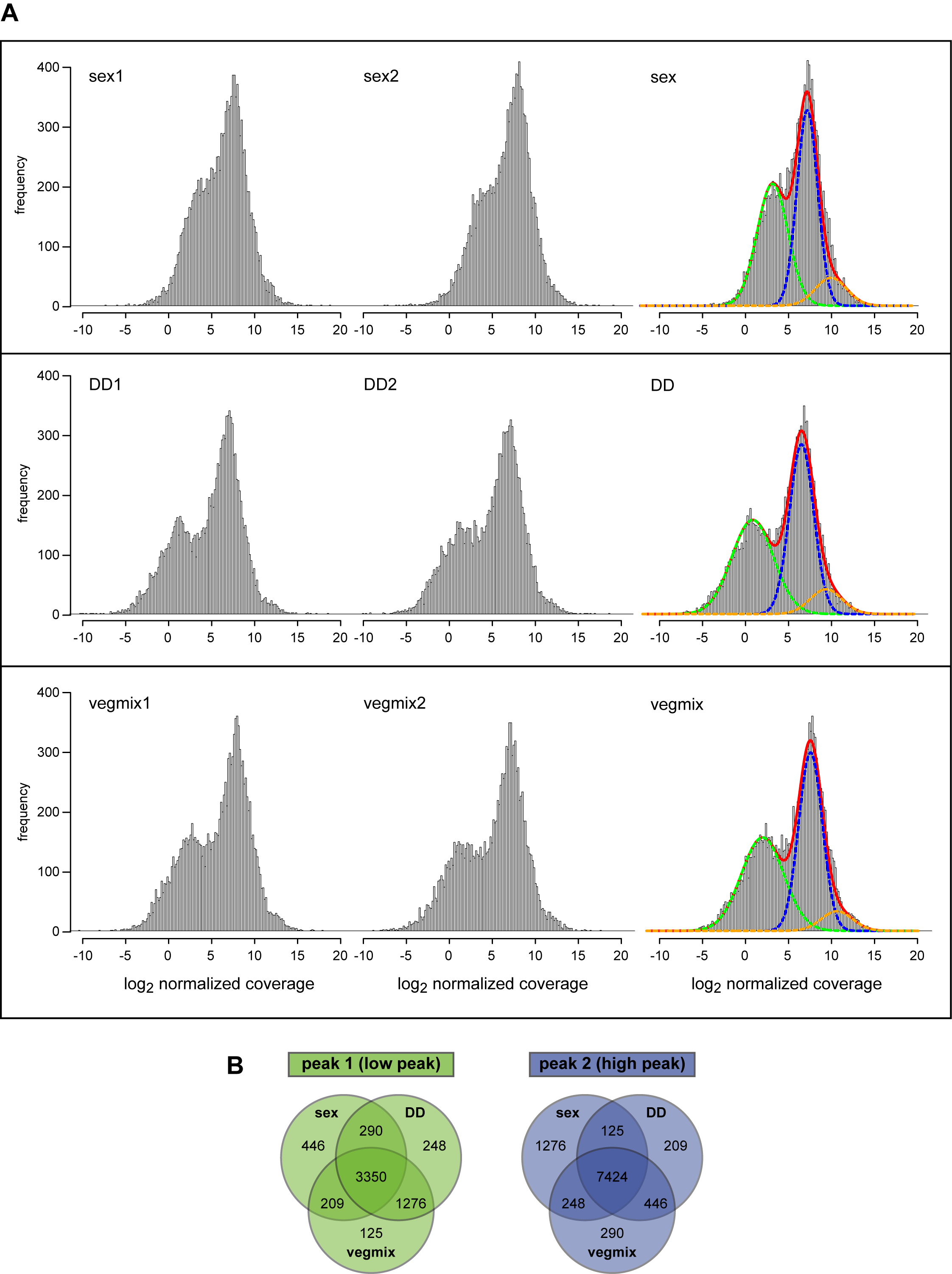

Supplement: Figure S4 — Distribution of gene expression levels. A. Histograms of log2 of coverage (normalized to base coverage per kilobase of mRNA per million counted bases) for each locus tag for each independent biological replicate (left, middle), and histograms and estimated frequency distribution functions for the log2 of the mean for each condition (right). In case of zero coverage, log2 coverage was set to −11 (otherwise all log2 values >−11) and was not used here. The distribution function (red) for each condition could be dissected into components. The components (blue, green, and yellow lines) are normal distributions with varying means and variances that make up different proportions of the observed distribution. Estimation of mixtures was done with the mclust package from R (Fraley and Raftery, J Amer Stat Assoc 2002, 97: 611–631) and some manual curve adjustments. B. Venn diagrams of the number of genes that contribute to the low expression peak (peak 1, green) and the high expression peak (peak 2, blue) in each condition in A. (TIF) [file pgen.1003820.s004.tif]

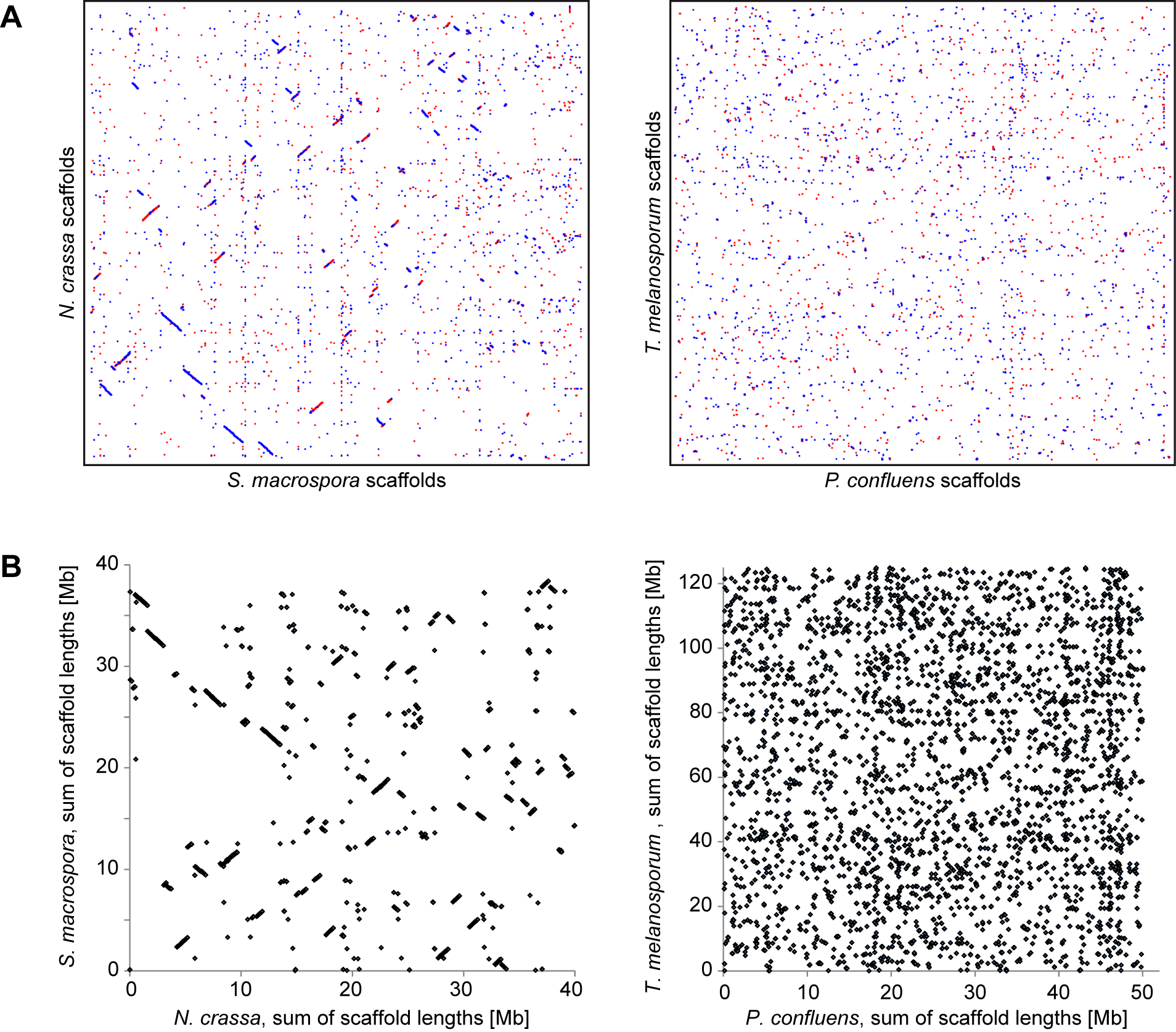

Supplement: Figure S5 — Synteny analysis with other fungi. A. Regions of sequence identity between the in silico-translated genomic sequences of the pairs T. melanosporum/P. confluens and S. macrospora/N. crassa (for comparison) were determined with the PROmer algorithm from the MUMmer package [47]. The dot plot was plotted with gnuplot, red indicates sequences on the forward strand, blue on the reverse strand of the reference (x-axis). The PROmer analysis also shows much greater synteny between S. macrospora and N. crassa than between T. melanosporum and P. confluens. B. Positions of orthologous genes were determined along the concatenated scaffolds from each species and visualized as dot plot for the pairs T. melanosporum/P. confluens and S. macrospora/N. crassa (for comparison). Scaffolds were not specifically ordered for this analysis, therefore the seemingly still somewhat random organization in the N. crassa/S. macrospora comparison (as compared to the corresponding Figure in [22]); however, this was done on purpose to show the difference between the S. macrospora/N. crassa and T. melanosporum/P. confluens comparisons, even though the number of scaffolds in S. macrospora and P. confluens is very similar (1,583 vs. 1,588 scaffolds in the assemblies used for this comparison). (TIF) [file pgen.1003820.s005.tif]

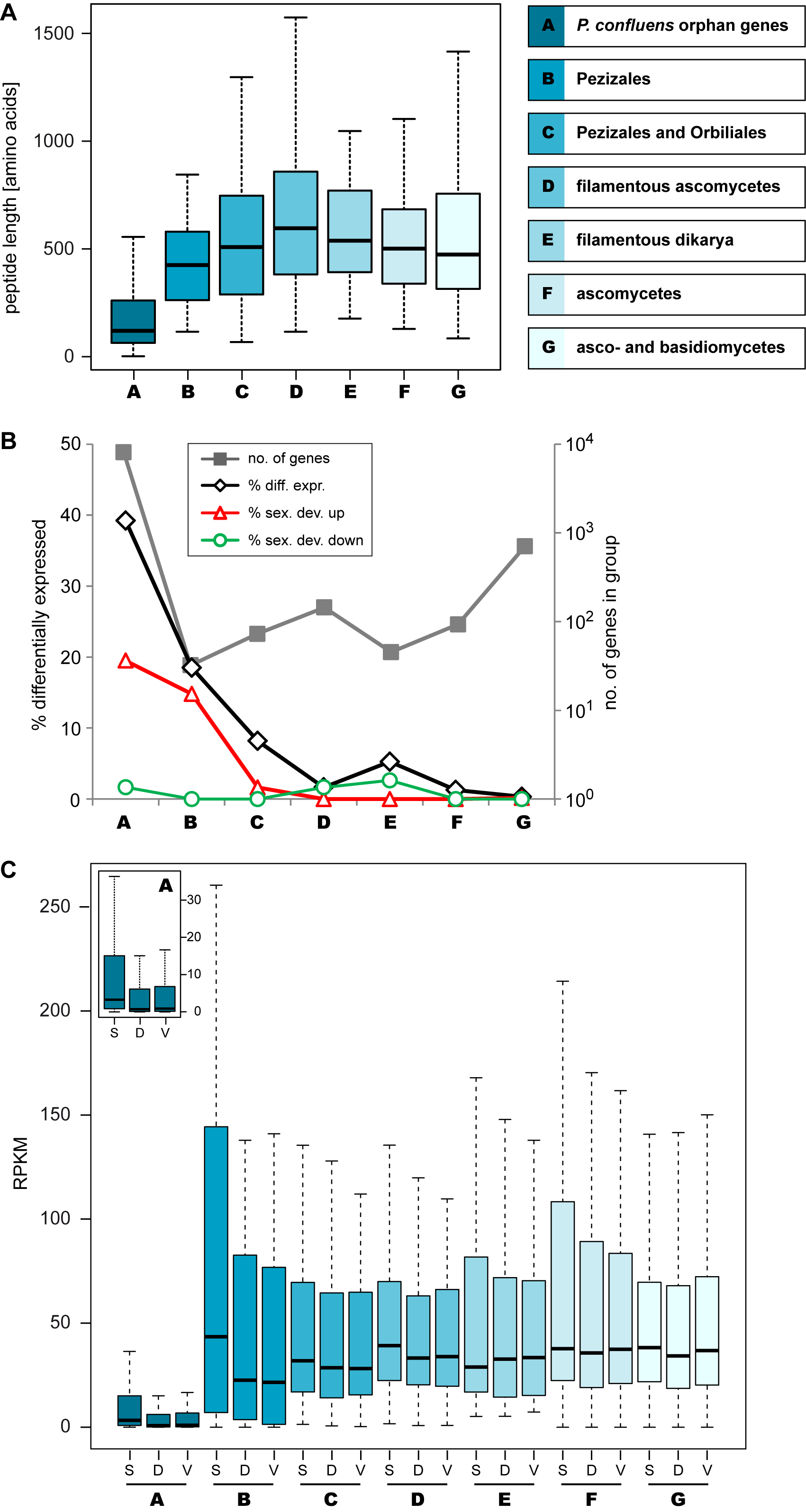

Supplement: Figure S6 — Lineage-specific peptide lengths and gene expression based on phylogenomics analysis and RNA-seq data. Peptide lengths and expression of P. confluens genes from groups of genes with different levels of evolutionary conservation as indicated. Lineage-specificity groups were determined based on phylogenomics analyses (Tables S8 and S9). A. Boxplot showing the distribution of peptide lengths (outliers left out for better visibility) with the median value as a horizontal line in the box between the first and third quartiles. Peptide length of predicted P. confluens orphan genes are smaller than those for the other groups, and from groups A to D, median peptide lengths increase with increasing conservation of genes. B. The number of genes in each group are indicated on the logarithmic y-axis to the right, and percentage of genes that are differentially regulated under any condition, and up- or down-regulated during sexual development (up or down in sex/DD and sex/vegmix, data shown for stringent expression analysis) are indicated on the y-axis to the left. The orphan genes have the highest percentage of differentially expressed genes, most of which are upregulated during sexual development, and this can also be observed in the Pezizales-specific genes (group B). In the other groups, the portion of differentially regulated genes is smaller, and the percentage of genes upregulated during sexual development is similar or smaller than that of downregulated genes. C. Overall expression levels given in RPKM (reads per kilobase per million counted reads). For each group, RPKM values were calculated for samples sexual development (S), DD (D), and vegmix (V) as mean RPKM values of the two independent experiments. The boxplot shows the distribution with the median value as a horizontal line in the box between the first and third quartiles (outliers left out for better visibility). The small inlet shows a magnification of the RPKM values for group A (orphan genes) for better visual [file pgen.1003820.s006.tif]

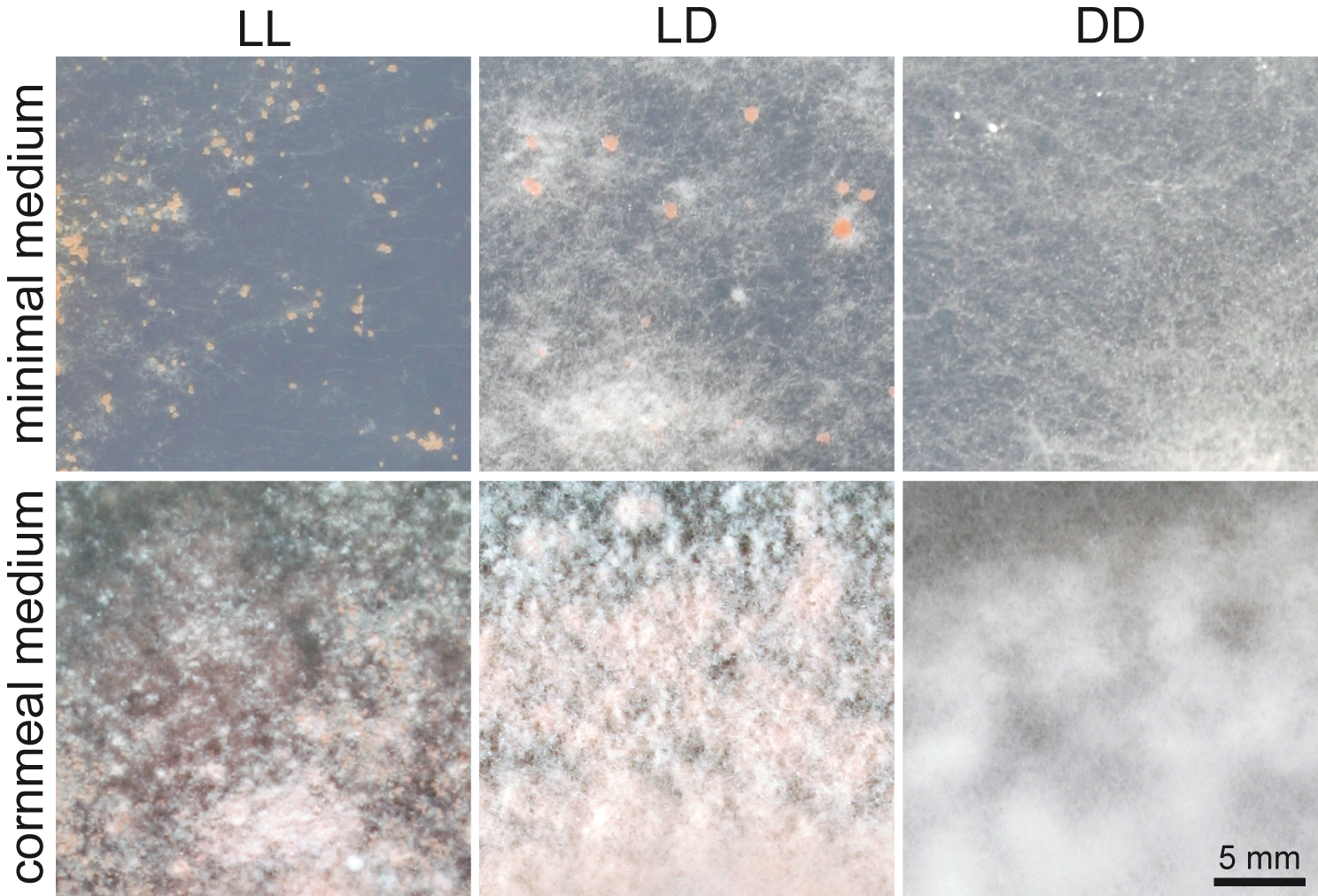

Supplement: Figure S7 — Fruiting body development under different light regimes and on different media. P. confluens was grown on minimal medium or cornmeal medium for 7 d at 25°C. Light regimes were constant white light (LL), constant darkness (DD) and 12 h light/12 h darkness cycles (LD). Fruiting bodies are only formed on minimal medium with illumination (LL or LD), whereas on complete medium, there is orange pigmentation of the mycelium, but no fruiting body formation. Under conditions that do not allow sexual development, sometimes mycelial aggreagates are formed, but do not contain sexual structures. (TIF) [file pgen.1003820.s007.tif]

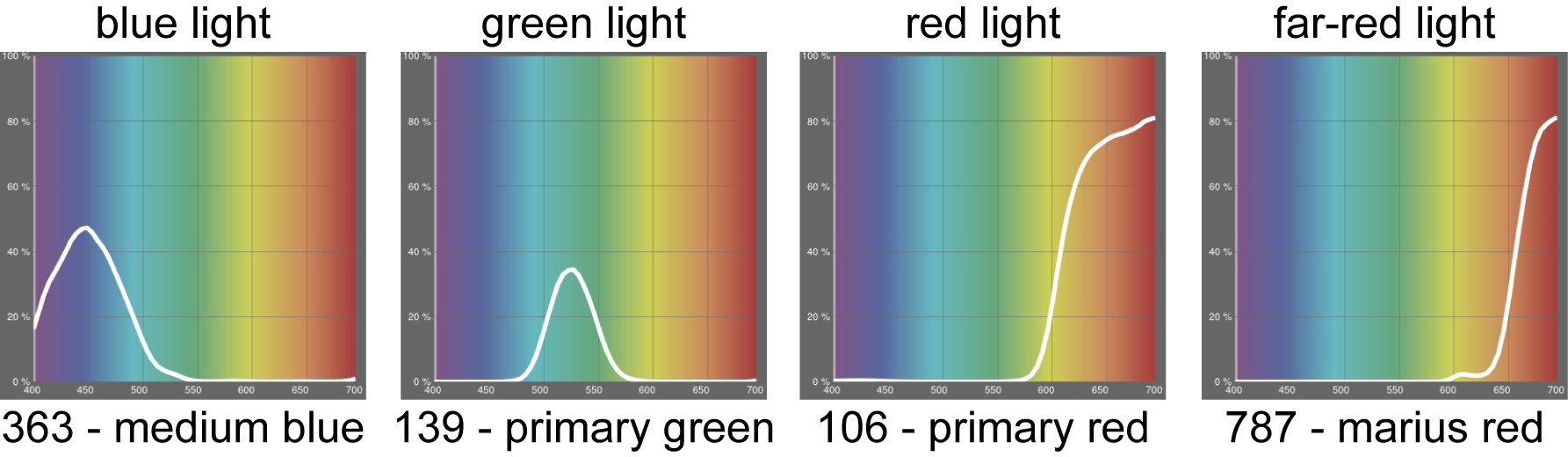

Supplement: Figure S8 — Transmission data for filters used in light experiments. The following LEE filters (Hampshire, UK) were used, transmitted light curves from http://www.leefilters.com/lighting/colour-list.html: blue: http://www.leefilters.com/lighting/colour-details.html#363&filter=cf, green: http://www.leefilters.com/lighting/colour-details.html#139&filter=cf, red: http://www.leefilters.com/lighting/colour-details.html#106&filter=cf, far-red: http://www.leefilters.com/lighting/colour-details.html#787&filter=cf. Curves indicate transmitted light (in %) for each wavelength. (TIF) [file pgen.1003820.s008.tif]

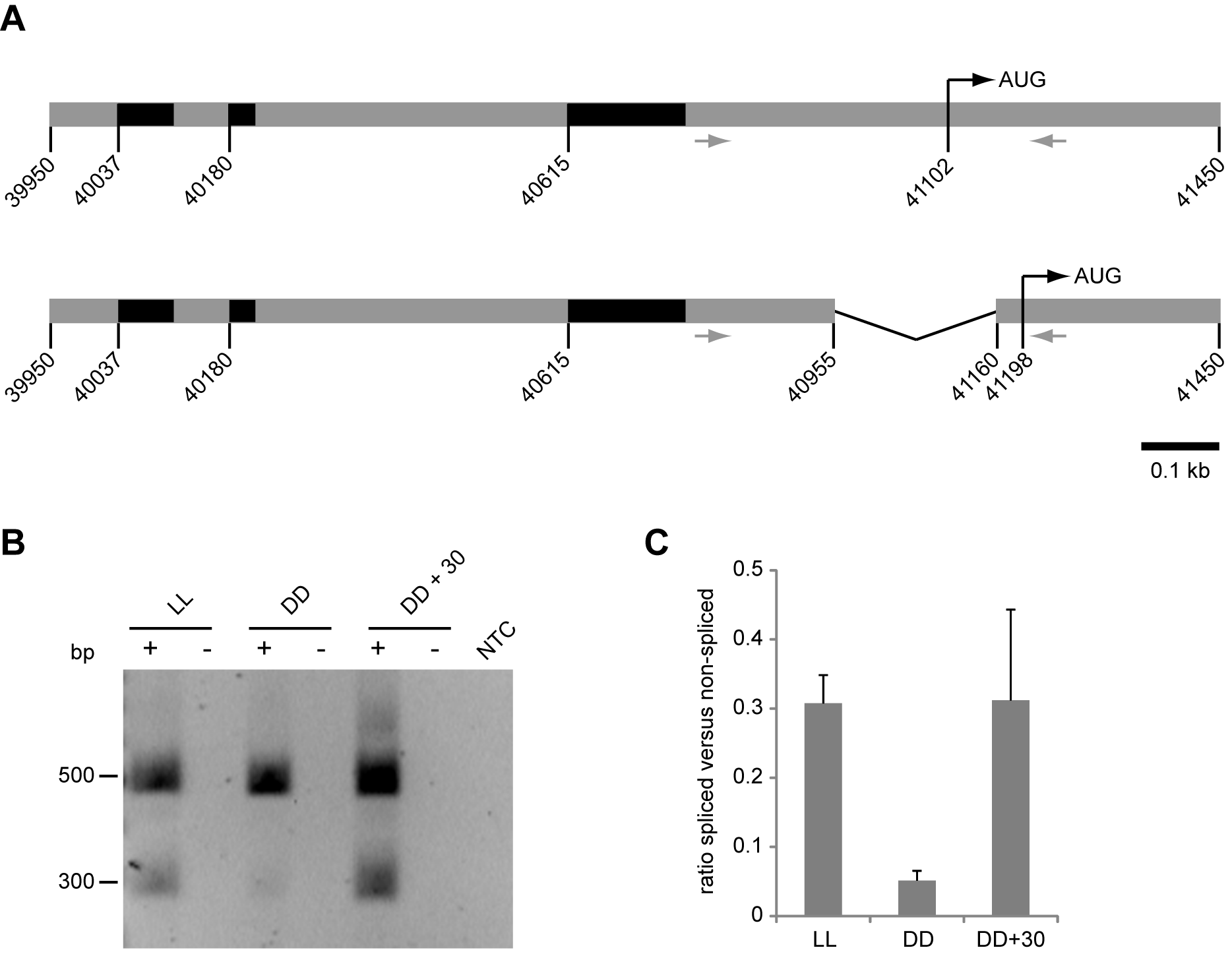

Supplement: Figure S9 — Alternative splicing of the P. confluens frq gene (PCON_09365). A. The first 1500 nucleotides of the frq transcript are shown. The upper part shows the major transcript and the lower part shows a minor transcript generated through alternative splicing as identified by the presence of spliced sequence reads in the RNA-seq data. The minor transcript lacks the predicted AUG and would therefore use a downstream AUG and yield a protein that lacks the first 32 amino acids. Three putative upstream ORFs are indicated as black rectangles. Numbers indicate nucleotide positions in scaffold 447. B. Semi-quantitative RT-PCR analysis of alternative splicing. PCR primers (PCON_09365_t1/t3, Table S15) are indicated by gray arrows below the transcripts in A. (+) and (−) indicate RT-PCRs with and without reverse transcriptase, respectively, with the (−) samples showing no amplicons as expected. NTC, no template control. The 500 bp and 300 bp amplicons represent the major and the minor (alternatively spliced) transcript, respectively. The minor transcript is barely detectable in DD, but clearly present under light conditions, both in LL (4 d of constant light) and after a 30 min light pulse (DD+30). C. Quantification of the ratio of spliced versus non-spliced transcripts (300 bp versus 500 bp RT-PCR products in B). Mean and standard deviation of two independent biological replicates are shown. (TIF) [file pgen.1003820.s009.tif]

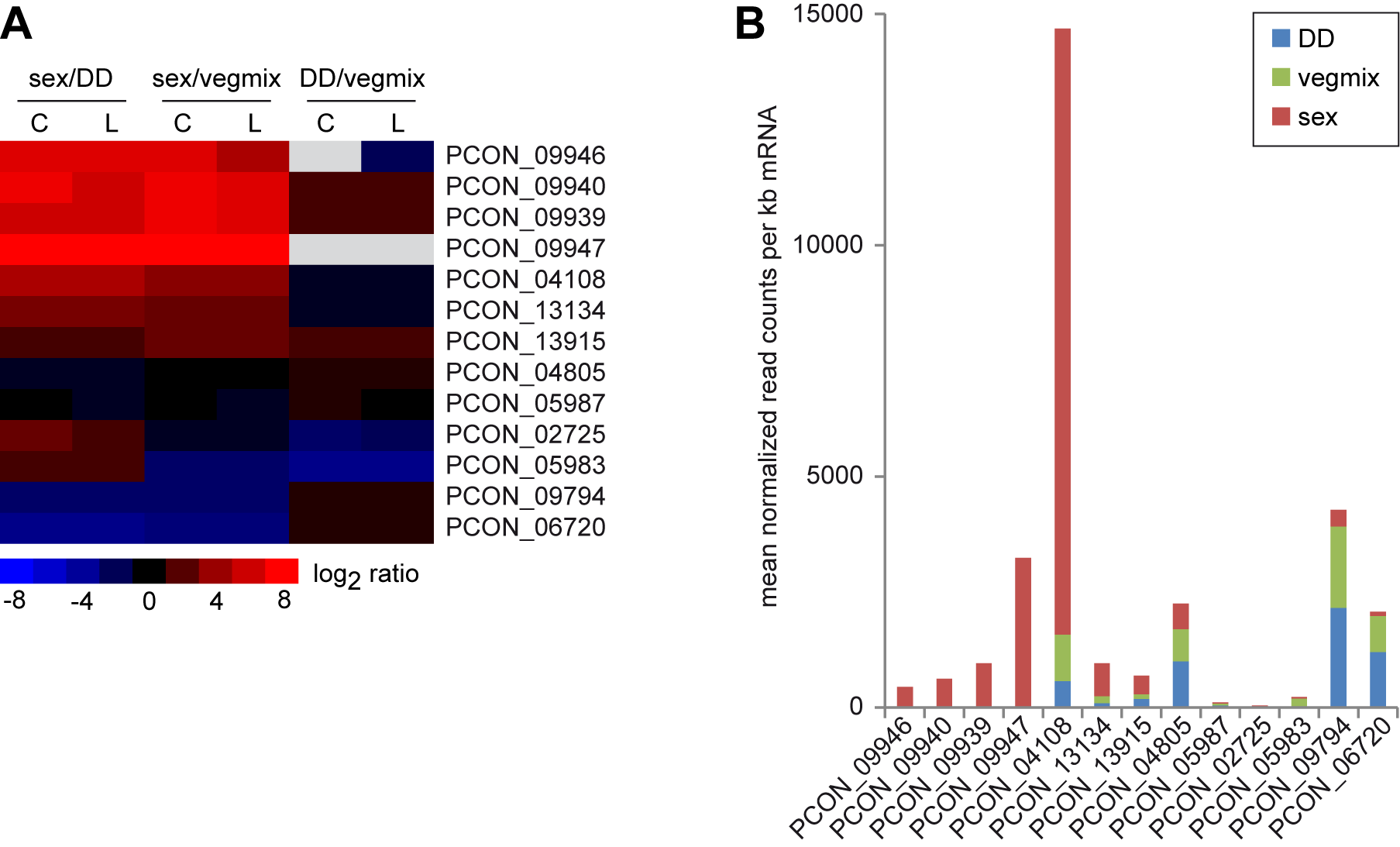

Supplement: Figure S10 — Expression of CBM_14 domain-encoding genes in P. confluens as determined by RNA-seq. A. Expression of CBM_14 domain-encoding proteins during sexual development and vegetative growth. Heatmap of log2 ratios of gene expression under three different conditions. Gray boxes indicate non-determined values. C, ratio from “classical” analysis; L, ratio from LOX analysis. Nearly half of the CBM_14 domain-encoding genes are upregulated during sexual development. B. Overall expression levels vary between CBM_14 domain-encoding genes. Normalized read counts per kb of mRNA (mean of two independent biological replicates) are shown for the three conditions for each gene. Overall read counts per condition vary from none to >13000. (TIF) [file pgen.1003820.s010.tif]

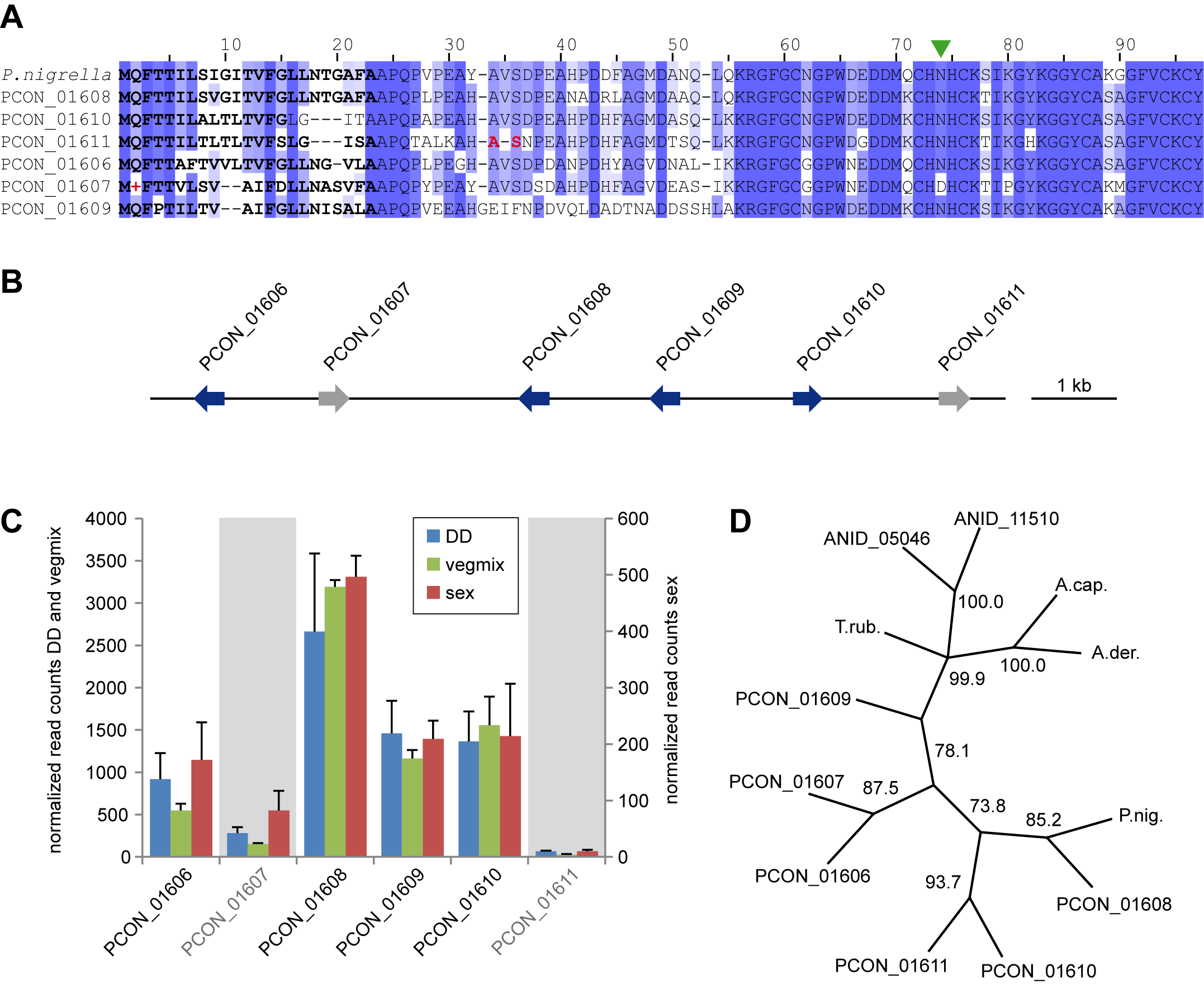

Supplement: Figure S11 — Defensin_2 domain-encoding genes in P. confluens. A. Multiple Alignment of Defensin_2 domain-containing proteins from P. confluens and Pseudoplectania nigrella. Two of the P. confluens genes are pseudogenes that are not expected to yield a functional protein; the stop codon within PCON_01607 and the two amino acids between which a frameshift occurs in PCON_01611 are indicated in red. The predicted signal sequences for co-translational insertion into the ER are given in bold (SignalP 4.0 predictions, Nature Methods 2011, 8:785–786). The position of the conserved intron within the coding region of all P. confluens proteins is indicated by a green triangle above the sequences (the intron is located between the second and third nucleotide of the corresponding nucleotide triplet). Accession number of the P. nigrella plectasin: sp|Q53I06.1. B. Genomic organization of Defensin_2 domain-encoding genes. The genes are located within a region from nt 102001 to nt 113000 of scaffold 1117. The two pseudogenes PCON_01607 and PCON_01611 are shaded in gray. C. Expression of Defensin_2 domain-containing genes. Normalized counts of RNA-seq reads for the three conditions that were investigated (sex, DD, and vegmix) are shown for the six genes; note that the read counts for sexual development are given on the secondary axis to the right, because they are much lower than the read counts in DD and vegmix for all six genes. Read counts for the two pseudogenes PCON_01607 and PCON_01611 (shaded in gray) are generally lower than those for the other genes in all conditions investigated. D. Phylogenetic analysis of Defensin_2 domain-encoding proteins. In addition to the P. confluens proteins, the following proteins were used for Neighbor joining analysis with 1000 bootstrap replications: A.cap, Ajellomyces capsulatus ref|XP_001537899.1; A.der., Ajellomyces dermatitides gb|EEQ83322.1; ANID_05046 and ANID_11510, Aspergillus nidulans from the A. nidulans genome project http://www.broadinstitute.o [file pgen.1003820.s011.tif]

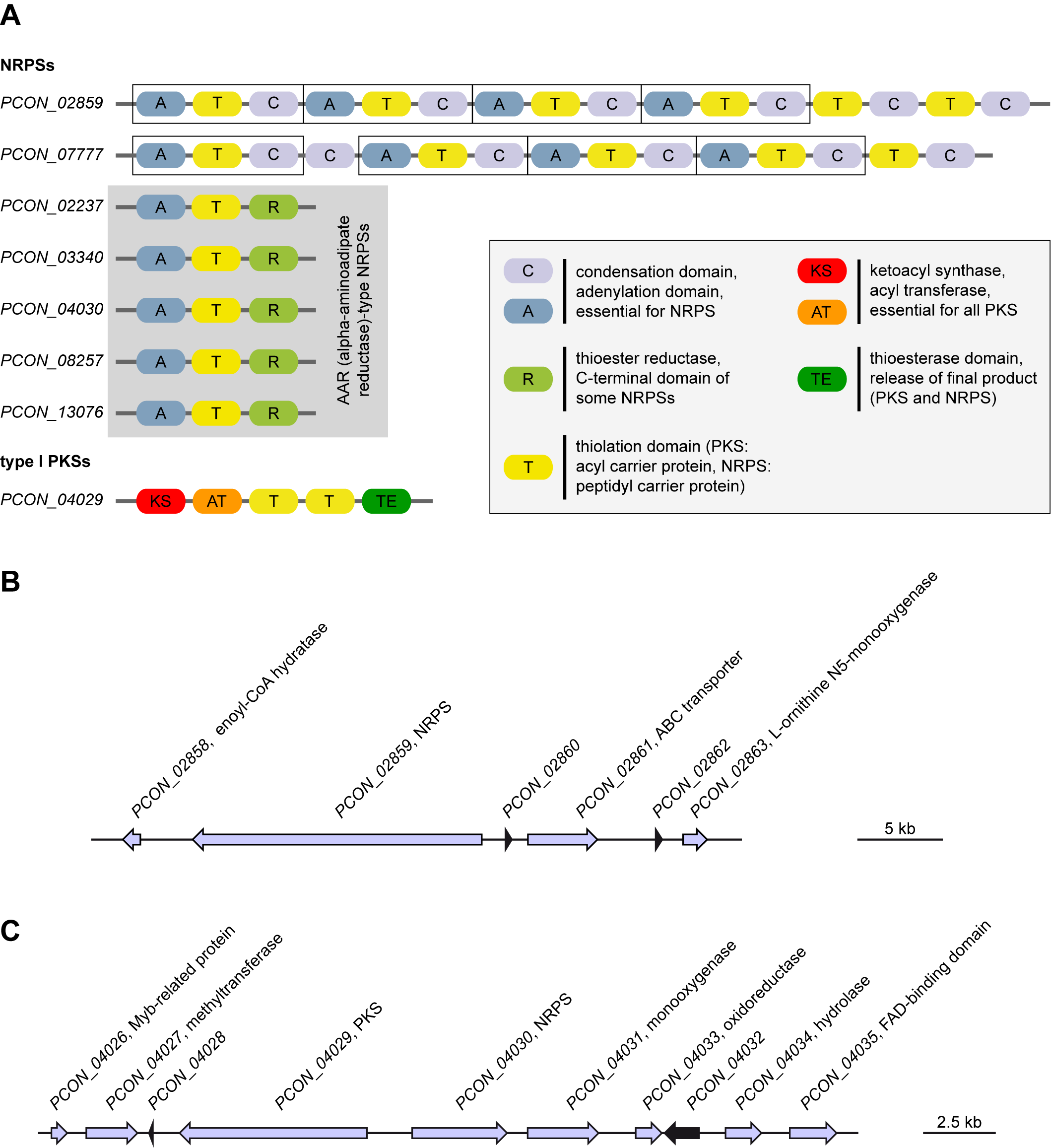

Supplement: Figure S12 — Genes for polyketide and non-ribosomal peptide biosynthesis in P. confluens. A. Protein domain organization of all proteins in P. confluens that are predicted to be PKSs or NRPSs. Complete A-T-C modules in the NRPSs are boxed. The putative siderophore biosynthesis NRPS PCON_02859 has the typical domain structure of four A-T-C modules and two T-C modules. The five AAR-type NRPSs (domain structures A-T-R) are shown in a gray box. B. Putative biosynthetic cluster containing the NRPS gene PCON_02859. Genes with putative functions in siderophore biosynthesis are shown in light blue. C. Putative biosynthetic gene cluster containing the PKS gene PCON_04029 and the NRPS gene PCON_04030. Genes with putative functions in the biosynthesis of a hybrid polyketide/non-ribosomal peptide are shown in light blue. (TIF) [file pgen.1003820.s012.tif]

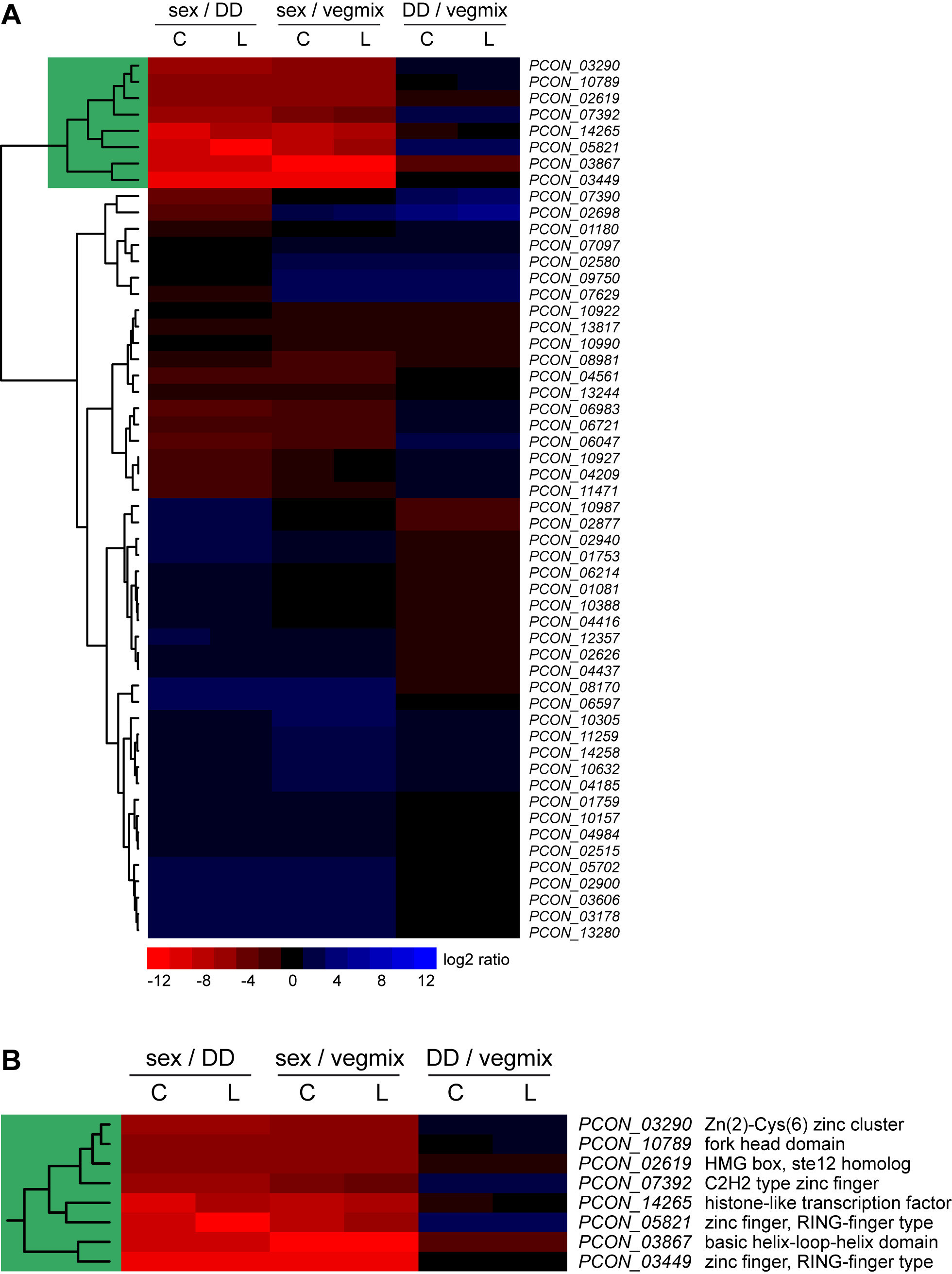

Supplement: Figure S13 — Differentially expressed predicted transcription factors in P. confluens. A. 54 of the 177 predicted transcriptions factors are differentially expressed in at least one comparison (sex/DD, sex/vegmix, DD/vegmix; data from non-stringent analysis); these are shown in this heatmap (clustering and heatmap generation in R). C and L show log2 expression ratios from classical and LOX analysis, respectively. B. Eight putative transcription factors are strongly upregulated (>10×, up to ∼4000×) during sexual development in both sex/DD and sex/vegmix. They contain domains from seven different DNA binding domain families. Only PCON_07392 and PCON_02619 have an ortholog in most investigated fungi, for the others, there are often BLAST hits, but no clear orthologs (in reciprocal BLAST). One reason for this might be that these transcription factors belong to larger families where the definition of orthologs is difficult. PCON_02619 is the ortholog of the S. macrospora/yeast STE12 transcription factor. (TIF) [file pgen.1003820.s013.tif]

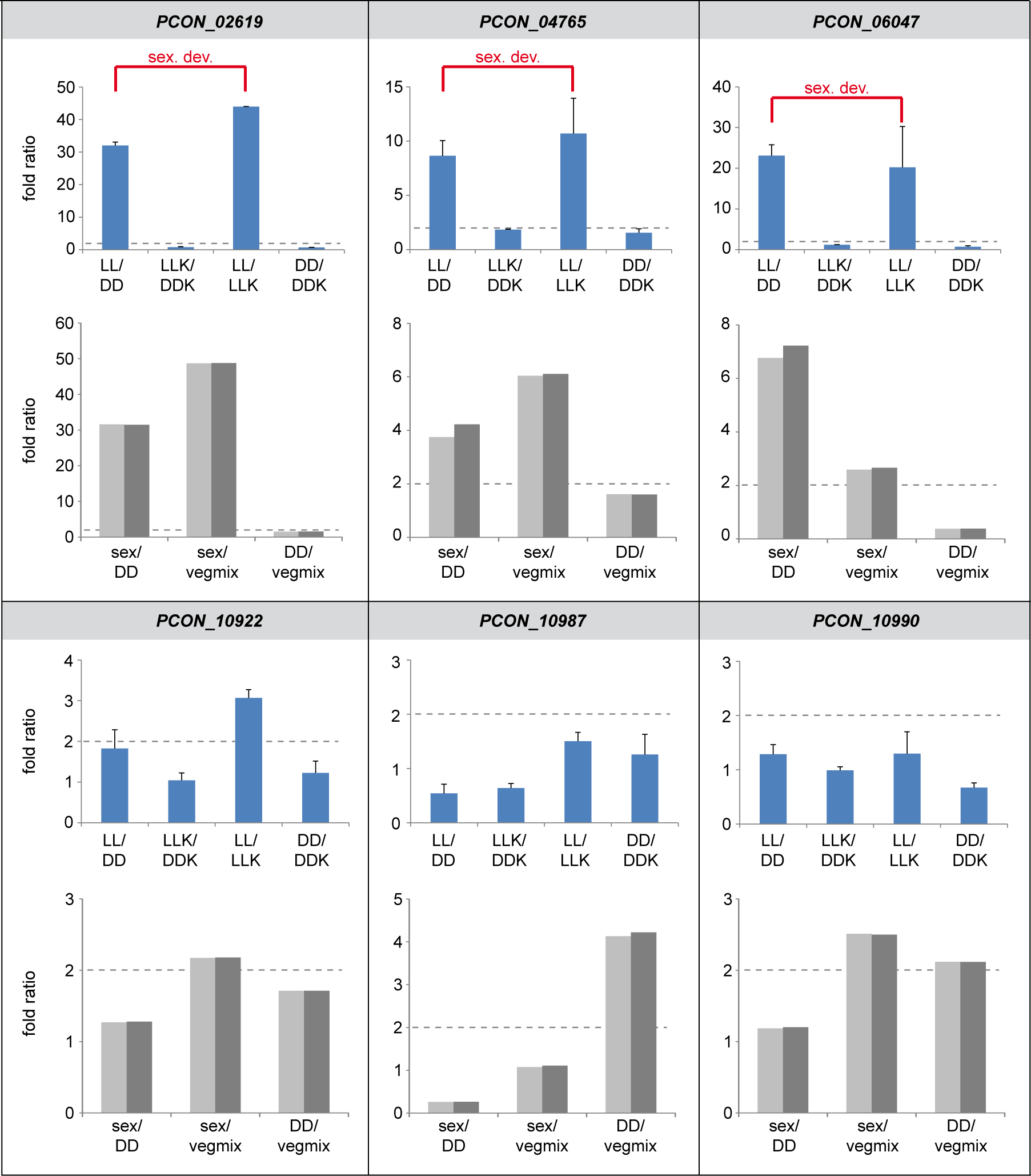

Supplement: Figure S14 — Quantitative real time PCR analysis of selected putative transcription factor genes in P. confluens. Expression was analyzed by qRT-PCR for each gene in two independent biological replicates for the four conditions LL, DD, LLK and DDK (light and darkness in surface culture and submerged culture). Sexual development is only possible in condition LL. Expression ratios are shown as blue bars, and were calculated to address the question if a gene is differentially regulated during sexual development (i.e. in LL/DD and in LL/LLK), or regulated by light (i.e. in LL/DD and LLK/DDK) or regulated by surface versus submerged growth (i.e. LL/LLK and DD/DDK). RNA-seq results for each gene are given as gray bars for comparison: light gray, LOX; dark gray, classic analysis. Dashed lines indicate two-fold upregulation. The qRT-PCR results match the RNA-seq results very well, this can be seen in the comparison sex/DD and LL/DD (same growth conditions, qRT-PCR 3 d, RNA-seq: pool of 3–5 d; the other growth conditions are not directly comparable, but tendencies are conserved). The qRT-PCR data confirm a strong upregulation of PCON_02619, PCON_04765, and PCON_06047 during sexual development only (as opposed to light- or surface culture-dependent, because LL/LLK also gives upregulation which indicates independence of light, and DD/DDK does not give upregulation which indicates that surface culture alone is not sufficient for upregulation). The three genes PCON_10922, PCON_10987, and PCON_10990 are not significantly upregulated by a single condition (sexual development, light, surface culture). PCON_10922 is upregulated in the comparison LL/LLK, but not LL/DD or DD/DDK. This might indicate that surface culture induces the expression of this gene, but only in the light. (TIF) [file pgen.1003820.s014.tif]

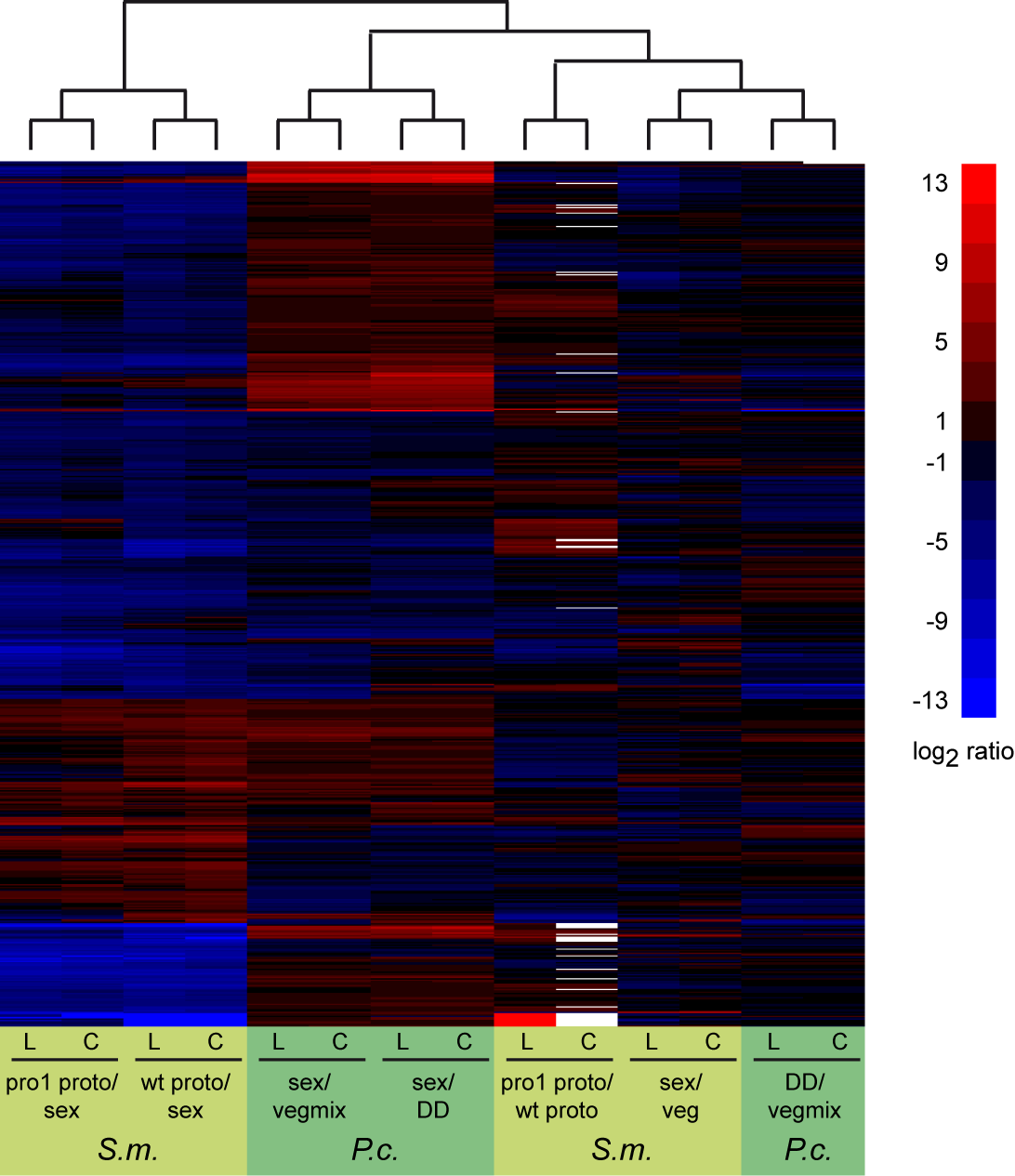

Supplement: Figure S15 — Comparative analysis of gene expression during sexual development in P. confluens (P.c.) and S. macrospora (S.m.). Heat map of hierarchical clustering of log2 expression ratios for comparison of different growth conditions in both fungi. (TIF) [file pgen.1003820.s015.tif]

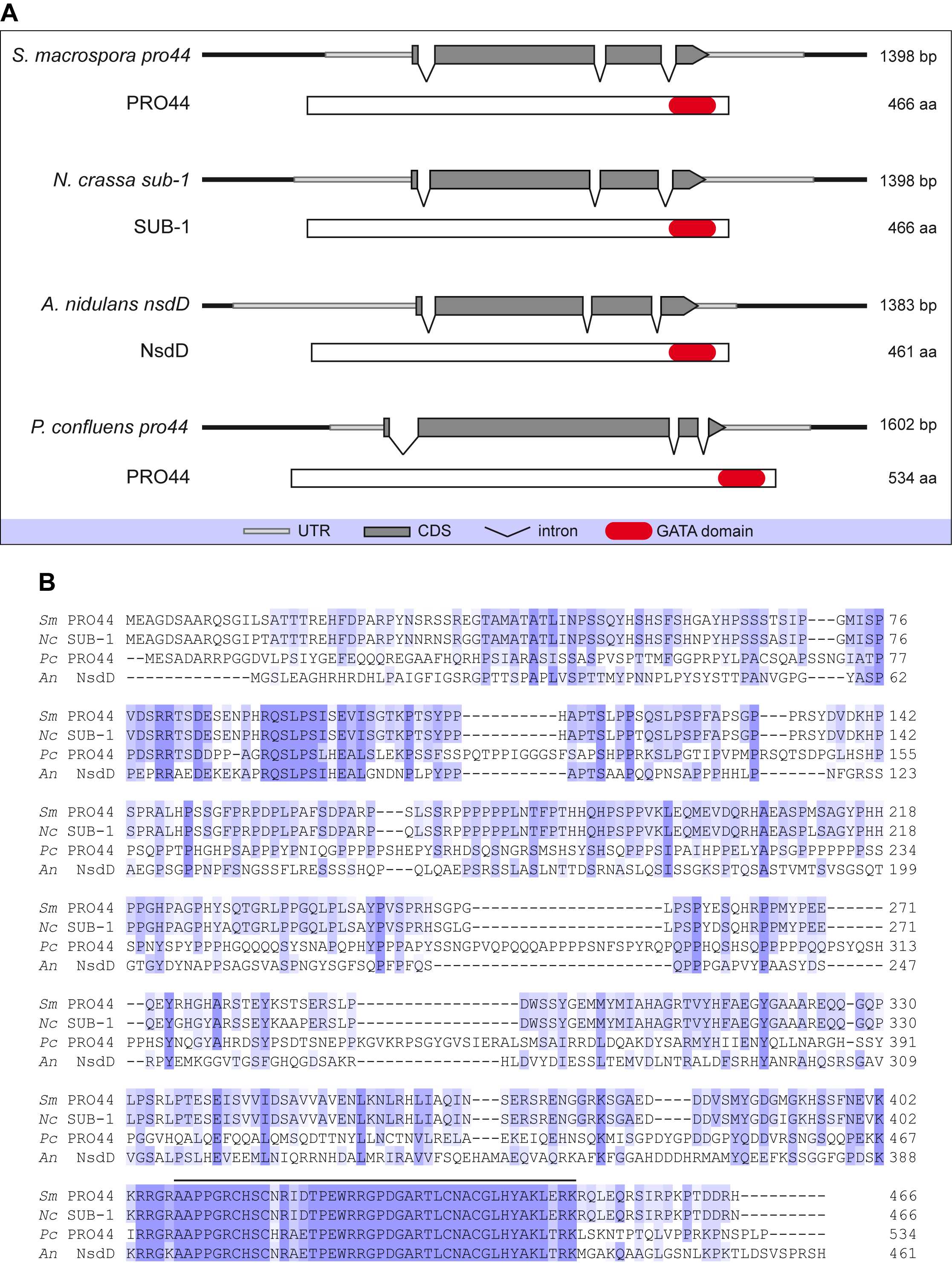

Supplement: Figure S16 — Gene structure and alignments of pro44 homologs. A. Genomic loci and predicted proteins for P. confluens pro44 (PCON_06721) and its orthologs in S. macrospora (pro44, SMAC_03223), N. crassa (sub-1, NCU01154), and A. nidulans (nsdD, ANID_03152). Lengths of coding sequences and proteins are given to the right. B. Multiple alignment of the four proteins. The conserved GATA domain at the C-terminus is indicated by a black line above the sequence. It includes four conserved cysteine residues that bind a zinc atom to form the zinc finger. Data from the following sources: S. macrospora: acc. no. CABT02000007, N. crassa: Neurospora crassa database (http://www.broadinstitute.org/annotation/genome/neurospora/MultiHome.html), A. nidulans: acc. no. U70044 and Aspergillus comparative database (http://www.broadinstitute.org/annotation/genome/aspergillus_group/MultiHome.html). (TIF) [file pgen.1003820.s016.tif]
