# Supplementary material for: The Genome and Development-Dependent Transcriptomes of Pyronema confluens: A Window into Fungal Evolution
Source: PLoS Genet. 2013 Sep 19;9(9):e1003820. doi: 10.1371/journal.pgen.1003820 (PMC3778014; doi:10.1371/journal.pgen.1003820)
Supplement: Table S1 — Overview of DNA and RNA sequencing results for the P. confluens genome and transcriptome. A. Roche/454 sequencing of genomic DNA. B. Illumina/Solexa sequencing of genomic DNA (50 base reads from 300 bp insert paired-end library). C. RNA-seq analysis (Illumina/Solexa sequencing of 101 base reads from 300 bp insert paired-end cDNA library). (PDF) [file pgen.1003820.s017.pdf]

Table S1. Overview of DNA and RNA sequencing results for the *P. confluens* genome and transcriptome

A. Roche/454 sequencing of genomic DNA

no. of reads in raw data	1,941,228
no. of reads after quality trimming	1,772,000
total length of trimmed reads	632 Mb
average length of trimmed reads	357 bases

B. Illumina/Solexa sequencing of genomic DNA (50 base reads from 300 bp insert paired-end library)

no. of reads in raw data	233,571,340
read pairs after quality trimming	205,166,750
single reads after quality trimming	12,944,813
total length of trimmed reads	10,840 Mb

C. RNA-seq analysis (Illumina/Solexa sequencing of 101 base reads from 300 bp insert paired-end cDNA library)

**condition**	**independent biol. replicate**	**no. of cleaned reads**	**no. of mapped reads**	**% of reads that map**
sex	sex1	89,421,077	75,927,569	84.9
	sex2	98,872,105	90,479,204	91.5
DD	DD1	71,994,183	62,756,746	87.2
	DD2	72,126,216	66,123,948	91.7
vegmix	vegmix1	92,177,140	85,152,565	92.4
	vegmix2	71,819,312	64,342,583	89.6
