# Supplementary material for: The Genome and Development-Dependent Transcriptomes of Pyronema confluens: A Window into Fungal Evolution
Source: PLoS Genet. 2013 Sep 19;9(9):e1003820. doi: 10.1371/journal.pgen.1003820 (PMC3778014; doi:10.1371/journal.pgen.1003820)
Supplement: Table S5 — P. confluens homologs of chromatin-associated proteins and proteins involved in genome defense. A. Histones and histone modification. B. DNA methylation machinery. C. RNA interference and meiotic silencing. (PDF) [file pgen.1003820.s021.pdf]

Table S5. ***P. confluens*** **homologs of chromatin-associated proteins and proteins involved in genome defense.**

A. Histones and histone modification. *P. confluens* encodes a typical eukaryotic set of histones with one gene each for histones H1, H2AZ, H2A, H2B, H3, and H4-1, and a separate H4-2. H3/H4-1 and H2A/H2B occur in divergently transcribed clusters. In addition, there is another H4 homolog (H4?) that is not part of the usual histone gene set, but also occurs in other filamentous fungi, e.g. in *N. crassa* (Microbiol. Mol. Biol. Rev., 2004, 68: 1-108). Whether this gene has a specific function in fungi remains to be elucidated.

The histone modification machinery is similar to that in other filamentous fungi (data not shown). Interestingly, *P. confluens* encodes the catalytic subunit (PCON_07755) of the PRC2 complex responsible for H3K27 trimethylation and gene silencing in *N. crassa* (Proc. Natl. Acad. Sci. USA, 2013, in press); and different from *T. melanosporum*, where the gene for the catalytic subunit is missing. This might have led to less gene silencing in *T. melanosporum*, and could be an explanation for the transposon invasion in this species.

locus tag	description/putative function	acc. closest homolog*
PCON_05919	Histone H1	Q9UV33
PCON_13456	Histone H2A.Z	A1C5F1
PCON_05921	Histone H2A	Q875B8
PCON_05922	Histone H2B	Q0CBD1
PCON_13209	Histone H3	P23753
PCON_13210	Histone H4-1	P23750
PCON_05222	Histone H4-2	P23750
PCON_07103	Histone H4?	P23751
PCON_10853	CenH3	Q9Y812

*Swissprot or nr (GenBank)

B. DNA methylation machinery. *P. confluens* has four putative DNA methyltransferases, two of which are homologous to DIM2 (de novo cytosine methylation), one to RID (involved in RIP [repeat induced point mutation] in *N. crassa*, Proc. Natl. Acad. Sci USA, 2002, 99:8802-8807), and one is an unusual fusion of a DNA methyltransferase domain with an Rad8/Rad5 DNA repair protein domain. Most ascomycetes harbor only one DIM2-type protein; however, two DIM2 proteins have, for example, been found in *Coprinopsis cinerea*.

locus tag	description/putative function	acc. closest homolog*
PCON_01959	cytosine DNA methyltransferase DIM2-1	Q24K09
PCON_02009	cytosine DNA methyltransferase DIM2-2	Q24K09
PCON_06255	cytosine DNA methyltransferase RID	P52311
PCON_08358	DNA methylase domain fused with Rad8/Rad5 homolog	Q6C2R8

*Swissprot or nr (GenBank)

C. RNA interference and meiotic silencing. The complement of putative RNA interference genes in *P. confluens* is similar to other fungi, with a slight expansion in some gene families. For example, there are four Argonaut proteins instead of the more usual two that are present in *N. crassa* and also in *T. melanosporum*.

locus tag	description/putative function	evalue*
PCON_01366	RNA-dependent RNA polymerase (similar to QDE-1)	2e-75
PCON_13920	RNA-dependent RNA polymerase (similar to QDE-1)	4e-48
PCON_05873	RNA-dependent RNA polymerase (similar to QDE-1)	3e-12
PCON_09961	Argonaut (similar to QDE-2, best match in *T. melanosporum* acc. no. CAZ85927)	3e-96
PCON_04155	Argonaut (similar to QDE-2, best match in *T. melanosporum* acc. no. CAZ85927)	3e-93
PCON_11553	Argonaut (similar to QDE-2, best match in *T. melanosporum* acc. no. CAZ85927)	1e-76
PCON_02188	Argonaut (similar to SMS-2, involved in meiotic silencing, best match in *T. melanosporum* acc. no. CAZ83738)	7e-54
PCON_01517	Dicer	9e-121
PCON_03869	Dicer	3e-31
PCON_01652	RecQ helicase (similar to QDE-3)	2e-153

*in BLAST searches with the corresponding *N. crassa* genes
