# Supplementary material for: The Genome and Development-Dependent Transcriptomes of Pyronema confluens: A Window into Fungal Evolution
Source: PLoS Genet. 2013 Sep 19;9(9):e1003820. doi: 10.1371/journal.pgen.1003820 (PMC3778014; doi:10.1371/journal.pgen.1003820)
Supplement: Table S7 — Mating type, pheromone, pheromone receptor, pheromone-processing, and signal transduction genes, and homologs to developmental genes in P. confluens. A. Genes within or adjacent to the mating type loci. Genes that are upregulated at least two-fold in both classic (C) and LOX (L) analysis in the conditions investigated are given in bold red. B. Putative pheromone processing proteins. Genes that are upregulated at least two-fold in both classic (C) and LOX (L) analysis in the conditions investigated are given in bold red. C. Putative proteins of the pheromone response pathway. Genes that are upregulated at least two-fold in both classic (C) and LOX (L) analysis in the conditions investigated are given in bold red. D. Putative MAP kinase modules. E. P. confluens homologs to developmental proteins from S. macrospora. (PDF) [file pgen.1003820.s023.pdf]

Table S7. Mating type, pheromone, pheromone receptor, pheromone-processing, and signal transduction genes, and homologs to developmental genes in in *P. confluens*.

A. Genes within or adjacent to the mating type loci. Genes that are upregulated at least two-fold in both classic (C) and LOX (L) analysis in the conditions investigated are given in bold red.

locus tag	peptide lengt in amino acids	Position within mating type locus	best BLASTP hit	closest homolog in *T. melanosporum*	putative function	sex/DD		sex/vegmix		DD/vegmix	
						C	L	C	L	C	L
PCON_07490	1102	Left flank of MAT1-1	[AN4791.2] uncharacterized protein, *Emericella nidulans*	GSTUMT00008232001	unknown	0.83	0.82	1.16	1.13	1.41	1.38
PCON_07491	340	MAT1-1-1	[ADU56595.1] Mating type protein MAT1-1-1, *Tuber melanosporum*	no hit	alpha domain mating type transcription factor	**4.70**	**4.60**	**5.67**	**5.60**	1.22	1.22
PCON_07492	48	In the mating type locus or right flank of MAT1-1	no hit	no hit	unknown	65.00	n.d.	65.00	n.d.	n.d.	n.d.
PCON_07493	384	Right flank of MAT1-1	[CAZ84152] uncharacterized protein, *Tuber melanosporum*	GSTUMT00008221001	unknown	0.54	0.54	0.66	0.66	1.22	1.23
PCON_08385	527	Left flank of MAT1-2	[GAA85719.1] DNA lyase Apn2, *Aspergillus kawachii*	no hit	APN2 DNA lyase	0.94	0.93	1.42	1.39	1.51	1.5
PCON_08388	341	Left flank of MAT1-2	[EED49897.1] putative phospholipase, *Aspergillus flavus*	GSTUMT00006682001	phosphoplipase	0.84	0.84	**2.17**	**2.00**	**2.59**	**2.38**

PCON_08389	274	MAT1-2-1	[B4E458] Mating type protein mat-2-1, *Gibberella indica*	GSTUMT00001090001	HMG domain mating type transcription factor	1.43	1.4	1.25	1.23	0.88	0.88
PCON_08390	124	In the mating-type locus or right flank of MAT1-2	no hit	no hit	unknown	**14.93**	**7.33**	**10.16**	**8.22**	1.38	1.12
PCON_08391	1095	Right flank of MAT1-2	[EPG82700] uncharacterized protein MYCGRDRAFT_111677, *Mycosphaerella graminicola*	GSTUMT00008232001	unknown	1.45	1.43	1.75	1.65	1.2	1.16

B. Putative pheromone processing proteins. Genes that are upregulated at least two-fold in both classic (C) and LOX (L) analysis in the conditions investigated are given in bold red.

S. cerevisiae protein	function	E-value (blastp vs. *P. confluens*)	*P. confluens* locus tag	best BLASTP hit (*P. confluens* vs. GenBank)	sex/DD		sex/vegmix		DD/vegmix	
					C	L	C	L	C	L
Kex1p	carboxy-peptidase	6e-090	PCON_03828	[XP_003070320.1] putative pheromone processing carboxypeptidase, *Coccidioides posadasii*	0.96	0.97	0.86	0.86	0.9	0.89
Kex2p	endopeptidase	e-158	PCON_13110	[XP_001593105] hypothetical protein SS1G_06027, *Sclerotinia sclerotiorum*	**2.42**	**2.42**	**2.85**	**2.82**	1.18	1.17
Ste13p	dipeptidyl aminopeptidase	5e-086	PCON_01634	[EEH18802.1] dipeptidyl aminopeptidase B *Paracoccidioides brasiliensis*	0.93	0.93	0.58	0.56	0.62	0.6
Ste24p	CAAX prenyl protease	e-111	PCON_13836	[EGX50261.1] hypothetical protein AOL_s00076g226, *Arthrobotrys oligospora*	1.41	1.39	1.34	1.29	0.95	0.93
Rce1p	CAAX prenyl protease 1	6e-016	PCON_11402	[EED51246.1] CaaX prenyl proteinase Rce1, *Aspergillus flavus*	1.10	1.08	1.24	1.23	1.13	1.14

Ste23p	Secreted/periplasmic Zn-dependent peptidase	0.0	PCON_05396	[XP_681313.1] hypothetical protein AN8044.2, *Aspergillus nidulans*	**5.06**	**5.13**	**4.69**	**4.72**	0.93	0.92
Ram1p	Prenyl-transferase, beta subunit	2e-066	PCON_08707	[XP_003066451.1] putative protein farnesyltransferase beta subunit, *Coccidioides posadasii*	1.65	1.67	1.29	1.31	0.78	0.79
Ram2p	Protein prenyltransferase, alpha subunit	1e-051	PCON_13838	[EGP85616.1] hypothetical protein MYCGRDRAFT_73812, *Mycosphaerella graminicola*	1.53	1.54	1.04	1.05	0.68	0.68
Ste14p	Farnesyl cysteine-carboxyl methyltransferase	9e-050	PCON_04672	[XP_001267767.1] prenyl cysteine carboxyl methyltransferase Ste14, *Aspergillus clavatus*	1.16	1.15	1.20	1.21	1.05	1.06
Ste6p	ABC-type multidrug transport system, pheromone efflux pump	1e-128	PCON_02811	[XP_002562150.1] Pc18g03100 *Penicillium chrysogenum*	0.75	0.72	1.19	1.19	1.66	1.66

C. Putative proteins of the pheromone response pathway. Genes that are upregulated at least two-fold in both classic (C) and LOX (L) analysis in the conditions investigated are given in bold red.

S. cerevisiae protein	function	E-value (blastp vs. *P. confluens*)	*P. confluens* locus tag	best BLASTP hit (*P. confluens* vs. GenBank)	sex/DD		sex/vegmix		DD/vegmix	
					C	L	C	L	C	L
Ste2p	peptide receptor	3e-012	PCON_02097	[EGX50494.1] hypothetical protein AOL_s00075g223, *Arthrobotrys oligospora*	**122.41**	**28.88**	**76.17**	**56.29**	2.33	1.95
Ste3p	lipopeptide receptor	2e-031	PCON_08875	[XP_002544713.1] predicted protein, *Uncinocarpus reesii*	**32.14**	**31.76**	**56.53**	**50.43**	1.76	1.59
Gpa1p	alpha subunit G-protein	2e-072	PCON_07314	[XP_001800368.1] G-alpha subunit, *Phaeosphaeria nodorum*	1.09	1.05	1.19	1.17	1.11	1.12
		7e-066	PCON_07308	[EGX50588.1] hypothetical protein AOL_s00075g14, *Arthrobotrys oligospora*	1.07	1.07	1.45	1.43	1.36	1.34
		3e-055	PCON_04877	[EGX46447.1] hypothetical protein AOL_s00109g19, *Arthrobotrys oligospora*	1.05	1.02	1.25	1.24	1.21	1.22

Ste4p	beta subunit G-protein	2e-077	PCON_14045	[EGX53779.1] hypothetical protein AOL_s00004g438, *Arthrobotrys oligospora*	0.74	0.72	0.89	0.88	1.22	1.21
Ste18p	gamma subunit G-protein	5e-009	PCON_01676	[EEA21555.1] G-protein complex gamma subunit GpgA, *Penicillium marneffei*	0.64	0.63	0.73	0.73	1.15	1.15
Sst2p	RGS protein	e-023	PCON_02904	[EGX43780.1] hypothetical protein AOL_s00215g516, *Arthrobotrys oligospora*	0.75	0.74	0.93	0.93	1.26	1.27
Cdc24p	Guanine nucleotide exchange factor for Rho/Rac/Cdc42-like GTPases	7e-050	PCON_08710	[EGX49557.1] hypothetical protein AOL_s00078g46, *Arthrobotrys oligospora*	1.14	1.1	1.29	1.24	1.15	1.13
Cdc42p	Small rho-like GTPase	3e-094	PCON_10718	[EGX52049.1] hypothetical protein AOL_s00043g439, *Arthrobotrys oligospora*	0.91	0.89	0.86	0.84	0.95	0.93
Bem1p	scaffold protein for complexes that include Cdc24p, Ste5p, Ste20p, and Rsr1p	2e-057	PCON_09345	[EED18295.1] putative protein kinase activator Bem1, *Talaromyces stipitatus*	0.75	0.75	0.88	0.88	1.17	1.17

Ste50p	adaptor linking Cdc42p-Ste20p complex to the effector Ste11p to modulate signal transduction	9e-17	PCON_08369	[XP_756047.1] MAPKKK cascade protein kinase regulator Ste50, *Aspergillus fumigatus*	1.30	1.28	1.22	1.17	0.95	0.91
Ste20p	PAK (p21-activated kinase)	e-113	PCON_11179	[EED23353.1] sexual development serine/threonine kinase PakA, *Talaromyces stipitatus*	1.04	1.03	1.06	1.02	1.03	0.99
Ste5p	scaffold of MAP kinase cascade	--	no hit	--	--	--	--	--	--	--
Ste11p	MEK kinase	1e-086	PCON_03982	[XP_962994.2] hypothetical protein NCU06182, *Neurospora crassa*	0.87	0.86	1.09	1.05	1.28	1.22
Ste7p	MAP kinase kinase	5e-065	PCON_02257	[XP_001263859.1] MAP kinase kinase Ste7, *Neosartorya fischeri*	0.83	0.82	0.76	0.75	0.91	0.91
Fus3p	MAP kinase	e-125	PCON_05927	[EGX46330.1] hypothetical protein AOL_s00110g154, *Arthrobotrys oligospora*	0.97	0.97	0.99	0.95	1.02	0.99
Kss1p	MAP kinase	e-123	PCON_05927	[EGX46330.1] hypothetical protein AOL_s00110g154,	0.97	0.97	0.99	0.95	1.02	0.99

				Arthrobotrys oligospora						
Ste12p	homeobox transcription factor	8e-062	PCON_08877	[EFY93360.1] transcription factor Fst12, *Metarhizium acridum*	0.71	0.70	0.94	0.92	1.34	1.32
Dig1p	Regulatory protein required for MAP-kinase imposed repression	--	no hit	--	--	--	--	--	--	--
Dig2p	Regulatory protein required for MAP-kinase imposed repression	--	no hit	--	--	--	--	--	--	--

D. Putative MAP kinase modules.

P. confluens	*S. macrospora*		*N. crassa*		protein	function
locus tag	locus tag	acc. no.	locus tag	acc. no.		
PCON_03027	SMAC_06775	ref|XP_003351367.1	NCU02234	gb|EAA30411.2	MIK1	MAPKKK
PCON_05319	SMAC_07802	ref|XP_003346461.1	NCU06182	gb|EAA33758.2	MIK2	MAPKKK
PCON_07314	SMAC_05328	ref|XP_003348304.1	NCU03071	gb|EAA34928.1	OS4	MAPKKK
PCON_07308	SMAC_06605	ref|XP_003350470.1	NCU06419	gb|EAA28074.2	MEK1	MAPKK
PCON_04877	SMAC_07195	ref|XP_003345972.1	NCU04612	gb|EAA29619.1	MEK2	MAPKK
PCON_08682	SMAC_01638	ref|XP_003352445.1	NCU00587	gb|EAA36491.2	OS5	MAPKK
PCON_11743	SMAC_00338	ref|XP_003349221.1	NCU09842	gb|EAA28804.1	MAK1	MAPK
PCON_13421	SMAC_08794	ref|XP_003351189.1	NCU02393	gb|EAA30477.2	MAK2	MAPK
PCON_12605	SMAC_02580	ref|XP_003343998.1	NCU07024	sp|Q96TL5.1	OS2	MAPK

E. *P. confluens* homologs to developmental proteins from *S. macrospora*.

P. confluens		*S. macrospora*		*N. crassa*		protein	function
locus tag	evalue	locus tag	acc. no.	locs tag	acc. no.		
PCON_03027	0.0	SMAC_06775	ref|XP_003344998.1	NCU06785	sp|Q8X097.1	ACL1	subunit of ATP citrate lyase
PCON_05319	3E-117	SMAC_07802	ref|XP_003352361.1	NCU03564	gb|EAA26891.2	LEU1	β-Isopropylmalate dehydrogenase
PCON_07314	0.0	SMAC_05328	ref|XP_003346433.1	NCU06493	sp|Q05425.1	GSA1	G-protein α-subunit
PCON_07308	4E-137	SMAC_06605	ref|XP_003346138.1	NCU06729	sp|Q05424.1	GSA2	G-protein α-subunit
PCON_04877	1E-154	SMAC_07195	ref|XP_003347338.1	NCU05206	sp|Q9HFW7.1	GSA3	G-protein α-subunit
PCON_08682	0.0	SMAC_01638	ref|XP_003352805.1	NCU08377	sp|Q01631.2	SAC1	Adenylate cyclase
PCON_11743	2E-157	SMAC_00338	ref|XP_003351793.1	NCU07392	sp|Q9P326.1	PRO1	C6 zinc finger transcription factor
PCON_13421	1E-96	SMAC_08794	ref|XP_003345440.1	NCU08741	gb|EAA34366.1	PRO11	WD40-repeat protein
PCON_12605	7E-138	SMAC_02580	ref|XP_003352145.1	NCU03727	gb|EAA32267.2	PRO22	membrane protein
PCON_02536	0.0	SMAC_04815	ref|XP_003347511.1	NCU02794	gb|EAA34840.2	PRO40	WW domain protein
PCON_03268	6E-50	SMAC_04848	ref|XP_003347542.1	NCU02767	gb|EAA35026.1	PRO41	membran protein
PCON_06721	5E-24	SMAC_03223	ref|XP_003349634.1	NCU01154	gb|EAA31340.1	PRO44	Zinc finger; GATA transcription factor
PCON_14305	3E-74	SMAC_08608	ref|XP_003345657.1	NCU09436	sp|Q7S1X9.1	ASF1	histone chaperone, anti silencing function
