# Supplementary material for: The Genome and Development-Dependent Transcriptomes of Pyronema confluens: A Window into Fungal Evolution
Source: PLoS Genet. 2013 Sep 19;9(9):e1003820. doi: 10.1371/journal.pgen.1003820 (PMC3778014; doi:10.1371/journal.pgen.1003820)
Supplement: Table S10 — Gene expression under different light regimes. Transcript levels were determined after short term light induction (5–60 min after growth in darkness for 4 d). P. confluens was grown in minimal liquid medium and harvested under far-red light. Transcript levels were determined by qRT-PCR from at least two independent biological replicates, ratios versus 4 d in darkness (DD) and standard deviations are given. (PDF) [file pgen.1003820.s026.pdf]

**Table S10.** Gene expression under different light regimes. Transcript levels were determined after short term light induction (5-60 min after growth in darkness for 4 d). *P. confluens* was grown in minimal liquid medium and harvested under far-red light. Transcript levels were determined by qRT-PCR from at least two independent biological replicates, ratios versus 4 d in darkness (DD) and standard deviations are given. Expression of the cryptochrome gene *cry* was tested, but no expression was detected in the conditions investigated.

| | | *wc1* | | *wc2* | | *frq* | | *pro44* | | *orp* | | *phy1* | | *phy2* | | *al1* | | *al2* | | *al3* | |
|---|---|---|---|---|---|---|---|---|---|---|---|---|---|---|---|---|---|---|---|---|---|
| | | ratio | +/- | ratio | +/- | ratio | +/- | ratio | +/- | ratio | +/- | ratio | +/- | ratio | +/- | ratio | +/- | ratio | +/- | ratio | +/- |
| **white light** | DD | 1 | 0 | 1 | 0 | 1 | 0 | 1 | 0 | 1 | 0 | 1 | 0 | 1 | 0 | 1 | 0 | 1 | 0 | 1 | 0 |
| | 5LL | 1.11 | 0.15 | 0.95 | 0.11 | 2.52 | 0.31 | 3.09 | 0.53 | 1.90 | 0.03 | 1.01 | 0.32 | 1.23 | 0.36 | 2.4 | 0.51 | 3.28 | 0.24 | 3.32 | 0.04 |
| | 10LL | 1.23 | 0.09 | 0.89 | 0.04 | 6.13 | 0.28 | 4.96 | 0.37 | 2.30 | 0.82 | 1.01 | 0.21 | 2.00 | 0.34 | 7.08 | 5.36 | 11.50 | 0.73 | 10.20 | 2.54 |
| | 30LL | 1.73 | 0.21 | 1.18 | 0.02 | 10.8 | 0.27 | 7.98 | 1.88 | 2.41 | 0.26 | 1.17 | 0.36 | 2.51 | 1.00 | 136.00 | 13.40 | 247.00 | 164.00 | 40.30 | 22.80 |
| | 60LL | 1.66 | 0.82 | 1.41 | 0.29 | 8.61 | 3.29 | 8.35 | 2.65 | 2.02 | 0.85 | 1.19 | 0.2 | 3.29 | 1.45 | 594.00 | 108.00 | 1838.00 | 1318.00 | 74.80 | 13.20 |
| **blue light** | DD | 1 | 0 | 1 | 0 | 1 | 0 | 1 | 0 | 1 | 0 | 1 | 0 | 1 | 0 | 1 | 0 | 1 | 0 | 1 | 0 |
| | 5LL | 0.81 | 0.06 | 0.74 | 0.05 | 1.48 | 0.33 | 1.57 | 0.45 | 0.71 | 0.48 | 0.83 | 0.02 | 1.06 | 0.10 | 1.11 | 0.03 | 1.63 | 0.61 | 1.86 | 1.15 |
| | 10LL | 1.21 | 0.12 | 0.87 | 0.16 | 4.17 | 2.86 | 3.87 | 2.61 | 0.89 | 0.26 | 1.1 | 0.03 | 2.01 | 1.02 | 4.87 | 1.57 | 11.90 | 11.02 | 6.34 | 6.55 |
| **green light** | DD | 1 | 0 | 1 | 0 | 1 | 0 | 1 | 0 | 1 | 0 | 1 | 0 | 1 | 0 | 1 | 0 | 1 | 0 | 1 | 0 |
| | 5LL | 1.19 | 0.34 | 1.15 | 0.3 | 1.72 | 0.33 | 1.93 | 0.41 | 1.57 | 0.82 | 1.31 | 0.61 | 1.23 | 0.35 | 1.12 | 0.27 | 1.67 | 0.10 | 1.26 | 0.37 |
| | 10LL | 1.13 | 0.38 | 1.26 | 0.43 | 2.32 | 0.82 | 3.43 | 1.41 | 1.50 | 0.81 | 1.56 | 0.89 | 1.63 | 0.72 | 1.68 | 0.96 | 3.97 | 3.04 | 1.80 | 0.46 |
