# Supplementary material for: The Genome and Development-Dependent Transcriptomes of Pyronema confluens: A Window into Fungal Evolution
Source: PLoS Genet. 2013 Sep 19;9(9):e1003820. doi: 10.1371/journal.pgen.1003820 (PMC3778014; doi:10.1371/journal.pgen.1003820)
Supplement: Table S12 — P. confluens homologs to heterokaryon incompatibility genes from N. crassa and P. anserina. For N. crassa and P. anserina proteins shaded in gray, no clear P. confluens ortholog can be identified. A. N. crassa het genes. B. P. anserina het genes. (PDF) [file pgen.1003820.s028.pdf]

Table S12. *P. confluens* homologs to heterokaryon incompatibility genes from *N. crassa* and *P. anserina*. For *N. crassa* and *P. anserina* proteins shaded in gray, no clear *P. confluens* ortholog can be identified.

A. *N. crassa het* genes

Gene	Ref.	Characteristics	homologs in *P. confluens*	e-value BastP
mat A1 (NCU01958)	[1]	mating-type gene (alpha domain)	No hit, but mating-type protein PCON_07491 homologous to MATA1	-
mat a1	[2]	mating-type gene (HMG box)	PCON_08389	1e-15
het-c (NCU03493)	[3]	allelic *het* gene, signal peptide, transmembrane domain, glycine rich	PCON_05333	e-171
pin-c1 (NCU03494)	[4]	non-allelic *het* gene, TOL/HET-6/HET-E domain, interaction with *het-c*	PCON_01604<br>PCON_09721<br>PCON_02361<br>PCON_02350	4e-040<br>1e-026<br>1e-025<br>4e-021
het-6 (NCU03533)	[5]	allelic het gene, TOL/HET-6/HET-E domain	PCON_05306<br>PCON_02361<br>PCON_09721<br>PCON_02350	2e-007<br>1e-006<br>8e-005<br>7e-004
un-24 (NCU03539)	[5]	allelic *het* gene, ribonucleoside-diphosphate reductase large chain	PCON_07770	0.0
tol (NCU04453)	[6]	Suppressor of incompatibility TOL/HET-6/HET-E domain	PCON_01604<br>PCON_02024<br>PCON_09721<br>PCON_02361<br>PCON_02350<br>PCON_12913<br>PCON_03497	3e-041<br>3e-022<br>1e-019<br>4e-019<br>3e-017<br>6e-015<br>2e-007
vib-1 (NCU03725)	[7]	Suppressor of incompatibility, nuclear localization domain	PCON_08807 (reciprocal best hit is NCU09915)	5e-011

B. *P. anserina het* genes

Gene	Ref.	Characteristics	homologs in *P. confluens*	e-value BastP
het-c2 AAA20542.1	[8]	non-allellic het gene against het-d and het-e, glycolipid transfer protein	PCON_10723	3e-056
het-d CAL30216.1	[9]	non-allelic *het* gene against *het-c*, WD repeat doamin, TOL/HET-6/HET-E domain, GTP-binding	PCON_11390<br>PCON_04786<br>PCON_11858<br>PCON_12888	1e-049<br>3e-039<br>1e-034<br>4e-033

			PCON_10803<br>PCON_09822<br>PCON_01716<br>PCON_10843<br>PCON_14293<br>PCON_03032<br>PCON_14268<br>PCON_13460<br>PCON_02131<br>PCON_08266<br>PCON_07564<br>PCON_05306<br>PCON_03101	7e-031<br>2e-027<br>5e-025<br>7e-025<br>2e-015<br>1e-014<br>4e-011<br>1e-010<br>2e-010<br>4e-008<br>2e-006<br>9e-004<br>0.068
het-e<br>AAL37300	[9]	non-allelic *het* gene against *het-c*, WD repeat doamin, TOL/HET-6/HET-E domain, GTP-binding	PCON_11390<br>PCON_04786<br>PCON_12888 (only 138 aa)<br>PCON_10803 (only 154 aa)<br>PCON_11858<br>PCON_09822 (only 217 aa)<br>PCON_01716<br>PCON_10843<br>PCON_14293<br>PCON_03032<br>PCON_05422 (only 173 aa)<br>PCON_14268<br>PCON_13460<br>PCON_02131<br>PCON_08266<br>PCON_03101 (only 246 aa)<br>PCON_07564 (only 181aa)<br>PCON_03584 (only 168 aa)<br>PCON_04154 (only 267 aa)<br>PCON_04718 (only 226 aa)	5e-052<br>1e-038<br>3e-038<br>1e-037<br>7e-037<br>2e-029<br>2e-029<br>4e-027<br>8e-026<br>3e-019<br>2e-015<br>2e-009<br>6e-009<br>1e-008<br>2e-008<br>6e-007<br>3e-005<br>1e-004<br>2e-004<br>2e-004
het-r<br>ACM48730.1	[10]	non-allelic *het* gene against *het-v*, WD repeat doamin, TOL/HET-6/HET-E domain, GTP-binding	PCON_05683 (ribos. assem.)<br>PCON_10921 (rco-1)<br>PCON_04786<br>PCON_11858<br>PCON_12888 (only 143 aa)<br>PCON_10803 (only 154 aa)<br>PCON_09822 (only 217 aa)<br>PCON_01716<br>PCON_10843<br>PCON_03032<br>PCON_14293<br>PCON_05422 (only 173 aa)<br>PCON_08266	1e-061<br>7e-060<br>7e-039<br>1e-033<br>2e-031<br>9e-031<br>1e-026<br>1e-025<br>3e-022<br>1e-020<br>2e-016<br>4e-015<br>2e-011

			PCON_14268<br>PCON_02131<br>PCON_13460<br>PCON_04718 (only 226 aa)<br>PCON_07564 (only 181aa)<br>PCON_03101 (only 246 aa)<br>PCON_04154 (only 267 aa)	3e-011<br>1e-008<br>6e-007<br>8e-006<br>1e-005<br>3e-004<br>0.004
het-s<br>Q03689	[11]	allelic *het* gene, prion analogue	PCON_05235	7e-007

References

1. Glass NL, Grotelueschen J, Metzenberg RL: ***Neurospora crassa* A mating-type region.** *Proc Natl Acad Sci U S A* 1990, **87:**4912-4916.
2. Staben C, Yanofsky C: ***Neurospora crassa a* mating-type region.** *Proc Natl Acad Sci U S A* 1990, **87:**4917-4921.
3. Saupe SJ, Kuldau GA, Smith ML, Glass NL: **The product of the het-C heterokaryon incompatibility gene of *Neurospora crassa* has characteristics of a glycine-rich cell wall protein.** *Genetics* 1996, **143:**1589-1600.
4. Kaneko I, Dementhon K, Xiang Q, Glass NL: **Nonallelic interactions between het-c and a polymorphic locus, pin-c, are essential for nonself recognition and programmed cell death in *Neurospora crassa*.** *Genetics* 2006, **172:**1545-1555.
5. Smith ML, Micali OC, Hubbard SP, Mir-Rashed N, Jacobson DJ, Glass NL: **Vegetative incompatibility in the het-6 region of *Neurospora crassa* is mediated by two linked genes.** *Genetics* 2000, **155:**1095-1104.
6. Shiu PK, Glass NL: **Molecular characterization of tol, a mediator of mating-type-associated vegetative incompatibility in *Neurospora crassa*.** *Genetics* 1999, **151:**545-555.
7. Xiang Q, Glass NL: **Identification of vib-1, a locus involved in vegetative incompatibility mediated by het-c in *Neurospora crassa*.** *Genetics* 2002, **162:**89-101.
8. Saupe S, Descamps C, Turcq B, Bégueret J: **Inactivation of the *Podospora anserina* vegetative incompatibility locus het-c, whose product resembles a glycolipid transfer protein, drastically impairs ascospore production.** *Proc Natl Acad Sci U S A* 1994, **91:**5927-5931.
9. Espagne E, Balhadère P, Penin ML, Barreau C, Turcq B: **HET-E and HET-D belong to a new subfamily of WD40 proteins involved in vegetative incompatibility specificity in the fungus *Podospora anserina*.** *Genetics* 2002, **161:**71-81.
10. Chevanne D, Bastiaans E, Debets A, Saupe SJ, Clavé C, Paoletti M: **Identification of the het-r vegetative incompatibility gene of *Podospora anserina* as a member of the fast evolving HNWD gene family.** *Curr Genet* 2009, **55:**93-102.
11. Deleu C, Clavé C, Bégueret J: **A single amino acid difference is sufficient to elicit vegetative incompatibility in the fungus *Podospora anserina*.** *Genetics* 1993, **135:**45-52.
