# Supplementary material for: The Genome and Development-Dependent Transcriptomes of Pyronema confluens: A Window into Fungal Evolution
Source: PLoS Genet. 2013 Sep 19;9(9):e1003820. doi: 10.1371/journal.pgen.1003820 (PMC3778014; doi:10.1371/journal.pgen.1003820)
Supplement: Table S15 — Oligonucleotides used as primers for qRT-PCR analysis. (PDF) [file pgen.1003820.s031.pdf]

Table S15. Oligonucleotides used as primers for qRT-PCR and RT-PCR analysis.

Oligonucleotide	sequence 5'-3'
PCON_02619-for	TGTCCGAGCCTCGTCTGTTA
PCON_02619-rev	CAAGGCTGGGTCCAAATAGC
PCON_04108-for	CGAGGGACGGAGGATGTAAG
PCON_04108-rev	TAGCATCTGCCTCAGCGGTA
PCON_06047-for	CAGGCCAGAGACGAAGAGGT
PCON_06047-rev	TAATTTACCCCGCCACCAAC
PCON_06720-for	TGCAGTTCTCCACCATCGTC
PCON_06720-rev	ACAGTGCAGTCGTTGGCATC
PCON_06721-for	TCGATTGCACGAGCGAGTAT
PCON_06721-rev	TTCTCGAATCGGGAGGTGTT
PCON_09794-for	CCTCAACCAACCACCAACAA
PCON_09794-rev	GCGAGGAGGAAGTGCTTGTC
PCON_09947-for	CCCGTCGAGAAATGTCCAAT
PCON_09947-rev	TGCTTCGGAGAAAAGTGCAA
PCON_04765-for	AACAGCCTCAAACCGCAACT
PCON_04765-rev	GTCTGAAACGACGGGACCTC
PCON_10922-for	ACCACTAGCGGTGGGGATAA
PCON_10922-rev	GACGGAAGCTGTTGCTGTTG
PCON_10987-for	CCGCCATGTCAGAATACGAA
PCON_10987-rev	GCTCCTTGGCTTGGATCAAC
PCON_10990-for	GGGTTATGGAAGGCGTTGAA
PCON_10990-rev	CTTTCCGACAGCATGATCCA
PcWC1-qRT-fw	GTGAGCCGTAGTGCTTCTCG
PcWC1-qRT-rv	GAGATGGCGTTCATGGATTG
PcWC2-qRT-fw	ACCGGCGAAACCTACAAGTC
PcWC2-qRT-rv	CAATATGCGTCCCGTCAAAC
PcFRQ-qRT-fw	CTGGGTCGGAGGATCAGACT
PcFRQ-qRT-rv	GCCGCAATAAGTCCCTCAAG
PCON_09365_t1	ctggacatcctgtggtgccatcg
PCON_09365_t3	caagcatcgattgctacaaagacc
PcCRY-qRT-fw	CTCGGAGGATGGGAGGATTA
PcCRY-qRT-rv	TCAACCCGTTCCTTCTTCG
PcAL1-qRT-fw	CGCAGGGTCGAAGATTACCT
PcAL1-qRT-rv	CTCCTTCGCCCTAAGGTTCA
PcAL2-qRT-fw	TACATTCCGGGGACTTGGTT
PcAL2-qRT-rv	CAGCTTCAGCCAGCAGTTTC
PcAL3-qRT-fw	GAGAAGAGAACGGGCAGCTT
PcAL3-qRT-rv	CCAGAATCCCCATCAACATCT
PcPHY1-qRT-fw	ACGGTGGTACATCAGCAACC
PcPHY1-qRT-rv	TACTTCTGGGCCTTCCGAGT
PcPHY2-qRT-fw	CCAATGGTCTCGAATGCCTA
PcPHY2-qRT-rv	CTGGCATCAGCAAGAGCTTC
PcORP1-qRT-fw	TTTGGACGTCTGTTCCCAAG
PcORP1-qRT-rv	TAACCCTCAACGGCATTCTG
