# Supplementary material for: The Genome and Development-Dependent Transcriptomes of Pyronema confluens: A Window into Fungal Evolution
Source: PLoS Genet. 2013 Sep 19;9(9):e1003820. doi: 10.1371/journal.pgen.1003820 (PMC3778014; doi:10.1371/journal.pgen.1003820)
Supplement: Text S1 — Distribution of gene expression levels. (PDF) [file pgen.1003820.s032.pdf]

Text S1. Distribution of gene expression levels.

The availability of transcriptome data for *P. confluens* also allowed us to compare overall expression levels across the genome. In a previous study with metazoans, genes could be grouped in two classes with high and low expression levels, respectively, independent of species, tissue type, or type of experiment (microarrays, RNA-seq) [1]. Strongly expressed genes were considered to comprise the active and functional transcriptome while weakly expressed genes in each cell type show "leaky" expression. In fungi, the analysis of RNA-seq data from the filamentous ascomycete *Sordaria macrospora* indicated that the situation might be different, because there were up to three expression peaks depending on the conditions analyzed [2]. However, *S. macrospora* was the only fungus for which this analysis has been performed yet. Therefore, we analyzed genome-wide expression levels in *P. confluens* (Figure S4). Interestingly, the situation in *P. confluens* is more similar to metazoans, with two main peaks that represent high and low expression in all three conditions investigated (sex, DD, vegmix). At this point, it is not clear whether this is the rule or the exception in fungi, and further analysis with more species and conditions will be necessary to address this question. An analysis of the genes that belong to the different peaks in the three conditions showed that the majority is either in the low or high peak, independent of the condition (Figure S4B). Among those genes that occur in either peak in only one or two conditions, the highest number (1,276 genes) is in the high peak in condition sex, and in the low peak in conditions DD and vegmix, therefore these genes might be activated specifically during sexual development. An examination of the putative functions of these 1,276 genes showed that the majority (76%) are orphan genes, compared to only 46% of orphan genes among all predicted genes. This is consistent with the finding that many orphan genes are preferentially expressed during sexual development (see main manuscript).

1. Hebenstreit D, Fang M, Gu M, Charoensawan V, van Oudenaarden A, et al. (2011) RNA sequencing reveals two major classes of gene expression levels in metazoan cells. Mol Syst Biol 7: 497.
2. Teichert I, Wolff G, Kück U, Nowrousian M (2012) Combining laser microdissection and RNA-seq to chart the transcriptional landscape of fungal development. BMC Genomics 13: 511.
