# Supplementary material for: The Genome and Development-Dependent Transcriptomes of Pyronema confluens: A Window into Fungal Evolution
Source: PLoS Genet. 2013 Sep 19;9(9):e1003820. doi: 10.1371/journal.pgen.1003820 (PMC3778014; doi:10.1371/journal.pgen.1003820)
Supplement: Text S2 — Genes for pheromone/receptor signaling. (PDF) [file pgen.1003820.s033.pdf]

Text S2. Genes for pheromone/receptor signaling

A BLAST search of the *P. confluens* genome with the *S. cerevisiae* alpha-factor receptor Ste2 and the a-factor receptor Ste3 led to the identification of two genes encoding a peptide receptor (*PCON_02097*) and a lipopeptide receptor (*PCON_08875*). The deduced gene products are putative seven transmembrane domain proteins with a predicted extracellular N-terminal tail, three outer and three cytoplasmic loops, and an inner C-terminal tail (http://www.enzim.hu/hmmtop/html/submit.html). The proteins are therefore highly similar to G-protein coupled pheromone receptors functionally characterized in other filamentous ascomycetes [1-4]. Interestingly, transcripts of both genes are highly upregulated during conditions that allow sexual development, in contrast to most other genes in the mating type/pheromone system that are only weakly or not differentially regulated at the level of transcription (Table S7).

BLASTP and TBLASTN searches with peptide and lipopeptide pheromones of yeasts and filamentous ascomycetes yielded no significant hits, similar to searches with pheromone consensus sequences (data not shown). However, enzymes involved in the processing of pheromone precursors are well conserved in *P. confluens* (Table S7). Thus, sequences of *P. confluens* pheromone precursors, if present, seem to be rather different from the known pheromone precursors of ascomycetes.

In *S. cerevisiae*, after binding of the pheromones to a cell-type-specific receptor, the signal is transmitted by interaction of a heterotrimeric G protein composed of Gα (Gpa1p), Gβ (Ste4p), and Gγ (Ste18p) through a downstream mitogen activated protein (MAP) kinase cascade encoded by STE20, STE11, STE7, and FUS3. Homologues of all these genes and most other conserved genes of the *S. cerevisiae* pheromone response pathway have been identified in *P. confluens* (Table S7). The identification of conserved components of the pheromone response pathway, pheromone processing enzymes and pheromone receptors suggests that *P. confluens* encodes pheromones and has the potential to trigger a pheromone-induced G protein-linked signal transduction pathway. We also identified homologs to the proteins of the two other MAP kinase signaling modules that are present in filamentous ascomycetes as well as homologs to known developmental genes in the more derived filamentous ascomycete *S. macrospora* indicating that core signaling and developmental pathways might be conserved from lower to higher filamentous ascomycetes (Table S7, see also section about comparative expression analysis in the main manuscript).

1. Kim H, Borkovich KA (2006) Pheromones are essential for male fertility and sufficient to direct chemotropic polarized growth of trichogynes during mating in *Neurospora crassa*. Eukaryot Cell 5: 544-554.
2. Lee J, Leslie JF, Bowden RL (2008) Expression and function of sex pheromones and receptors in the homothallic ascomycete *Gibberella zeae*. Eukaryot Cell 7: 1211-1221.
3. Mayrhofer S, Weber JM, Pöggeler S (2006) Pheromones and pheromone receptors are required for proper sexual development in the homothallic ascomycete *Sordaria macrospora*. Genetics 172: 1521-1533.
4. Pöggeler S (2011) Function and evolution of pheromones and pheromone receptors in filamentous ascomycetes. In: Pöggeler S, Wöstemeyer J, editors. The Mycota Evolution of fungi and fungal-like organisms. Berlin, Heidelberg: Springer-Verlag.
